# Hunting and processing of straight-tusked elephants 125.000 years ago: Implications for Neanderthal behavior

**DOI:** 10.1126/sciadv.add8186

**Published:** 2023-02-01

**Authors:** Sabine Gaudzinski-Windheuser, Lutz Kindler, Katharine MacDonald, Wil Roebroeks

**Affiliations:** ^1^ MONREPOS Archaeological Research Center and Museum for Human Behavioral Evolution, Schloss Monrepos, Neuwied 56567, Germany (LEIZA).; ^2^ Institute of Ancient Studies, Pre- and Protohistoric Archaeology, Johannes Gutenberg University of Mainz, Schönborner Hof, Schillerstraße 11, Mainz 55116, Germany.; ^3^ Faculty of Archaeology, Leiden University, P.O. Box 9514, 2300 RA Leiden, Netherlands.

## Abstract

Straight-tusked elephants (*Palaeoloxodon antiquus*) were the largest terrestrial mammals of the Pleistocene, present in Eurasian landscapes between 800,000 and 100,000 years ago. The occasional co-occurrence of their skeletal remains with stone tools has generated rich speculation about the nature of interactions between these elephants and Pleistocene humans: Did hominins scavenge on elephants that died a natural death or maybe even hunt some individuals? Our archaeozoological study of the largest *P. antiquus* assemblage known, excavated from 125,000-year-old lake deposits in Germany, shows that hunting of elephants weighing up to 13 metric tons was part of the cultural repertoire of Last Interglacial Neanderthals there, over >2000 years, many dozens of generations. The intensity and nutritional yields of these well-documented butchering activities, combined with previously reported data from this Neumark-Nord site complex, suggest that Neanderthals were less mobile and operated within social units substantially larger than commonly envisaged.

## Teaser

Neanderthals hunted the largest terrestrial mammals 125,000 years ago.

## INTRODUCTION

Straight-tusked elephants (*Palaeoloxodon antiquus*) were the largest terrestrial mammals of the Pleistocene, with body mass estimates of up to 6 metric tons for adult females and up to 13 metric tons for adult males, i.e., roughly three times larger than that of living Asian elephants, twice that of African ones, and also much larger than mammoth (*Mammuthus primigenius*) (*1*). With shoulder heights of up to 4 m and with their large tusks, they were very impressive animals (*1*), present in the landscapes of Pleistocene Europe and western Asia from about 800 thousand years (ka) ago until c. 100 ka ago. *P. antiquus* had a preference for warm temperate settings and has been documented in the middle latitudes of Europe mainly during interglacials, probably finding a refuge in the southern parts of western Eurasia during colder parts of the Pleistocene (*2*, *3*). Their spatiotemporal distribution overlapped with that of western Eurasian hominins, such as Neanderthals and earlier populations. Several Lower and Middle Paleolithic sites have yielded skeletal remains of these large elephants, in spatial association with stone tools [e.g., (*4*, *5*)], leading to rich speculation about the nature of interactions between these proboscideans and Pleistocene humans: Were these the remains of scavenged animals or may some of them have been hunted, although hunting of these large animals is often considered a dangerous enterprise, with the costs possibly larger than the benefits [e.g., (*6*)]? In addition, in the latter case, given the quantity of fat and meat involved, does this imply a role for larger groups or the availability of methods to store these food items over time?

A number of Last Interglacial sites from central Europe, the focus area of this study, have been featured in these discussions. On the basis of the rich *P. antiquus* material from the Last Interglacial travertine exposures at Taubach (Germany) [minimum number of individuals (MNI), 64], Soergel (*7*) suggested back in 1922 that Neanderthals were targeting young (<20 years old) individuals, hunting them in pit traps there. In 1948, the Last Interglacial site Lehringen (Germany) yielded a skeleton of *P. antiquus*, associated with 25 flint artifacts and a wooden lance (*8*), while a small number of flint artifacts (*n* = 27) were recovered during excavation of an adult (c. 40 years old) *P. antiquus* individual from a Last Interglacial lake infill at Gröbern (Germany) in 1987 (*9*).

However, none of these three Last Interglacial elephant locales have yielded bones with unambiguous cut marks, which would provide the most straightforward evidence for elephant exploitation by humans. As concluded in a recent review of the European evidence (*10*), proboscidean remains from Lower and Middle Paleolithic sites only yielded possible cut marks at one-third of the 36 locales studied and, if any, in very small numbers only [see also table 8 of (*11*) for Pleistocene proboscidean sites with bone surface modification described as created during carcass butchering by hominins]. One explanation for this rarity is that cut marks are rarely produced during the exploitation of large carcasses, with, for example, large muscle masses, cartilage, tendons, and strong ligaments hampering contact between stone tool edges and bone surfaces (*12*, *13*). This rarity of unambiguous anthropogenic bone surface modifications limits our understanding of the character and the nutritional importance of Pleistocene human interactions with elephants, as the spatial associations between their skeletal remains and lithic artifacts form indirect evidence only. Stable isotope analyses, on the other hand, have suggested very high amounts of mammoth meat consumption by Late Pleistocene Neanderthals and early modern humans (*14*, *15*), stressing the potential importance of proboscideans in hominin diets during the second half of the Last Glacial, i.e., from around 60 to 70 ka ago onward.

Here, we report the results of an archaeozoological study of the richest straight-tusked elephant assemblage known thus far, consisting of the well-preserved remains of >70 *P. antiquus* individuals (*3*) recovered during archaeological rescue operations carried out by D. Mania and his team from 1985 to 1996 at the Last Interglacial (Eemian) site Neumark-Nord 1, near Halle, in central Germany. As we show here, the evidence for human interference with these *P. antiquus* individuals consists of a uniquely rich cut mark record, demonstrating extended processing of a very specific part of a *P. antiquus* population, especially during the early phase of the Eemian. These results have important implications for our views of the subsistence strategies and possibly social organization of Last Interglacial Neanderthals.

Substantial parts of the Neumark-Nord record have already been published in some detail, summarized here in a brief introduction to the geological setting of the site complex, the local environment, and the traces of Neanderthal activities there, focusing on those aspects of the rich record needed to contextualize our archaeozoological study. This is followed by Results, in which we present data on the Neumark-Nord 1 *P. antiquus* assemblage. In Discussion, we interpret these results in terms of Neanderthal subsistence strategies, first, within the context of the Neumark-Nord data and, second, within the broader record of Neanderthal subsistence. We lastly also hypothesize what our data may imply for existing views on the scales of Neanderthal societies and their inferred highly mobile lifestyles.

### The Neumark-Nord site complex

**Fig. 1.**
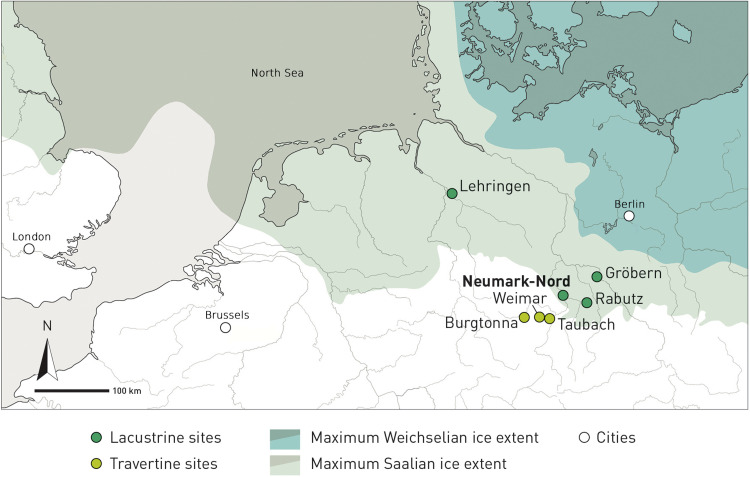
Location of Neumark-Nord and other Last Interglacial archaeological sites on the northern European plain, relative to the maximum extension of the Saalian and Weichselian glaciers. Just south of the Saalian glaciated area, where Neumark-Nord is located, occurs a rich series of Last Interglacial travertine sites.

Neumark-Nord is located about 10 km south of the German city of Halle, in an area that was covered by Saalian ice sheets at the end of the Middle Pleistocene, during marine isotope stage (MIS) 6 but that was not reached by the ice advance of the subsequent (Weichselian) glaciation (Fig. 1). The location in between the maximum extensions of the MIS 6 and MIS 2 glaciers is relevant for understanding the exceptional quality and quantity of well-preserved Last Interglacial sequences in this part of Europe. The retreat of the Saalian ice sheets exposed a landscape covered in sediment-receiving structures caused by a range of glacial and postglacial processes. The water bodies that developed in these landscapes filled up with sediment during the Last Interglacial. Covered by thick wind-blown deposits during the Weichselian, these basins provide a uniquely rich palaeoenvironmental (and sometimes archaeological) record for the Last Interglacial, a period that is not well represented elsewhere in the European sedimentary record south of the Saalian-glaciated area.

The Last Interglacial basins of Neumark-Nord are located in the former lignite open cast mine Mücheln in Saxony-Anhalt, Germany (51°19′28″N, 11°53′56″E). The first basin, Neumark-Nord 1, was uncovered in 1985. D. Mania and his research group thoroughly investigated this basin in a series of rescue interventions, in a continuous race against the large-scale bucket-wheel excavators, until the end of the mining activities in the mid-90s. During subsequent reclamation works in the quarry, Mania also found the second basin, Neumark-Nord 2, about 100 m northeast of Neumark-Nord 1. Multidisciplinary investigations and archaeological excavations of Neumark-Nord 2 were undertaken from 2004 to 2009. In the area around the former lignite quarry, more basin structures similar to Neumark-Nord 1 and 2 are known, as part of the extended Last Interglacial lake area there (*16*).

Lake basin Neumark-Nord 1 covered an area of about 24 ha, while basin Neumark-Nord 2 represents a small and shallow pond of about 1.6 ha in size. Sandwiched between a thick Saalian till at the bottom and Weichselian loess deposits on top, both basin infills cover the complete Last Interglacial and contain uniquely high-resolution palaeoenvironmental proxies. In Northern Europe, the duration of the Last Interglacial (Eemian) is calculated to c. 11,000 years, subdivided into distinct pollen assemblage zones (PAZs) reflecting the vegetation succession during the interglacial. On the basis of stratigraphic and geochronologic position, palaeobotanical data, and alternating lake trans- and regressions during the individual PAZs, bone-bearing deposits with archaeological locales and high-density concentrations at Neumark-Nord 1 and Neumark-Nord 2 can be synchronized in high spatial and temporal resolution (*17*).

Neanderthals created a rich archaeological record here, leaving a wide variety of traces of their presence in this lake-dotted landscape. The bulk of the material dates to the early parts of the Last Interglacial, PAZ III, and PAZ IV, with a total duration of c. 2850 years, and there are some scattered traces of their presence recorded for the *Carpinus* (hornbeam) phase (PAZ V) of the Last Interglacial, with a duration of c. 4 ka. Apart from isolated bone and lithic scatters throughout the Eemian deposits, three main find contexts characterize the Neumark paleontological and archaeological record: (i) In littoral zones of the basins, associations of lithics and highly fragmented bones are present in high-density concentrations; (ii) also in littoral zones, partly articulated and disarticulated bones from single individual skeletons, partially associated with lithics, are distributed over small areas; and (iii) in gyttja deposits inside the Neumark-Nord 1 basin, articulated skeletons, partially preserved skeletons of single individuals, and skeletal remains from multiple individuals are found. Lithics were generally not documented in these gyttja deposits, but some skeletal remains display cut marks and hunting lesions (*18*).

At Neumark-Nord 2, find level NN2/2B represents one such high-density, c. 500-m^2^ large concentration, which formed along the northern margin during a lake transgression phase, with about 20,000 flint artifacts and more than 118,000 well-preserved faunal remains. The heavily cut-marked and fragmented faunal remains represent a minimum number of 166 large mammals, mostly horses (MNI, 56), bovids (MNI, 40), and cervids (MNI, 53), deposited during a time interval of 455 years maximum, as calculated from sedimentation rates during PAZ IV (*19*). Seasonality data, obtained through the analysis of faunal remains from fetal to subadult individuals from four different taxa, demonstrate that Neanderthals were active at the Neumark-Nord 2 location all year round (*17*). Together with a virtual absence of carnivore marks on the rich faunal remains from Neumark-Nord 2, this strongly suggests some form of permanent presence of Neanderthals and points to their dominant role as apex predator in the faunal community around this locale (*17*).

During intermediate regression, phases up to 100 m of broad littoral zones developed in the Neumark-Nord 1 basin, with the lower littoral zone (unit 6.1) and the upper littoral zone especially well documented. In both units, high-density concentrations, similar to NN2/2B, were encountered but could not be excavated in detail because of active mining there. These concentrations are characterized by the presence of highly fragmented animal bones, flint knapping waste, flint cores, and charcoal particles, with large chunks of charcoal. In addition, especially in unit 6.1, along the immediate shore, clusters of disarticulated and partly articulated carcasses were uncovered by the rotary extractors. In some instances, the presence of simple flakes suggests Neanderthal involvement in the disarticulation of the carcasses. Estimates of sedimentation rates, development of peat layers and counting of annual rings in tree stumps indicate that the lower littoral zone (unit 6.1) at Neumark-Nord 1 existed for c. 300 years (*20*). The majority of the elephant remains uncovered at Neumark-Nord 1 belong to unit 6.1.

All these finds were an integral part of a larger Neumark-Nord lake landscape that was extensively exploited by hominins, with the Neumark-Nord 1 and 2 archaeology representing a small sample of the evidence of hominin activities once scattered around this lake-dotted landscape. This perspective is also supported by abundant evidence for fire use at and around the excavated areas, including heated lithics, burnt bones, charred seeds, and charcoal particles, while systematic sampling of a large section through the Neumark-Nord 2 basin yielded thousands of charcoal particles and more charred seeds. An anthropogenic origin for the macroscopic charcoal from this basin area is supported by the correlations between the artifact and charcoal datasets (*21*, *22*). The distinctive charcoal peak in the lower part of the Neumark-Nord 2 section correlates with the first documented presence of archaeological finds in the two Neumark-Nord basins, reflecting the arrival of Neanderthals in the wider lake landscape during PAZ III of the Last Interglacial. This spike, with 10 times the amount of charcoal particles of any other peak in the Neumark-Nord 2 sequence, co-occurs with a strong increase in nonarboreal pollen, i.e., an opening up of the landscape, providing space for oak and hazel and their edible hazelnuts and acorns. The hypothesis that this charcoal peak might reflect anthropogenic burning of parts of the landscape was systematically addressed and evaluated in a recent study (*22*). Among the factors that shaped vegetation structure and succession in this lake landscape, this study identified a distinct ecological footprint of hominin activities, including fire use: At Neumark-Nord, notably open vegetation that started with fire events coincides with a virtually continuous c. 2000-year-long hominin presence. With an age of c. 125,000 years, Neumark-Nord provides a very early example of a hominin role in local-scale vegetation transformation caused by an archaeologically very visible and long-lasting presence of Neanderthals in this lake-land area. The analysis of the *P. antiquus* assemblage presented here provides new data regarding the character of Neanderthal activities in this larger Neumark-Nord lake landscape.

### The *P. antiquus* assemblage from Neumark-Nord 1

Mania’s team collected 3122 elephant remains during their interventions in the Neumark-Nord 1 exposures, and all of these were analyzed in the present study. The remains were recovered from three fossiliferous units in the Neumark-Nord infill, straddling the *Pinus-Quercus*, *Quercus-Corylus*, and early stages of the *Carpinus* phase (PAZ III to PAZ V) of the Last Interglacial: the lower gyttja of unit 4; the unit 6 middle gyttja, comprising the lower littoral zone 6.1 and the upper littoral zone; and the upper gyttja of unit 7.

As described in detail in (*23*), some elephants were represented by virtually complete skeletons, well preserved with bones in anatomical connection—two individuals even with partially preserved gut content—or just slightly disarticulated, while other individuals occurred in larger bone concentrations, Mania’s so-called Knochenfelder (i.e., bone fields or, here, bone complexes). These bone complexes contained the scattered remains of one or more elephants, occasionally associated with the isolated remains of other large mammals, such as rhinos, bovids, and horses. Later paleontological studies (*1*, *3*, *24*) showed that, in some cases, Mania’s single-carcass assemblages contained the remains of more than one elephant individual. Thus, in this study, we address all excavated assemblages as “bone complexes.” Many of these complexes were still in primary context when uncovered by Mania, and, in some cases, flint artifacts were associated and recovered during these rescue archaeology interventions. Some skeletons were damaged by large digging machines and/or partially destroyed before Mania’s team was able to recover any finds. Given the rescue archaeology character of the operations in the quarry, the large *P. antiquus* assemblage does not include all the skeletal remains once present around the lake, as an unknown quantity of remains went undiscovered or were destroyed through mining. The incomplete character of bone complexes may—to a limited degree—also have been caused by Neanderthal movement of skeletal elements, which is indicated by the occurrence of artificial concentrations (*n* = 9) of tusks around the lake in the lower littoral zone (unit 6.1) (*23*). In one remarkable case (E28), this concerned an isolated occurrence of nine medium- and large-sized tusks over an area of 10 m by 20 m at the southern margin of the lake without any other associated skeletal remains (*23*).

**Fig. 2.**
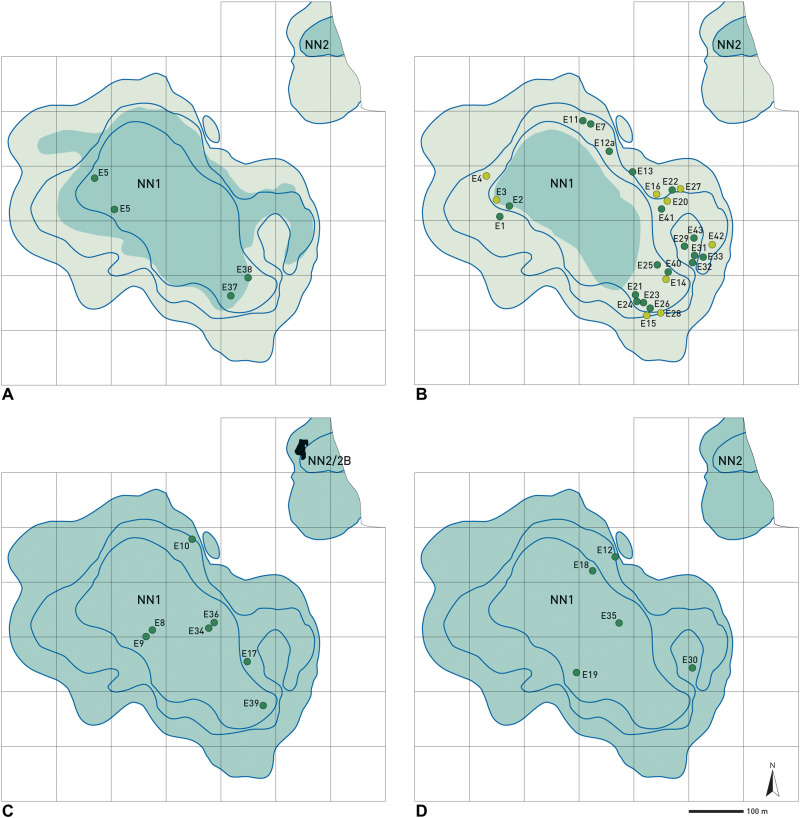
Chronostratigraphic position and location of elephant bone complexes from Neumark-Nord. NN1, basin Neumark-Nord 1; NN2, basin Neumark-Nord 2. The blue contour lines indicate the shape of the basins at, respectively, 8, 16, and 24 m below the modern day surface. Dark green dots indicate skeletal remains of one or multiple individuals; light green dots indicate isolated tusk(s) occurrences. The grid system consists of 100 m by 100 m. (**A**) Unit 4. Eemian PAZ III (duration, ~450 years), lake transgression with maximum expansion of water table indicated in dark green and littoral zone in light green. (**B**) Unit 6.1 (lower littoral zone) during Eemian PAZ IVa, a short regression phase with minimum expansion of water table indicated in dark green and littoral zone in light green. Formation duration, ~300 years. (**C**) Unit 6. Eemian PAZ IVa (duration, 1200 years), lake transgression with maximum expansion of water table indicated in green. Indicated in basin NN2 is the excavation area of the contemporaneous level NN2/2B, with a formation duration of ~450 years. (**D**) Unit 7. Eemian PAZ V (duration, 4000 years), lake transgression with maximum expansion of water table indicated in green. Maps redrawn and modified after (*17*, *29*). Subdivision and attribution of PAZ after (*58*) and duration of each PAZ after (*25*).

The stratigraphical provenance of elephant remains through the Neumark-Nord 1 Eemian sequence and their spatial distribution within the Neumark-Nord 1 basin, as recorded by Mania (*23*), are shown in Fig. 2. The elephant remains are grouped in bone complexes E1 to E43, containing the remains of one up to eight individuals. Most individuals (*n* = 36, from 15 bone complexes) were retrieved from unit 6.1 sediments, the so-called “lower littoral zone,” formed during a strong regression of the lake. The presence of swampy/muddy areas may have acted as traps and led to burial, rapid enough to prevent development of traces of surface disintegration on the bones and, thus, fostering preservation. Unit 6 also contained an upper littoral zone (Obere Uferzone), similar to the lower one formed during PAZ IV of the Last Interglacial, with an estimated total duration of ~2400 years (*25*).

Various previous studies have dealt with the straight-tusked elephant assemblage from Neumark-Nord, including a genetic analysis, which showed that *P. antiquus* was a close relative of extant African forest elephants (*26*). Palombo and colleagues (*1*, *3*, *24*) performed a very thorough and extensive analysis of the Neumark-Nord 1 *P. antiquus* remains from a paleontological perspective, in which they focused on the structural characteristics of the assemblage: body size and shape, age at death, sexual dimorphism, and ontogeny (*1*, *3*, *24*). Body size estimates and study of the features indicative of the sex of the individuals demonstrated a very pronounced sexual dimorphism, with marked differences in height and body mass. For 35 individuals, they could confidently determine age at death: Unexpectedly, they found that 94.3% of the individuals were older than 25, and 40% were older than 40 years. Juveniles or young adults only made up 5.7%, matching the proportion of very old (>50 years) individuals in the assemblage. Size and bone structure of individuals who they were not able to assign an age, again, were not indicative of young animals. The age distribution from unit 6.1 main find level (with 839 remains studied) had similar proportions (96.9%, >20 years; 40.6%, >40 years; 6.2%, >50 years), again indicating a “strongly unbalanced” mortality profile (*24*) not observed so far in either fossil or recent elephant populations and differing substantially from the so-called “catastrophic” and “attritional” profiles. Other unexpected results were the high percentages of males in all levels, likewise suggesting a selective accumulation. Palombo and colleagues [(*3*), p. 244] emphasized that the age and sex structure “…inferred for the bone assemblage is actually different from that of the population that inhabited the region over the time the fossiliferous sediments accumulated.”

Palombo and colleagues (*3*) were unable to explain the causal factors underlying the very peculiar age and sex structure that they had identified. Our archaeological study of the assemblage, which has profited in many ways from the analyses by Palombo and colleagues, does offer a clear explanation for this peculiar selection: Neanderthal hunting.

## RESULTS

In his presentation of the evidence from Neumark-Nord 1, Mania (*23*) lists a total of 44 bone complexes identified in the field, comprising 35 complexes with skeletal remains of one or more individuals and nine separated depositions of tusks from unit 6.1. For eight complexes, we could not recover any finds in the Halle Museum. For some of these (*n* = 5), Mania explicitly documented that they could not be rescued in the field, and we assume that this was the case for all eight. For four other complexes, only the recovery of some skull/molar fragments was possible.

A total of 27 bone complexes yielded cranial and postcranial material that could be examined for surface modifications (see Table 1 and tables S1 and S2). Some of these remains had suffered recent damage by the bucket excavators during mining activities. As the bones were uncovered in toto, most of the modern fractures could be conjoined. In cases where conjoining was not possible, irregular fracture planes, white in color, clearly indicated that these fractures occurred when the bones were already fossilized.

**Table 1.** Stratigraphical provenance of all 44 elephant bone complexes from Neumark-Nord 1. For a broader contextualization of these bone complexes, see table S15.

Unit	Elephant bone complex (EBC)	Comments
4	E37, E38	Not recovered
	E5	Largely destroyed, skull fragments only, cut marked assemblage
	E6	Cut marked assemblage
6	E8, E9, E10, E17, E34, E36, E39	Cut marked assemblages
6.1	E7	Largely destroyed,
		Skull fragments only
	E2, E11, E12a, E41	Not recovered
		(resp. bones lack attribution to EBC)
	E3, E4, E14, E15, E16, E20, E27,	Tusk(s) only
	E28, E42	
	E1, E13, E21, E22, E23, E24, E26,	Cut marked assemblages
	E29, E32, E33, E40, E43	
	E25, E31	Unmodified assemblages
7	E30	Cut marked assemblage
	E19, E35	Largely destroyed
	E12, E18	Not recovered

The 27 complexes studied originate from units 4, 6.1 (the main find horizon), 6, and 7 and often contain the remains of more than one elephant individual. Complexes E5 and E6 with two individuals were studied from unit 4. Ten individuals were analyzed from seven bone complexes from unit 6, 36 individuals from unit 6.1; the lower littoral zone within unit 6 originate from 15 bone complexes (*24*); and, last, four individuals from bone complexes E19, E30, and E35 were studied from unit 7.

### Bone surface modifications: Nonanthropogenic

The overwhelming majority of skeletal elements did not display any signs of climatically induced weathering (*27*, *28*), suggesting rapid burial of the skeletal elements. Only in a few cases, signs of weathering stages 0-1 and 1 (*28*) [see (*3*): figure 23 for femora from E43 and E39, figure 24 for pelvis from E8 and E24, and figure 28 for the scapula from E43] on one surface of individual bones indicate that a delay in the burial process occasionally occurred. Several carnivore species were documented in the Neumark-Nord fauna, among them two that regularly feed on mammal bones, i.e., *Crocuta crocuta* and *Canis lupus.*

For unit 6.1, five individuals from five bone complexes representing two to eight individuals and a single carcass (E26) displayed carnivore traces (see table S3). It is primarily the zonoskeleton that was affected here, and cut marks were observed on gnawed or tooth marked bones.

From unit 6, six singular carcasses (E8, E9, E17, E34, E36, and E39) and bone complex E10, with three individuals altogether, were documented. All but one individual (E17—represented by a skull, the fragment of a rib, and a third metacarpal) showed some carnivore modification, as did a further carcass from E10. Most traces were observed on the zonoskeleton, particularly on the thoracic spine where anthropogenic modifications were also documented for most of the carcasses. With the zonoskeleton missing, only the front legs of carcass E34 showed traces of gnawing.

Traces of carnivore modification are relatively rare, and their impact on the overall composition of the assemblage is altogether negligible. This is primarily evident from the character of traces observed, underlined by the fact that evidence for regular bone transport or bone consumption by carnivores, especially for the single-carcass findspots, is missing. Concerning the character of traces observed, only in a few cases (*n* = 8) did carnivore modification lead to the partial or almost complete destruction of proximal or distal longbone epiphyses (documented for unit 6 for E10, E34, and E39, and for 6.1 by E22, E24, and E43), while the overwhelming majority of traces is restricted to tooth marks with only minor bone destruction involved [cf. E24—thoracic spine; see (*29*), figure on p. 450 to 451].

Gnawing occurred when parts of the carcasses were still in anatomical position, as shown by tooth marked sections of the spine or feet (documented for unit 6—E8, E9, E10, and E36—and for unit 6.1—E22, E23, and E43), suggesting that carnivores spent only a relatively short time at the carcasses. We also documented evidence for late (i.e., posthuman) carnivore access: A distally completely gnawed ulna in E34, preserved including its unfused and unmarked distal epiphysis, shows that carnivore modification occurred when the distal epiphyses had already been disarticulated from the carcass. Late carnivore access is also evidenced by nonoverlapping of cut and tooth marks on the same elements, showing that carnivore access occurred after anthropogenic meat removal.

### Anthropogenic bone surface modifications

**Fig. 3.**
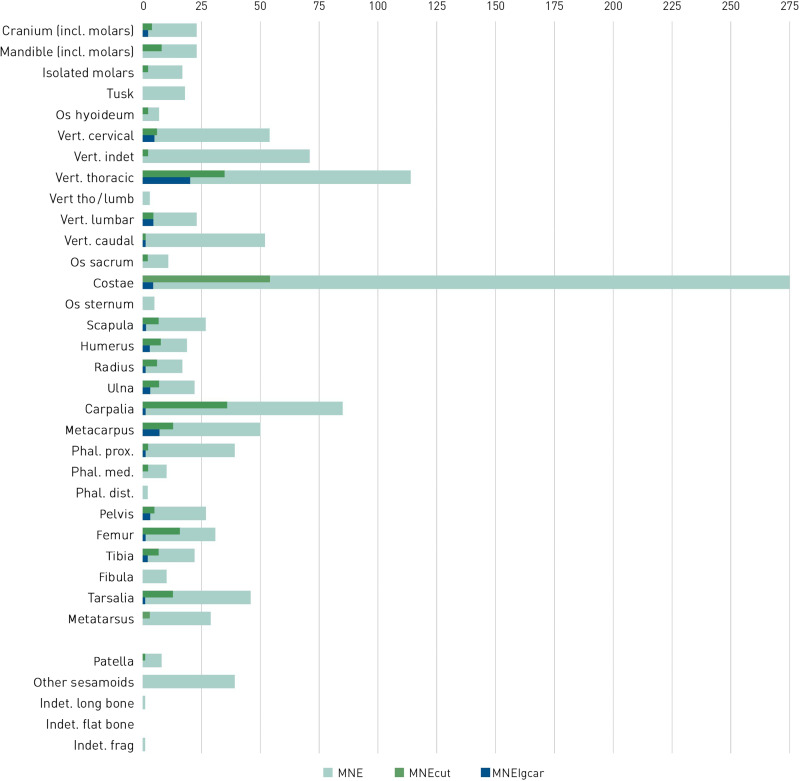
MNE (minimal number of elements) for bones of *P. antiquus* from Neumark-Nord (light green), MNE of cut-marked bones (dark green), and MNE of bones displaying damage induced by large carnivores (blue). For data, see tables S1 and S2.

**Fig. 4.**
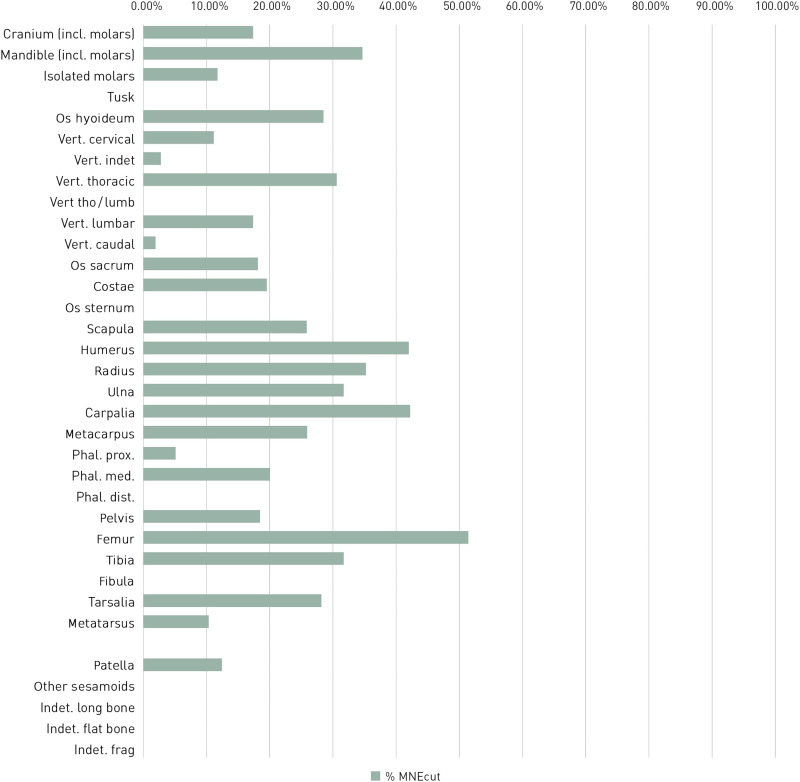
Frequency of MNE displaying cut marks on *P. antiquus* bones from Neumark-Nord 1 (*n* = 1181), in percentage per skeletal element. For data, see tables S1 and S2.

All of the studied bone complexes display traces of anthropogenic modification of elephant carcasses. Figures 3 and 4 give an overview of the cut mark distribution for the whole Neumark-Nord assemblage. The Supplementary Materials (text S1) provide detailed information about the cut mark distribution of individual carcasses from the main fossiliferous units.

The difficulties encountered in the field during rescue operations sometimes resulted in the retrieval of only a small selection of specific elephant individuals’ bones. However, the large number of individuals represented and the excellent state of preservation of the salvaged remains enabled good documentation of the distribution of a rich cut mark record over the various body parts. This demonstrates a repetitive and virtually complete exploitation of these large animals based on the following observations.

**Fig. 5.**
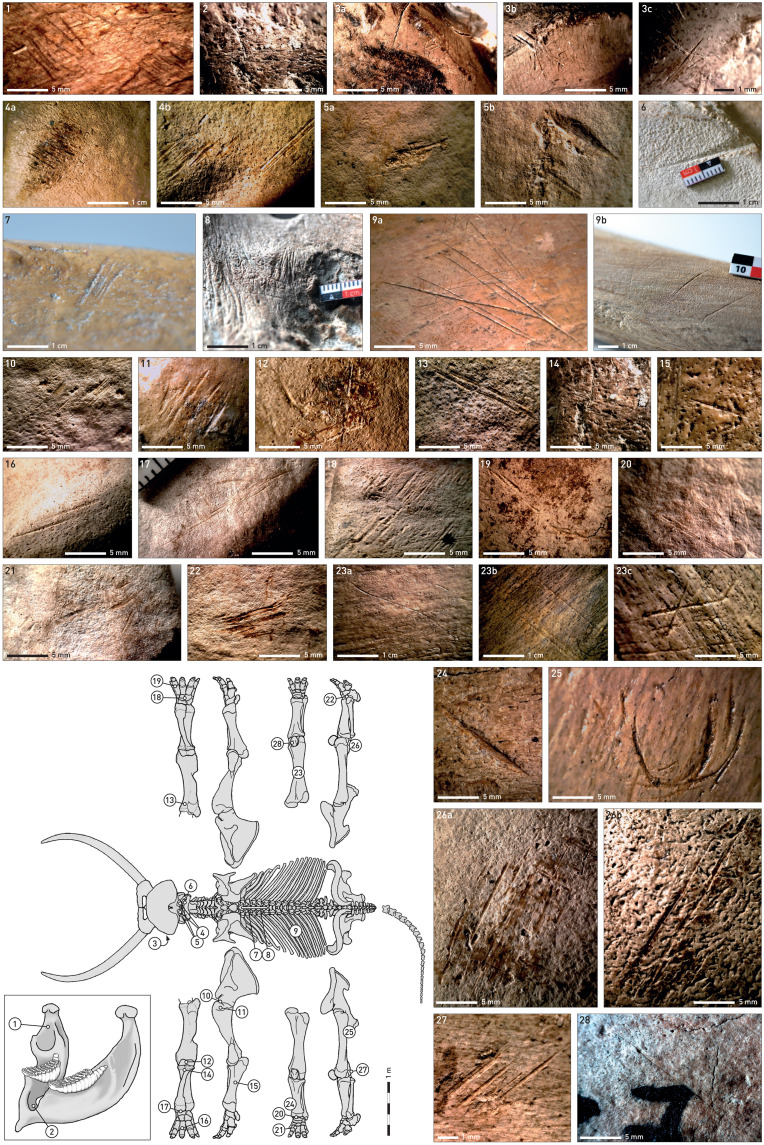
Cut marks on skeletons of *P. antiquus* from Neumark-Nord. The position of cut marks on the skeletons is indicated by the numbering. For detailed descriptions, see text. Bones of different individuals from 12 bone complexes [E1, E8, E17, E21, E22, E24 (individuals E24A, E24B, and E24D), E29, E32, E35, E36, E40, E43] served as examples.

1) There are several indications that skulls and mandibles of elephants were intensively exploited. For instance, cut marks produced by the disarticulation of the lower jaw are present in the material (e.g., in E24A and E43; Fig. 5, 1). In various bone complexes, there are also cut marks from removal of tissue from the lateral ramus mandibulae (e.g., on E24B and E24D) as well as from targeting the palate and the tongue. Thus, there are cut marks on the buccal surface of the pars incisiva of the mandible (e.g., on E1; Fig. 5, 2) and on buccal and lateral faces on isolated molars of maxillae (e.g., on E22 and E40; Fig. 5, 3) and hyoids (e.g., on E24).

2) Skulls were separated from the atlas at the condyles of the os occipitale. During this process, narrowly defined fields of cut marks appeared on the articular surfaces of the right and left condyles. These are characterized by particularly deep, intersecting cuts, with the periost still partially preserved between the cuts (e.g., on E30; Fig. 5, 4, and fig. S18A). This process of disarticulation also produced deep marks that resemble hack marks, which show that the sequence of cutting began with the tip of a stone implement being driven into the bone with force (e.g., on E24; Fig. 5, 5). Corresponding traces can be observed on the cranial articular surfaces of the atlas (e.g., on E24; Fig. 5, 6). The severing of the skull at this position allows easy access to the brain directly through the occipital opening.

3) Cut marks showing that cervical vertebrae were also disarticulated were observed on their incisura vertebralis caudalis (e.g., on E22) and around the foramen transversarium (e.g., on E24). On the majority of vertebrae, however, cuts on the processi spinosi of the thoracic vertebrae testify to the removal of backstrap muscle/tenderloin [e.g., on E8, E10 (fig. S11A), E17, E21 (Fig. 5, 7), E22, E23, and E24] either on both sides of the body or, as in the case of E10, only on one side. The traces can continue on the spinae of the lumbar vertebrae (e.g., on E10) and on those of the os sacrum (e.g., on E22 and E23; fig. S15J). The ribs were separated from the vertebrae as shown by cuts on the processus articularis on thoracic vertebrae (e.g., on E23 and E24; Fig. 5, 8) and on the caput costae of ribs [e.g., on E23 (fig. S15, D and F), E24, and E30]. Cut marks on the corpus costae (e.g., on E23; fig. S15E) indicate the removal of skin, fat, and connective tissue.

4) The outer side of the rib cage was freed from fat and connective tissue, as indicated by cuts on the lateral corpus costae of the ribs [e.g., on E10, E17 (Fig. 5, 9a), E22, E23 (fig. S15, A to C), E24, E32 (Fig. 5, 9b), E30, and E43]. These traces scatter over the outer surface of the ribs and are often long and deep. Cut marks can also be observed on the inner side of the rib cage. Thus, bone complex E10 bears witness to the fact that the ribs were still in anatomical position during this particular step of the dissection process (fig. S11B). For this, the rib cage must be opened and the viscera must be removed. The removal of viscera is also illustrated by cuts, e.g., on the ramus cranialis ossis pubis of the pelvis, also observed in bone complex E10.

5) Cut marks on the medial face of the scapula (e.g., on E22, E24, E30, E32, and E43) show the defleshing of the torso, and cuts around the articulation to the humerus (e.g., on E22, Fig. 5, 10) indicate the detachment of the scapula from the humerus. Cuts on the proximal [e.g., on E10 and E22 (Fig. 5, 11), E23 (fig. S15G), E35, and E43] and distal joints of the humerus [e.g., on E10 (fig. S11F) and E22 (Fig. 5, 12)] originate from the disarticulation of the bone. Further traces on the lateral and cranial surfaces of the entire diaphysis demonstrate defleshing (e.g., on E22; Fig. 5, 13).

6) Radius and ulna were separated from the humerus, leaving traces on the proximal joint of the ulna (e.g., on E9; fig. S7A1) before or after the defleshing of the bone, indicated by cut marks in the cranial diaphysis of the ulna [e.g., on E6 (Fig. 5, 14) and E22] and along the caudal ridge of the ulna diaphysis (e.g., on E21; Fig. 5, 15). Radius and ulna were then meticulously detached from the forelimb [cuts on distal joints, e.g., on E10 (figs. S11F and S15H) and E23], and the individual bones of the autopodium were separated from each other. This can be seen in numerous examples and was probably done to access and exploit the rich fat deposits in the elephant feet, which are tightly connected to the metapodials and phalanges [e.g., on E1, E6 (fig. S4B), E9 (fig. S7E), E10 (fig. S11, G to M), E21, E22, E23, E24 (Fig. 5, 16 to 18), E30, E36 (Fig. 5, 19), and E43] (*11*, *30*). In an equally meticulous manner, the rear lower limbs and feet were dissected [e.g., on E9 (fig. S7D), E10 (fig. S11, M and P), E24 (Fig. 5, 20 to 21)], E30 (fig. S18B), and E43 (Fig. 5, 22)]. This is true, although the connection between calcaneus and talus is very difficult to disarticulate with stone tools (*31*).

7) Cut marks on the pelvis again illustrate the defleshing of the carcasses with traces on the lateral os ilium (e.g., E10, E24, and E29) and its evisceration (cuts medial on os ilium and caudal on os pubis) (e.g., E10; fig. S11C). Similar to what has already been observed for ribs and long bones, the defleshing cut marks are long and deep and scattered over the bone surface. This is also underlined by the quality of cut marks left by the defleshing of the femur on their cranial diaphysis (e.g., in E24; Fig. 5, 23) and on the cranial and lateral midshaft of the tibia [e.g., E10 (fig. S11O) and E22 (Fig. 5, 24)]. A rather unusual cut mark was documented on the proximal, medial diaphysis of the left femur from E29 (Fig. 5, 25). As with all other long bones in this sample, cut marks particularly on the medial and lateral distal epicondyles [e.g., E8, E9 (fig. S7B), E10 (fig. S11N), E22 (Fig. 5, 26), E23 (fig. S15K), and E43 (Fig. 5, 27)] and on the proximal articular head of the femur (e.g., E23; fig. S15J) indicate its disarticulation and the deboning of the hindquarters. The dissection of a knee-joint left cut marks on a patella (e.g., E21; Fig. 5, 28). Last, cut marks on the distal epiphysis of the tibia indicate its disarticulation (e.g., on E43).

What is most notable is that cut marks occur repetitively on skeletal elements of the left and right body halves of the same individual. Although cut marks were obviously produced during the same butchering process, their morphology can differ substantially, indicating that different stone tools, possibly handled by different butchers, were used in carcass processing (compare fig. S18A). Cut marks occur repeatedly in the same positions on the bones of different animals, suggesting that the dismemberment of these animals followed a more or less standard procedure. It is also noticeable that not a single carcass element provided evidence for bone cracking, in line with the generally inferred rarity of hollow marrow cavities and free marrow in elephant bones, which are structurally different from those of other ungulates (*32*). All observed bone breaks are of modern origin and result from excavation damage.

In their study of cut marks made in the butchering of recent African elephants, Haynes and Krasinski (*11*) documented two types of carcass utilization that are relevant for the Neumark-Nord case. In satisficing carcass processing, butchering was ceased before all possible returns were taken; butchers were satisfied to take tusks, the largest parts of muscle masses, and skin before abandoning the carcass. Cut marks were not observed on large limb elements. In extended utilization, efforts were made to completely strip meat scraps remaining on the largest limb bones, and cut marks were clearly visible on limb bone diaphyses [(*11*), p.21]. Extended utilization often resulted in cut mark patterns that were repeatedly documented at Neumark, such as “parallel or subparallel fields at right angles or diagonal to a bone’s long axis” [(*11*), p. 10], or cut marks on interior faces of proboscidean ribs, which these authors even see as indicative of maximized carcass utilization.

Butchering of elephants yields large packages of protein-rich meat. Other macronutrient food resources must have been a welcome and necessary supplement to balance the daily dietary needs of Neanderthals (see also text S2). Particularly noteworthy at Neumark-Nord is the focus on fatty tissues, for example, visible in the frequent and meticulous exploitation of the large fatty weight–supporting foot cushions, as indicated by the cut mark distribution data. These foot cushions are a well-known repository of adipose tissue in present-day elephants and, together with the trunk, form a highly prized body part for consumption by recent indigenous elephant hunters (*6*). They are also quite resistant to fast degradation processes and spoiling and are a rich source of nutritionally valuable n-3 long-chain polyunsaturated fatty acids (*33*). In an experimental butchering of an Indian elephant, it took a researcher about 3 hours to remove the fatty food cushion, which weighed 1.8 kg. That study suggests that removing the foot from the leg makes accessing the fat cushion easier and would have made the fat package easier to transport (*31*). Furthermore, the Neumark-Nord butchering activities were also clearly aimed at obtaining brain matter. Muscle meat was also clearly targeted: The cut mark patterns and the absence of indications for bone breakage strongly suggest that muscle masses were not removed to facilitate the breakage of bones but that, instead, dismemberment and defleshing testify to a strong interest in meat. Because of the frequently observed cut marks on the diaphyses of long bones, we can assume that this meat was fresh and that we are not witnessing long-term exploitation of ripening animals, which would allow for bulk removal of muscle masses, resulting in a lack of cut marks on longbone diaphyses (*11*). Thus, we infer that the Neumark-Nord evidence is indicative of rather swift and extended butchering of fresh carcasses, supported by the lack of indication that carnivores had first access to the carcasses.

## DISCUSSION

This study of the Neumark-Nord 1 *P. antiquus* bone complexes, with an MNI of 57, demonstrates that Neanderthals had primary access to fresh carcasses and butchered these in similar ways, involving extensive processing, during the first half of the Last Interglacial. This exceptionally rich assemblage represents a specific part of the straight-tusked elephant population. The peculiar age structure of the assemblage identified by Palombo and colleagues (*3*, *24*) indicates that hominins were not focusing on animals that died natural deaths around the lake but instead targeting very specific age classes. This becomes even more apparent when considering elephants whose incomplete preservation inhibits a precise age determination but whose bone size is inconsistent with young age (*3*, *24*). As only for 23 of the 57 individuals in the assemblage was determination of sex possible, the presence of female individuals in the assemblage might be underestimated [but see (*3*, *24*)]. However, the unusually high number of adult and old male individuals observed by Palombo and colleagues (*3*, *24*) might be related to the strong sexual dimorphism of *P. antiquus*, with males substantially taller, with a body mass more than twice that of females, and growing in stature longer than females until old age. With extant elephants, older male adults usually keep to themselves except when individual animals are in season, and assuming similar behavior, at Neumark-Nord, the largest “calorie packages” would have roamed the lake landscape in relative isolation, without the protection of a herd, and would have been easier to approach closely, compared to females in mixed herd groups. Hunting of females may have called for different hunting tactics compared to hunts of males and could have entailed larger hunting parties, given the need to separate individual prey animals from the protective herd. Focusing on adult and old males would give hunters the highest returns for a significantly lower risk.

On the basis of Churchill’s (*34*) ethnographic data, hunting of these single giant individuals may have required little technological sophistication, with hunting strategies mostly aimed at limiting the mobility of prey, e.g., by digging pits or driving them into mud traps, and killing them with wooden thrusting spears (*35*). These weapons are well documented in the archaeological record at Schöningen (*36*) and Lehringen (*8*), i.e., from the second half of the Middle Pleistocene onward. Indirect evidence for the use of spears comes from Neumark-Nord 1 itself in the form of hunting lesions on fallow deer bones uncovered at the site (*18*).

All the data presented above indicate that hunting caused the accumulation of this assemblage. It constitutes the earliest unambiguous evidence for the systematic targeting and processing of straight-tusked elephants, the largest Pleistocene terrestrial mammals that ever lived. This has important implications for our views of Neanderthal local group sizes, mobility, and cooperation, for several reasons.

First, the processing of an average adult *P. antiquus* male individual such as E9, with an estimated body mass of 10 metric tons, would have taken a considerable amount of time, minimally two times the 100– to 300–person-hour estimates listed in (*6*) for much smaller African elephants. Given the extended butchering with stone tools, which consisted of removing all meat, including scraps from the long bones, these estimates should be seen as minima in the case of Neumark-Nord. The butchering of these large animals, and probably the subsequent preparation and storage of meat and fat, required cooperation between several individuals and staying in one place during this processing time. If, as seems likely, meat and fat were also prepared for storage, this would have further added to the time involved: As an illustration, Lupo and Schmitt (*6*) calculated 745 person-hours for butchery, drying, and smoking of African elephants. Regarding the amount of time a Neanderthal group of foragers would need to skin, strip meat from bones, and dry or smoke the meat from an adult male *P. antiquus*, we suggest, as a very rough estimate, that this could be done in 3 to 5 days, if 25 individuals were involved in the process.

Second, the processing of these animals yielded very large amounts of food no matter how difficult it is to quantify the exact amounts (see text S2). One complicating factor in these calculations is the human need to balance consumption of mammal tissue with nonprotein calories to avoid severe and potentially fatal health problems, such as “rabbit starvation,” documented in hunters forced to subsist on lean meat in the north (*37*, *38*). As detailed in text S2, the extended processing of the approximately 10–metric ton E9 individual, for example, would have yielded more than 2500 daily portions, taking this constraint into account: i.e., minimally 2500 adult Neanderthal rations of 4000 kcal (see text S2). These minimum estimates are important: They either imply a large group of consumers and/or the presence of cultural means to preserve food and to store products over a significant period.

Among contemporary low-density hunter-gatherers [see also (*39*)], the number of people living together in a residential group varies between about 15 and 30 individuals (*40*), with an average group size of 25 (*41*). Neanderthal local group (band) size is usually inferred as being smaller than that (*42*). Assuming the conventional extant hunter-gatherer local group size of 25, the values for calories and daily portions calculated (text S2) would provide food for at least 3 months, provided the existence of cultural ways to store food over such periods.

An anecdotal description of Mbuti hunter-gatherer exploitation of an elephant suggests that these estimates may be on the right scale: “They cut and take as much meat as they like ... More than a half ton of meat was obtained from the elephant, and this was enough for about 40 camp members to have meat feast for a week, even when part of the meat was brought to the village for exchanging with the Bantu cultivators for cassava, plantain and other agricultural food.” [(*43*), p.462]. Note that this is a very small amount compared with our estimates for the meat obtained from the Neumark-Nord elephants, i.e., more than 4metric tons for E9 (see text S2) and even more for the approximately 13–metric ton heavy E10, E23B, E26, and E29A individuals. In this Neumark-Nord context, a larger local group size seems more plausible, both because it would have reduced the time invested in processing by individuals and because of the resulting abundance of food implied by the extended processing. In the ethnographic record, meat bonanzas were sometimes taken as an opportunity for a temporary gathering of people from the larger social network, and we cannot exclude such a possibility for Neumark-Nord either. Using the same values for calories and daily portions calculated in text S2, a group of 100 adult individuals could eat the products of a single elephant carcass for nearly a month, and it would take more than 350 people to consume all that food within a week.

Our study demonstrates that Last Interglacial hominins were systematically and regularly targeting adult *P. antiquus* individuals, the largest herbivores of the entire Pleistocene. Given the ~300 years over which the 52 unit 6.1 individuals may have accumulated, one can infer that such hunts were not everyday events, with approximately one individual exploited per 5 to 6 years at this location, disregarding an unknown number of individuals destroyed unnoticed during mining. Irrespective of how often Neanderthals successfully targeted elephants here, their hunting activities also included a wide array of other animal taxa: These elephant hunts took place against the background of regular hunting and processing of prey animals well recorded elsewhere in the lake landscape (see above), including fallow deer, red deer, bovids, and horses. These other hunting and processing activities were studied in detail at Neumark-Nord 2, level NN2/2B, where heavily cut marked and fragmented faunal remains represent a minimum number of 166 large mammals [horses (MNI, 56), bovids (MNI, 40), and cervids (MNI, 53)], as mentioned above, which were transported to the location for extended processing (*17*).

Integrating the Neumark-Nord 2/2B data with the elephant processing data from Neumark-Nord 1 yields an interesting perspective on the wider record of Neanderthal hunting and butchering activities. Despite the high resolution of the Neumark-Nord 2/2B data, including evidence that Neanderthals were butchering animals there year round (*17*), we cannot quantify the period over which this very rich assemblage accumulated: 1 year, a few decades, or even longer? While this can be constrained by palynological data to a period of maximally 455 years (*19*), this assemblage may constitute the accumulation of a large series of hunting and processing events over this period or of a small number of important events, with many prey animals involved at the same time. We simply cannot tell. However, with the elephants from the contemporaneous Neumark-Nord 1 unit 6.1 find level, we know that we are dealing with discrete events, in which Neanderthals hunted and extensively butchered the largest animal ever documented in the Pleistocene archaeological record. The wider Neumark-Nord record, with its evidence for a year-round presence, a limited carnivore signal, and a broad diet, suggests that Neanderthals in this area were less mobile than commonly inferred.

Hunting of a wide range of prey animals, from small cervids up to and including large and dangerous animals, including bears and rhinoceroses, has been well documented in the archaeological record of Middle and Late Pleistocene Neanderthals for some time now (*44*–*47*), with some evidence suggesting overhunting of some prey species in the Levant (*48*). Our elephant window into Pleistocene subsistence strategies now shows unambiguously that Middle Paleolithic foragers not only hunted a wide array of prey animals, including the largest terrestrial mammals of the Pleistocene: They were also capable of dealing with large amounts of meat and fat in relatively short processing activities, easily and routinely managed by Neanderthals, as demonstrated by the exceptionally large food packages that they produced at Neumark-Nord 1. This opens up a new frame of reference for the interpretation of large accumulations of cut-marked and broken faunal remains, not only at Neumark-Nord 2 but also at other sites, e.g., the horses from the 300-ka-old site of Schöningen (*49*, *50*), the various faunal complexes from the 200-ka-old site Biache-Saint-Vaast (France), which include many cut-marked rhinoceros remains (*46*), or the rich reindeer assemblage from the Last Glacial (early Weichselian) site of Salzgitter-Lebenstedt (*45*). Often interpreted as the results of a single communal hunting event (*40*), the fat and protein products of such a reindeer hunt (MNI, 86) would have been rather similar to that of one 13–metric ton Neumark-Nord elephant. The Neumark-Nord elephant record supports the notion that these accumulations do not necessarily reflect palimpsests of independent small-scale hunting and processing events and instead points to the possibility of larger-scale collective subsistence activities, depending on mechanisms for cooperation (*39*).

Furthermore, a site complex c. 54 km to the southeast of Neumark-Nord suggests that the elephant record presented here may not be an isolated, one-off occurrence. A rich assemblage of *P. antiquus* remains, strongly biased in favor of identifiable and “paleontologically interesting” faunal remains, was retrieved from stone artifact–yielding sands and silts at Taubach (Germany), mostly within a relatively short period during the late 19th century (*44*). There seem to have been remarkable concentrations of elephant remains, with an MNI of 40 in February 1891 increasing to 50 in the autumn of that same year (*44*). Unfortunately, the extant sample is not associated with good records of the find situation, while bone fragments not considered of paleontological value were simply thrown away during fieldwork (*44*). In addition, rhinoceroses are the most common species at this site and are better documented and studied. Cut marks are well represented on a range of skeletal elements. With its strong focus on juveniles (*44*), the age profile of these rhinos differs from that of the elephants at Neumark-Nord 1. However, a point of similarity may be the focus on solitary animals, since (based on modern comparisons) these are likely to have been solitary or small-group dwelling animals, and the juveniles may have been old enough to be apart from mothers at least some of the time (*44*, *51*). Taubach provides an example of another site from the same region at which Neanderthals repeatedly exploited very large mammals over a relatively long time.

Other sites from the wider region with single-elephant carcasses and evidence for Neanderthal activity share some similarities and some differences with the Neumark-Nord 1 pattern. At the famous site of Lehringen, a single *P. antiquus* skeleton was found with a carefully modified yew wood lance among the bones (*8*). Another nearly complete skeleton was recovered at Gröbern, 55 km northeast of Neumark-Nord (*9*). At both sites, about two dozen flint artifacts were recovered, none of them consisting of formal tools. Similarities with the Neumark-Nord 1 elephants include the adult status of the elephant (both were more than 35 years old). At Neumark-Nord, the very strong record of extended utilization of fresh elephant carcasses only contains a very weak lithic signal of human presence at these butchering locales, consisting of a few informal flakes only. Therefore, a weak lithic signal does not necessarily imply limited processing of megafauna. However, while the apparent lack of cut marks in a similar depositional context might be due to preservation, it could also suggest variation in Neanderthal exploitation strategies, at the Lehringen and Gröbern sites, for instance, involving either less intensive processing or later access.

While the quality and quantity of the Neumark-Nord evidence are thus far unique in the archaeological record of the Pleistocene, as a result of the unique preservation conditions in this lake landscape, these exceptional data may afford us an unparalleled insight into rather frequent Neanderthal behavior during the Last Interglacial in the middle latitudes of Europe. Alternatively, specific characteristics of the lake area may have facilitated a specific type of behavior (including regular hunting of straight-tusked elephants) that was not more widely distributed during the Last Interglacial. We have no comparative material from other sites of similar quality and quantity to address this question straightforwardly. At first glance, the primary context associations of flint artifacts and skeletal remains at Lehringen and Gröbern do seem to fit the Neumark-Nord pattern described here. This may suggest that exploitation of elephants was a wider-spread phenomenon 125,000 years ago and that Neanderthal behavior at Neumark-Nord was not isolated or unusual nor was the impact that they made on their local environment, as the neighboring site of Rabutz shows strong similarities in that domain (*22*). A remaining question is whether this behavior can be extrapolated across the whole region or whether the nature of these activities and occupation pattern is better characterized by hot and cool spots.

Last, the Neumark-Nord record of exploitation of a wide array of prey animals, including the largest Pleistocene terrestrial mammals, combines with a clear footprint of anthropogenic vegetation openness in the local environment more than 2000 years in the first part of the Last Interglacial (*22*). This suggests that Pleistocene foragers were less mobile and transient and had more impact on their ecosystems than often hypothesized. Moreover, the elephant hunting and processing data presented here also suggest that in this landscape, Last Interglacial Neanderthals operated in substantially larger groups than the usually inferred maximum of 25 people in band units. Instead, they point to the existence of larger-scale collective subsistence activities already during the Middle Paleolithic and possibly much earlier and, thus, open up a new window into the character and the time depth of human larger-scale cooperation.

## MATERIALS AND METHODS

### Assemblage composition

The sample analyzed from Neumark-Nord 1 comprised 3122 bones and teeth of *P. antiquus*. Bones and teeth were retrieved from 44 bone complexes; their descriptions by Mania (*23*) served as a point of departure for this study. According to Mania, these bone complexes are spatially restricted find spots characterized by more or less complete carcasses of one or more individuals. Mania either documented them in situ or noted which ones were more or less disturbed by the excavator. Eight of these could not be recovered in the field. For four other complexes, only the recovery of some skull/molar fragments was possible. Nine further complexes from the main find layer unit 6.1 are exclusively represented by one (or more) tusk(s). The remaining bone complexes from units 4, 6, 6.1, and 7 yielded cranial and postcranial material. Further stratified material (C1-C2) and unstratified material (C3) (see table S1) could not be assigned to any of the bone complexes. This material increases the MNI by 5.

All recovered bone complexes were examined for bone surface modifications (Table 1). All finds are kept at the Landesamt für Archäologie Sachsen-Anhalt in Halle (Germany).

### Methods

#### Taxonomic, age, and sex determination

The *P. antiquus* sample from Neumark-Nord 1 was paleontologically analyzed and described in (*3*, *24*). These studies yielded data on the number of individuals for the different bone complexes, age at death, sex, and certain parameters of life appearance, focusing thereby primarily—but not only—on skulls, mandibles, and molars from unit 6.1. For the reconstruction of the life appearance of *P. antiquus*, a later study by Larramendi *et al.* (*1*) additionally included individuals from unit 6 (E8, E9, E10B, and E34A). We used these published data in our sample description, as well as for age and sex determination and for body mass estimates of the individuals in our sample.

#### Number of identified specimen per taxon and minimum number of elements

For the taxonomic determination of bones and bone fragments in our study, we used (*52*–*55*) and complete bones from *P. antiquus* deriving from the Neumark-Nord 1 sample itself. Minimum numbers of elements (MNEs) were calculated from the number of identified specimen per taxon (NISP) using (*27*).

#### Minimum number of individuals

For each of the spatially separated bone complexes, MNIs were calculated following (*56*). In cases where several individuals were represented, we took advantage of information on ontogeny and sex provided in (*24*) for bone complexes from unit 6.1 and in (*1*) for some bone complexes (see above) from unit 6.

#### Bone surface modifications

Bone surface modifications were studied using hand-held lenses with a magnification of up to ×10, a Dino-Lite PRO digital microscope with a magnification up to ×200, and a Leica reflected-light microscope with a magnification of up to ×32. For each bone or bone fragment, the location of the observed traces on the bone was photographed with a Nikon camera, while close-ups of the bone surface modifications were produced with the Dino-Lite PRO equipment. Traces caused by biotic and abiotic agents were identified using the taphonomic collection of the Archaeological Research Center and Museum for Human Behavioral Evolution, MONREPOS, and diagnostic criteria published in (*57*).

## Supplementary Materials

### This PDF file includes:

Supplementary Text 1 and 2 Figs. S1 to S18.2 Tables S1 to S15 References

## References

[1] A. Larramendi, M. R. Palombo, F. Marano, Reconstructing the life appearance of a Pleistocene giant: Size, shape, sexual dimorphism and ontogeny of *Palaeoloxodon antiquus* (Proboscidea: Elephantidae) from Neumark-Nord 1 (Germany). Boll. Della Soc. Paleontol. Ital. 56, 299–317 (2017).

[2] A. J. Stuart, The extinction of woolly mammoth (*Mammuthus primigenius*) and straight-tusked elephant (*Palaeoloxodon antiquus*) in Europe. Quat. Int. 126–128, 171–177 (2005).

[3] M. R. Palombo, E. Albayrak, F. Marano, The straight-tusked elephants from Neumark-Nord. A glance into a lost world, in *Elefantenreich: eine Fossilwelt in Europa ; Begleitband zur Sonderausstellung im Landesmuseum für Vorgeschichte Halle 26.03.-03.10.2010*, H. Meller, Ed. (Landesamt für Denkmalpflege und Archäologie Sachsen-Anhalt, Landesmuseum für Vorgeschichte, 2010), pp. 218–251.

[4] E. Santucci, F. Marano, E. Cerilli, I. Fiore, C. Lemorini, M. R. Palombo, A. P. Anzidei, G. M. Bulgarelli, *Palaeoloxodon* exploitation at the Middle Pleistocene site of La Polledrara di Cecanibbio (Rome, Italy). Quat. Int. 406, 169–182 (2016).

[5] G. Haynes, Late Quaternary Proboscidean sites in Africa and Eurasia with possible or probable evidence for hominin involvement. Quat. 5, 18 (2022).

[6] K. D. Lupo, D. N. Schmitt, When bigger is not better: The economics of hunting megafauna and its implications for Plio-Pleistocene hunter-gatherers. J. Anthropol. Archaeol. 44, 185–197 (2016).

[7] W. Soergel, *Die Jagd der Vorzeit* (Gustav Fischer, 1922).

[8] H. Thieme, S. Veil, Neue Untersuchungen zum eemzeitlichen Elefanten-Jagdplatz Lehringen, Ldkr. Verden. Kunde 36, 11–58 (1985).

[9] D. Mania, M. Thomae, T. Litt, T. Weber, Eds., *Neumark-Gröbern: Beiträge zur Jagd des mittelpaläolithischen Menschen* (Deutscher Verlag der Wissenschaften, 1990), *Veröffentlichungen des Landesmuseums für Vorgeschichte in Halle.*

[10] G. E. Konidaris, V. Tourloukis, Proboscidea-Homo interactions in open-air localities during the Early and Middle Pleistocene of western Eurasia: A palaeontological and archaeolocigal perspective, in *Human-Elephant Interactions: From Past to Present*, G. E. Konidaris, R. Barkai, V. Tourloukis, K. Harvati, Eds. (Tuebingen paleoanthropology book series - contributions in paleoanthropology, Tübingen Univ. Press, 2021), pp. 67–104;https://publikationen.uni-tuebingen.de/xmlui/handle/10900/114224.

[11] G. Haynes, K. Krasinski, Butchering marks on bones of *Loxodonta africana* (African savanna elephant): Implications for interpreting marks on fossil proboscidean bones. J. Archaeol. Sci. Rep. 37, 102957 (2021).

[12] G. Haynes, J. Klimowicz, Recent elephant-carcass utilization as a basis for interpreting mammoth exploitation. Quat. Int. 359–360, 19–37 (2015).

[13] M. R. Palombo, E. Cerilli, Human-Elephant interactions during the Lower Palaeolithic: Scrutinizing the role of environmental factors, in *Human-Elephant Interactions: From Past to Present*, G. Konidaris, R. Barkai, V. Tourloukis, K. Harvati, Eds. (Tuebingen paleoanthropology book series - contributions in paleoanthropology, Tübingen Univ. Press, 2021), pp. 105–143; 10.15496/publikation-55604.

[14] H. Bocherens, D. G. Drucker, M. Germonpré, M. Lázničková-Galetová, Y. I. Naito, C. Wissing, J. Brůžek, M. Oliva, Reconstruction of the Gravettian food-web at Předmostí I using multi-isotopic tracking (^13^C, ^15^N, ^34^S) of bone collagen. Quat. Int. 359–360, 211–228 (2015).

[15] J. Z. Metcalfe, Proboscidean isotopic compositions provide insight into ancient humans and their environments. Quat. Int. 443, 147–159 (2017).

[16] S. Gaudzinski-Windheuser, W. Roebroeks, Eds., *Multidisciplinary Studies of the Middle Palaeolithic Record from Neumark-Nord (Germany), Vol. I* (LDA-LSA, Halle (Saale), 2014), *Veröffentlichungen des Landesamtes für Denkmalpflege und Archäologie Sachsen-Anhalt - Landesmuseum für Vorgeschichte.*

[17] L. Kindler, G. M. Smith, A. Garcia-Moreno, S. Gaudzinski-Windheuser, E. Pop, W. Roebroeks, The last interglacial (Eemian) lakeland of Neumark-Nord (Saxony-Anhalt, Germany). Sequencing Neanderthal occupations, assessing subsistence opportunities and prey selection based on estimations of ungulate carrying capacities, biomass production and energy values, in *Human Behavioural Adaptations to Interglacial Lakeshore Environments*, A. Garcia-Moreno, J. M. Hutson, G. M. Smith, L. Kindler, E. Turner, A. Villaluenga, S. Gaudzinski-Windheuser, Eds. (RGZM-Tagungen, Propylaeum, 2020), pp. 67–104;https://books.ub.uni-heidelberg.de/index.php/propylaeum/catalog/book/647.

[18] S. Gaudzinski-Windheuser, E. S. Noack, E. Pop, C. Herbst, J. Pfleging, J. Buchli, A. Jacob, F. Enzmann, L. Kindler, R. Iovita, M. Street, W. Roebroeks, Evidence for close-range hunting by last interglacial Neanderthals. Nat. Ecol. Evol. 2, 1087–1092 (2018). 29942012 10.1038/s41559-018-0596-1

[19] M. J. Sier, W. Roebroeks, C. C. Bakels, M. J. Dekkers, E. Brühl, D. De Loecker, S. Gaudzinski-Windheuser, N. Hesse, A. Jagich, L. Kindler, W. J. Kuijper, T. Laurat, H. J. Mücher, K. E. H. Penkman, D. Richter, D. J. J. van Hinsbergen, Direct terrestrial–marine correlation demonstrates surprisingly late onset of the last interglacial in central Europe. Quat. Res. 75, 213–218 (2011). 26523075 10.1016/j.yqres.2010.11.003 PMC4600610

[20] D. Mania, H. Meller, Eds., *Neumark-Nord: ein interglaziales Ökosystem des mittelpaläolithischen Menschen* (LDA-LSA, Halle (Saale), 2010), *Veröffentlichungen des Landesamtes für Denkmalpflege und Archäologie Sachsen-Anhalt - Landesmuseum für Vorgeschichte*.

[21] E. Pop, W. Kuijper, E. van Hees, G. Smith, A. García-Moreno, L. Kindler, S. Gaudzinski-Windheuser, W. Roebroeks, Fires at Neumark-Nord 2, Germany: An analysis of fire proxies from a Last Interglacial Middle Palaeolithic basin site. J. Field Archaeol. 41, 603–617 (2016).

[22] W. Roebroeks, K. MacDonald, F. Scherjon, C. Bakels, L. Kindler, A. Nikulina, E. Pop, S. Gaudzinski-Windheuser, Landscape modification by Last Interglacial Neanderthals. Sci. Adv. 7, eabj5567 (2021). 34910514 10.1126/sciadv.abj5567 PMC8673775

[23] D. Mania, Der Fossilbericht von den Waldelefanten im Seebecken von Neumark-Nord, in *Elefantenreich: eine Fossilwelt in Europa ; Begleitband zur Sonderausstellung im Landesmuseum für Vorgeschichte Halle 26.03.-03.10.2010*, H. Meller, Ed. (Landesamt für Denkmalpflege und Archäologie Sachsen-Anhalt, Landesmuseum für Vorgeschichte, 2010), pp. 201–217.

[24] F. Marano, M. R. Palombo, Population structure in straight-tusked elephants: A case study from Neumark Nord 1 (late Middle Pleistocene?, Sachsen-Anhalt, Germany). Boll. Della Soc. Paleontol. Ital. 52, 207–218 (2013).

[25] H. Müller, Pollenanalytische Untersuchungen und Jahresschichtenzählungen an der eem-zeitlichen Kieselgur von Bispingen/Luhe. Geol. Jahrb. A21, 149–169 (1974).

[26] M. Meyer, E. Palkopoulou, S. Baleka, M. Stiller, K. E. H. Penkman, K. W. Alt, Y. Ishida, D. Mania, S. Mallick, T. Meijer, H. Meller, S. Nagel, B. Nickel, S. Ostritz, N. Rohland, K. Schauer, T. Schüler, A. L. Roca, D. Reich, B. Shapiro, M. Hofreiter, Palaeogenomes of Eurasian straight-tusked elephants challenge the current view of elephant evolution. eLife 6, e25413 (2017). 28585920 10.7554/eLife.25413 PMC5461109

[27] R. L. Lyman, *Vertebrate Taphonomy* (Cambridge Manuals in Archaeology, Cambridge Univ. Press, 1994);http://site.ebrary.com/id/10897773.

[28] G. Haynes, P. Wojtal, Weathering stages of proboscidean bones: Relevance for zooarchaeological analysis. J. Archaeol. Method Theory 10.1007/s10816-022-09569-3, (2022).

[29] H. Meller, Ed., *Elefantenreich: eine Fossilwelt in Europa ; Begleitband zur Sonderausstellung im Landesmuseum für Vorgeschichte Halle 26.03.-03.10.2010* (Landesamt für Denkmalpflege und Archäologie Sachsen-Anhalt, Landesmuseum für Vorgeschichte, 2010).

[30] G. E. Weissengruber, G. F. Egger, J. R. Hutchinson, H. B. Groenewald, L. Elsässer, D. Famini, G. Forstenpointner, The structure of the cushions in the feet of African elephants (*Loxodonta africana*). J. Anat. 209, 781–792 (2006). 17118065 10.1111/j.1469-7580.2006.00648.x PMC2048995

[31] B. M. Starkovich, P. Cuthbertson, K. Kitagawa, N. Thompson, G. E. Konidaris, V. Rots, S. C. Münzel, D. Giusti, V. C. Schmid, A. Blanco-Lapaz, C. Lepers, V. Tourloukis, Minimal tools, maximum meat: A pilot experiment to butcher an elephant foot and make elephant bone tools using lower paleolithic stone tool technology. Ethnoarchaeology 12, 118–147 (2020).

[32] G. Boschian, D. Caramella, D. Saccà, R. Barkai, Are there marrow cavities in Pleistocene elephant limb bones, and was marrow available to early humans? New CT scan results from the site of Castel di Guido (Italy). Quat. Sci. Rev. 215, 86–97 (2019).

[33] J. L. Guil-Guerrero, A. Tikhonov, R. P. Ramos-Bueno, S. Grigoriev, A. Protopopov, G. Savvinov, M. J. González-Fernández, Mammoth resources for hominins: From omega-3 fatty acids to cultural objects. J. Quat. Sci. 33, 455–463 (2018).

[34] S. E. Churchill, Weapon technology, prey size selection, and hunting methods in modern hunter-gatherers: Implications for hunting in the Palaeolithic and Mesolithic. Archeol. Pap. Am. Anthropol. Assoc. 4, 11–24 (1993).

[35] A. Milks, A review of ethnographic use of wooden spears and implications for pleistocene hominin hunting. Open Quat. 6, 12 (2020).

[36] H. Thieme, Lower Palaeolithic hunting spears from Germany. Nature 385, 807–810 (1997). 9039910 10.1038/385807a0

[37] J. D. Speth, K. A. Spielmann, Energy source, protein metabolism, and hunter-gatherer subsistence strategies. J. Anthropol. Archaeol. 2, 1–31 (1983).

[38] J. D. Speth, *The Paleoanthropology and Archaeology of Big-Game Hunting* (Interdisciplinary Contributions to Archaeology, Springer, 2010; 10.1007/978-1-4419-6733-6.

[39] R. Boyd, P. J. Richerson, Large-scale cooperation in small-scale foraging societies. Evol. Anthropol. 31, 175–198 (2022). 35485603 10.1002/evan.21944

[40] D. W. Bird, R. B. Bird, B. F. Codding, D. W. Zeanah, Variability in the organization and size of hunter-gatherer groups: Foragers do not live in small-scale societies. J. Hum. Evol. 131, 96–108 (2019). 31182209 10.1016/j.jhevol.2019.03.005

[41] R. L. Kelly, *The Lifeways of Hunter-Gatherers: The Foraging Spectrum* (Cambridge Univ. Press, ed. 2, 2013).

[42] S. E. Churchill, *Thin on the Ground: Neandertal Biology, Archeology, and Ecology* (John Wiley & Sons Inc., 2014); 10.1002/9781118590836.

[43] M. Ichikawa, Elephant hunting by the Mbuti hunter-gatherers in the Eastern Congo Basin, in *Human-Elephant Interactions: From Past to Present*, G. Konidaris, R. Barkai, V. Tourloukis, K. Harvati, Eds. (Tuebingen paleoanthropology book series - contributions in paleoanthropology, Tübingen Univ. Press, 2021), pp. 455–467; 10.15496/publikation-55604.

[44] B. Bratlund, Taubach revisited. Jahrb. Röm. Ger. Zentralmuseums. 46, 61–174 (1999).

[45] S. Gaudzinski, W. Roebroeks, Adults only. Reindeer hunting at the Middle Palaeolithic site Salzgitter Lebenstedt, Northern Germany. J. Hum. Evol. 38, 497–521 (2000). 10715194 10.1006/jhev.1999.0359

[46] P. Auguste, Chasse et charognage au Paléolithique moyen: L’apport du gisement de Biache-Saint-Vaast (Pas-de-Calais). Bull. Société Préhistorique Fr. 92, 155–168 (1995).

[47] L. Kindler, *Die Rolle von Raubtieren bei der Einnischung und Subsistenz jungpleistozäner Neandertaler: Archäozoologie und Taphonomie der mittelpaläolithischen Fauna aus der Balver Höhle, Westfalen* (Verl. des Römisch-Germanischen Zentralmuseums, 2012).

[48] J. Speth, J. Clark, Hunting and overhunting in the Levantine Late Middle Palaeolithic. Farming 2006, 1–42 (2006).

[49] J. M. Hutson, A. Villaluenga, A. García-Moreno, E. Turner, S. Gaudzinski-Windheuser, A zooarchaeological and taphonomical perspective of hominin behaviour from the Schöningen 13II-4 “Spear Horizon”, in *Human Behavioural Adaptations to Interglacial Lakeshore Environments* (RGZM-Tagungen, Propylaeum, 2020), pp. 43–66;https://books.ub.uni-heidelberg.de/index.php/propylaeum/catalog/book/647.

[50] A. García-Moreno, J. M. Hutson, A. Villaluenga, E. Turner, S. Gaudzinski-Windheuser, A detailed analysis of the spatial distribution of Schöningen 13II-4 ‘Spear Horizon’ faunal remains. J. Hum. Evol. 152, 102947 (2021). 33529840 10.1016/j.jhevol.2020.102947

[51] G. L. Dusseldorp, A View to a kill: Investigating Middle Palaeolithic Subsistence Using an Optimal Foraging Perspective (Sidestone Press, 2009).

[52] M. M. Smuts, A. J. Bezuidenhout, Osteology of the thoracic limb of the African elephant (Loxodonta africana). Onderstepoort J. Vet. Res. 60, 1–14 (1993). 8332313

[53] A. J. Bezuidenhout, C. D. Seegers, The osteology of the African elephant (Loxodonta africana): Vertebral column, ribs and sternum. Onderstepoort J. Vet. Res. 63, 131–147 (1996). 8856763

[54] N. J. van der Merwe, A. J. Bezuidenhout, C. D. Seegers, The skull and mandible of the African elephant (*Loxodonta africana*). Onderstepoort J. Vet. Res. 62, 245–260 (1995). 8668323

[55] G. J. Stanek, thesis, Veterinärmedizinische Universität Wien, Wien (2012).

[56] R. G. Klein, K. Cruz-Uribe, *The Analysis of Animal Bones From Archeological Sites* (Prehistoric archeology and ecology, University of Chicago Press, 1984).

[57] Y. Fernández-Jalvo, P. Andrews, *Atlas of Taphonomic Identifications 1001+ Images of Fossil and Recent Mammal Bone Modification* (Vertebrate Paleobiology and Paleoanthropology Series, Springer, 2016);http://springerlink.com/content/978-94-017-7432-1.

[58] B. Menke, R. Tynni, Das Eeminterglazial und das Weichselfrühglazial von Rederstall/Dithmarschen und ihre Bedeutung für die mitteleuropäische Jungpleistozän-Gliederung. Geol. Jahrb. A76, 3–120 (1984).

[59] R. Grube, M. R. Palombo, P. Iacumin, A. Di Matteo, What did the fossil elephants from Neumark-Nord eat?, in *Elefantenreich: eine Fossilwelt in Europa; Begleitband zur Sonderausstellung im Landesmuseum für Vorgeschichte Halle 26.03.-03.10.2010*, H. Meller, Ed. (Landesamt für Denkmalpflege und Archäologie Sachsen-Anhalt, Landesmuseum für Vorgeschichte, 2010), pp. 252–274.

[60] J. Koller, U. Baumer, Der organische Belag auf der Silexklinge aus Neumark-Nord. Gerbungsmaterial oder Schäftungskit?, in *Elefantenreich: eine Fossilwelt in Europa ; Begleitband zur Sonderausstellung im Landesmuseum für Vorgeschichte Halle 26.03.-03.10.2010*, H. Meller, Ed. (Landesamt für Denkmalpflege und Archäologie Sachsen-Anhalt, Landesmuseum für Vorgeschichte, 2010), pp. 553–563.

[61] D. A. Byers, A. Ugan, Should we expect large game specialization in the late Pleistocene? An optimal foraging perspective on early Paleoindian prey choice. J. Archaeol. Sci. 32, 1624–1640 (2005).

[62] G. C. Frison, L. C. Todd, *The Colby Mammoth Site: Taphonomy and Archaeology of a Clovis Kill in Northern Wyoming* (University of New Mexico Press, ed. 1, 1986).

[63] S. Bilsborough, N. Mann, A review of issues of dietary protein intake in humans. Int. J. Sport Nutr. Exerc. Metab. 16, 129–152 (2006). 16779921 10.1123/ijsnem.16.2.129

[64] L. Cordain, J. B. Miller, S. B. Eaton, N. Mann, S. H. Holt, J. D. Speth, Plant-animal subsistence ratios and macronutrient energy estimations in worldwide hunter-gatherer diets. Am. J. Clin. Nutr. 71, 682–692 (2000). 10702160 10.1093/ajcn/71.3.682

[65] R. S. Kuipers, M. F. Luxwolda, D. A. Janneke Dijck-Brouwer, S. B. Eaton, M. A. Crawford, L. Cordain, F. A. J. Muskiet, Estimated macronutrient and fatty acid intakes from an East African Paleolithic diet. Br. J. Nutr. 104, 1666–1687 (2010). 20860883 10.1017/S0007114510002679

[66] D. E. Chusyd, J. L. Brown, C. Hambly, M. S. Johnson, K. Morfeld, A. Patki, J. R. Speakman, D. B. Allison, T. R. Nagy, Adiposity and reproductive cycling status in zoo african elephants. Obesity 26, 103–110 (2018). 29265776 10.1002/oby.22046 PMC5744898

[67] D. E. Chusyd, T. R. Nagy, L. Golzarri-Arroyo, S. L. Dickinson, J. R. Speakman, C. Hambly, M. S. Johnson, D. B. Allison, J. L. Brown, Adiposity, reproductive and metabolic health, and activity levels in zoo Asian elephant (*Elephas maximus* ). J. Exp. Biol. 224, jeb219543 (2021). 33500325 10.1242/jeb.219543 PMC7847275

[68] D. C. Salazar-García, R. C. Power, A. Sanchis Serra, V. Villaverde, M. J. Walker, A. G. Henry, Neanderthal diets in central and southeastern Mediterranean Iberia. Quat. Int. 318, 3–18 (2013).

[69] R. C. Power, D. C. Salazar-García, M. Rubini, A. Darlas, K. Harvati, M. Walker, J.-J. Hublin, A. G. Henry, Dental calculus indicates widespread plant use within the stable Neanderthal dietary niche. J. Hum. Evol. 119, 27–41 (2018). 29685752 10.1016/j.jhevol.2018.02.009

[70] J. A. Fellows Yates, I. M. Velsko, F. Aron, C. Posth, C. A. Hofman, R. M. Austin, C. E. Parker, A. E. Mann, K. Nägele, K. W. Arthur, J. W. Arthur, C. C. Bauer, I. Crevecoeur, C. Cupillard, M. C. Curtis, L. Dalén, M. Díaz-Zorita Bonilla, J. C. Díez Fernández-Lomana, D. G. Drucker, E. Escribano Escrivá, M. Francken, V. E. Gibbon, M. R. González Morales, A. Grande Mateu, K. Harvati, A. G. Henry, L. Humphrey, M. Menéndez, D. Mihailović, M. Peresani, S. Rodríguez Moroder, M. Roksandic, H. Rougier, S. Sázelová, J. T. Stock, L. G. Straus, J. Svoboda, B. Teßmann, M. J. Walker, R. C. Power, C. M. Lewis, K. Sankaranarayanan, K. Guschanski, R. W. Wrangham, F. E. Dewhirst, D. C. Salazar-García, J. Krause, A. Herbig, C. Warinner, The evolution and changing ecology of the African hominid oral microbiome. Proc. Natl. Acad. Sci. U.S.A. 118, e2021655118 (2021). 33972424 10.1073/pnas.2021655118 PMC8157933

[71] A. G. Henry, A. S. Brooks, D. R. Piperno, Plant foods and the dietary ecology of Neanderthals and early modern humans. J. Hum. Evol. 69, 44–54 (2014). 24612646 10.1016/j.jhevol.2013.12.014

[72] W. J. Kuijper, Investigation of inorganic, botanical, and zoological remains of an exposure of Last Interglacial (Eemian) sediments at Neumark-Nord 2 (Germany), in *Multidisciplinary Studies of the Middle Palaeolithic Record from Neumark-Nord (Germany), Vol. I*, S. Gaudzinski-Windheuser, W. Roebroeks, Eds. (Veröffentlichungen des Landesamtes für Denkmalpflege und Archäologie Sachsen-Anhalt - Landesmuseum für Vorgeschichte, LDA-LSA, 2014), pp. 79–97.

[73] E. Pop, C. Bakels, Semi-open environmental conditions during phases of hominin occupation at the Eemian Interglacial basin site Neumark-Nord 2 and its wider environment. Quat. Sci. Rev. 117, 72–81 (2015).

