# Supplementary material for: Hunting and processing of straight-tusked elephants 125.000 years ago: Implications for Neanderthal behavior
Source: Sci Adv. 2023 Feb 1;9(5):eadd8186. doi: 10.1126/sciadv.add8186 (PMC9891704; doi:10.1126/sciadv.add8186)
Supplement: Supplementary file 1 — Supplementary Text 1 and 2 Figs. S1 to S18.2 Tables S1 to S15 References [file sciadv.add8186_sm.pdf]

Science Advances
AAAS

Supplementary Materials for

Hunting and processing of straight-tusked elephants 125.000 years ago: Implications for Neanderthal behavior

Sabine Gaudzinski-Windheuser *et al.*

Corresponding author: Sabine Gaudzinski-Windheuser, sabine.gaudzinski@leiza.de

Sci. Adv. **9**, eadd8186 (2023)
DOI: 10.1126/sciadv.add8186

This PDF file includes:

Supplementary Text 1 and 2
Figs. S1 to S18
Tables S1 to S15
References

Supplementary Text 1: Description of five representative bone complexes from Neumark-Nord 1

To illustrate the character of the find situation of the elephant remains from Neumark-Nord 1, their varying ways of documentation, as well as the distribution of the identified bone surface modifications, we here give a short description of 5 bone complexes, from the main *Palaeoloxodon antiquus* yielding sedimentary units. For the lower (unit 4) and upper (unit 7) units, we present the only bone complexes that could be salvaged from these sediments, E6 and E30 respectively, whereas for the find rich littoral zone unit 6.1 we focus on the virtually complete and most informative skeletons which have yielded an excellent bone surface modification record, providing detailed insight into carcass processing activities (Figure S1).

The following description summarizes the basic information about the bone complexes published by Mania (*23*), supplemented by information from detailed paleontological studies (*1, 3, 24*), and finally lists the skeletal elements examined for bone surface modifications per bone complex. We illustrate some of these bone surface modifications, and give a short description of their distribution as well as a tentative reconstruction of the carcass exploitation sequences.

Unit 4 (PAZ III of the Eemian Interglacial)

Bone complex E6

The sequence of bone complexes starts in the middle part of the lower varved fine detrital muds of unit 4, where *Quercus* appears in the pollen diagrams, i.e. PAZ III (*16*) of the Eemian Interglacial. Only one bone complex, discovered west of the center of the basin, was retrieved from this unit. Three further bone complexes were discovered in the upper part of the varved fine detrital mud (unit 4), but in all cases, no bones could be recovered due to ongoing mining activities. Complex E6 hence constitutes the only excavated skeletal *P. antiquus* evidence from this unit (*23*). E6 represents the remains of a more or less complete elephant carcass, spread over an area of 2x3 meters. Mania estimates that half of the carcass fell victim to the bucket excavator and according to him, virtually all skeletal elements were upon initial discovery still preserved at the location (*23*).

E6 consists of 38 skeletal elements taxonomically identified as *P. antiquus* (Figures S2-S3). An MNE of 19 was calculated from the NISP. A few elements of the zonoskeleton, parts of the left front leg, a few elements of the right front leg, pelvis, and a few elements of primarily right but also left hindleg survived (Table S4).

The following skeletal elements or fragments thereof were identified and studied for bone surface modifications: vertebrae fragments, rib fragments, *Pelvis* sin./dex., *Scapula* sin., *Humerus* indet., *Ulna* sin., *Os carpi intermedium sin*., *Os carpi accessorium* sin., *Os carpale I* sin., *Os carpale II* dex., *Os metacarpale I* dex., *Os metacarpale III* dex., *Femur* dex., *Patella* sin., *Tibia* dex. and *Os metatarsale III* sin.

Five bones displayed cut marks. Tables S4 and S5 list the observed cut marks and assign them to various activities during the butchering process. The cut marks testify to the disarticulation of the left front leg, the separation of the left hind leg from the pelvis, the separation of the left autopodium from zeugopodium, and the complete disarticulation of left and right metacarpals. Traces of carnivore modification were not observed on the remains.

Unit 6 (PAZ IV of the Eemian Interglacial)

Bone complex E9

Bone complex E9 contains the remains of a particularly large male, 47 years of age at death (*1*, *23*). Though the individual was still not fully grown, body mass for this individual was calculated to 9,8 tonnes, with a shoulder height of about 3,63 meters. The individual has a hole in the front of its skull, that does not extend into the pneumatic bone tissue, the reason why the individual survived the injury, also apparent from the ossification of its wound edges (*3*).

The remains of this individual were excavated from the central part of the lake basin, next to the shore. Mania (*23*) provides a detailed report of the find-situation. After the initial uncovering of the bones, the wider spatial extension of the complex was investigated, so that it was possible to record the complete spatial distribution of bone complex E9. Only part of the carcass was still in anatomical position. The *Os sacrum*, still attached to the complete *Pelvis,* was facing downwards and *Radius* with *Ulna* still articulated. The right *Femur*, *Tibia* and *Fibula* were stacked inside the *Pelvis,* while Cranium with mandible and attached cervical spine lay on top. Next to it, the left *Femur*, *Radius* and *Ulna* in anatomical position were located, as well as the left *Humerus,* facing in an anatomically incorrect direction. Parts of the rump as well as the two tusks were missing (Figure S5). Immediately next to the bones Mania recorded a large flint artifact (Figure S6). The E9 individual was used for paleontological reconstructions of the life appearance of *P. antiquus* from Neumark-Nord 1 (*1*).

The well-preserved skull of this bull as well as its mandible have been very heavily restored for exhibition purposes, to such a degree that much of the original bone surface on these pieces is no longer visible. Apart from the skull and the attached cervical spine, *Os sacrum* with articulating vertebrae and *Pelvis,* most of the torso is missing.

Table S6 compiles the qualitative and quantitative composition of bone complex E9 with 74 NISP representing 62 MNE. The following skeletal elements were studied for bone surface modifications: skull, mandible, complete cervical spine up to the 5th cervical vertebra, thoracic vertebrae, lumbar vertebrae, sacrum, caudal vertebra 1, caudal vertebra 2, *Scapula* sin./dex., *Humerus* sin./dex., *Radius/Ulna* sin./dex., *Femur* sin./dex., *Patella* sin., *Tibia* dex., *Fibula* dex., *Os carpi radiale* dex., *Os carpi intermedium* sin./ dex., *Os carpi ulnare* sin., *Os carpi acessorium* sin., *Os carpale I* sin./dex., *Os carpale II* dex., *Os carpale III* sin./dex., *Os carpale IV* dex., *Os metacarpale I-IV* sin./dex., T*alus* dex., *Calcaneus* sin., *Os tarsale III* dex., 3 *Phalanges proximales* sin., *Phalanges* indet. Of these, an MNE of 8 shows anthropogenic traces on elements of the extremities.

Table S7 lists the observed cut marks as a result of different activities during the butchering process. These testify to the systematic separation of the connections between the zonoskeleton, stylo-, zeugo-, and autopodium. Traces appear repetitively as can be seen, for example, on the right and left *Femora*. Remarkable is also abundant evidence for disarticulation of hand and foot bones.

In addition to cut-marked bones, three skeletal elements show traces of gnawing by a medium-sized carnivore. These were observed on the proximal right *Scapula*, on a lumbar vertebra, and the *Os metacarpale II* and *III* sin., and produced when after butchering these bones were still in articulation. Carnivore modification did not lead to the disarticulation of the metacarpals (Figure S8).

Bone complex E10

Bone complex E10 is described by Mania as consisting of the primary context remains of a large individual that had been disarticulated by humans and was in an advanced stage of decay. The individual body parts were distributed over a larger area, of 5x6 meters, and the anatomically correct position of particular body parts was still recognizable. Mania notes the absence of the skull and some long bones, elements that were not discovered even after subsequent spatial expansion of the excavation area. The complex is characterized by an almost complete torso in anatomically correct position (Figures S9-S10). The anterior part of the carcass with the cervical spine, including the first two cervical vertebrae, parts of the torso, and the anterior limbs lay eastwards, at a distance of 3 meters. The complete left leg in an anatomically correct position lay on the cervical spine (*23*). It is worth remarking that below the thorax of this animal, some of the gut content was still preserved (*59*). The animal suffered from hyper-ossification of the sacrum due to arthritis (*3*).

Later paleontological studies showed that bone complex E10 comprises the remains of altogether 3 individuals: E10A, a 47-year-old male whose average body mass was calculated to be 12,5 tonnes with a shoulder height of 395 cm, E10B, a 26-year-old female with a shoulder height of more than 3 meters and an estimated body weight of more than 5,5 tonnes and an indeterminate additional individual (E10C) (*3*, *24*). E10A is represented by a vertebrate column with most of the ribs and the *Pelvis*, nearly complete left and right forelegs with only the right *Scapula*, and nearly complete left hindleg with only the left *Femur* preserved. Larramendi et al. (*1*) reconstructed the female E10B based on the *Os sacrum* and a nearly complete additional *Pelvis*.

Altogether, 223 NISP (125 MNE) were analyzed for bone surface modifications: E10A - *Atlas, Epistropheus,* cervical vertebrae 3-7, 13 thoracal vertebrae, 2 lumbar vertebrae, 3 caudal vertebrae, 3 vertebrae fragments, *Pelvis* sin./dex., 29 Ribs (among these 6th-7th and 16th Rib sin. and 14th-19th Rib dex.), *Scapula* dex., *Humerus* sin./dex., *Radius / Ulna* sin./dex., *Os carpi radiale* dex., *Os carpi intermedium* sin./dex., *Os carpi ulnare* sin./dex., *Os carpi accessorium* sin., *Os carpale I-III* sin., *Os carpale II-IV* dex., *Os metacarpale I-V* dex., *Os metacarpale II-V* sin., *Femur* sin., *Patella* sin., *Tibia* sin./dex., *Fibula* sin./dex., *Talus* dex., *Calcaneus* dex., *Os tarsale III-IV* dex., 9 sesamoids, *Os metatarsale III-V* dex., *Phalanges proximales, dig. I-V* dex., *Phalanges proximales, dig. III-IV* sin., 2 *Phalanges mediae, dig. II* dex., *Phalange media, dig. V* sin., *Phalange media* indet., *Phalange distale* sin. and 7 long-bone fragments.

Individual E10B was identified through the presence of a complete *Pelvis* with *Os sacrum*. Individual E10C was defined due to the presence of a 5th cervical vertebra, *Tibia* dex., *Os carpi intermedium* indet., *Os carpale IV* dex. and an *Os metatarsale III* dex..

The carcass of individual 10A survived in large parts, in contrast to individual 10B, only represented by a *Pelvis* with attached *Os sacrum*. Due to the additional presence of a *Tibia* and some autopodium bones and a cervical vertebra, the third individual within the bone complex was identified.

In total 48 specimens (38 MNE) showed signs of anthropogenic modification. Table S9 lists the observed cut marks as a result of different activities during the butchering process. The detailed analysis of the cut marks shows that the carcass was dissected into its smallest parts. The butchery sequence shows acquisition of the tenderloin and the dissection of the thorax. Cut marks on the midshafts of the right 14th-16th and 18th ribs could indicate that the inside of the intact ribcage was treated with stone implements after the torso was broken open, the viscera removed and the carcass rested on the right side. On the ribs of the left half of the body, the cut marks are lateral, e.g., on the midshafts of the 6th, 7th, and 16th ribs. If one follows the above interpretation, one would have to postulate a position of the torso on the left

side. Cut marks on the lateral surfaces of the spinae of vertebrae, which were created during the removal of the tenderloin, were probably also created while the torso rested on the left side.

The deboning, defleshing and systematic dissection of the skeleton into its individual components of the right and left halves and the evisceration of the carcass are attested. It is remarkable that here, as in the previous examples, the dismemberment of the hands and feet is evident and that none of the skeletal elements was opened for the removal of bone marrow.

The *Pelvis* with attached *Os sacrum* attributed to individual 10B also shows cut marks. They indicate the deboning of the right hind leg for this individual. Table S10 lists the observed cut marks as a result of different activities during the butchering process. For E10C none of the bones were cut marked.

The bones of individual 10A show not only anthropogenic traces but also those of modification by medium- and large-sized carnivores. A total of seven bones are affected. The spinae of two thoracic vertebrae, one of them with cuts, show slight traces of gnawing proximally, as does the distal end of a rib. In addition, the *Os metacarpus II* sin., the *Phalange proximale, dig. III man*. sin. and the *Os metacarpus V* dex. are punctured distally. Finally, on the right *Tibia*, which was already mentioned in context with the cut marks, traces of gnawing can be seen at the proximal end. Distally, the bone shows a modern fracture.

Unit 6.1 – lower littoral zone (PAZ IV of the Eemian Interglacial)

Bone complex E23

Bone complex E23 was excavated from the southern part of the main find horizon, the lower littoral zone (unit 6.1), located next to complexes E21 and E24. Mania assumes that all three originally formed one large complex (*23*). Remains from altogether three individuals were scattered over an area of 6x6 meters. E23A, a large male, 44 years of age with an estimated body mass of 9,8 tonnes and a shoulder height of 363 cm, was associated with remains of an additional, even larger, 50 years old male individual of 13 tonnes and a shoulder height of 400 cm (E23B), as well as a small female, younger than 26 years, with an estimated 2030 kg body mass (E23C) (*1*, *24*). This bull, E23B, represents one of the largest fully grown individuals excavated at Neumark-Nord 1.

Concerning the find-situation (Figures S13-14), the anatomical order of body parts was still recognizable though not in anatomical connection. The bull (E23A) lacked its skull, but *Atlas* and *Os hyoideum* with ossified traumatic fracture (*3*, *24*) as well as some cervical vertebrae were distributed all over the area (*23*). The *Pelvis*, spine and ribs were more or less in anatomical position. Only a few vertebrae were in direct anatomical association. Mania counted 26 ribs and 27 vertebrae, excluding the tail (*23*). The *Scapula* was discovered at a distance of 4 meters. Skulls, tusks and long bones were also scattered over the area covered by the bone complex (*23*).

In their study of E23A Larramendi et al. (*1*) included a nearly complete vertebral column, *Os sternum*, most of the ribs, complete *Pelvis* and partial appendicular skeleton elements. From E23B, the other male individual of this bone complex, part of the skull and the fragment of a left tusk was studied. For the last molar, Palombo et al. (*3*) describe a hole on the anterior side of the occlusal face, probably caused by acid food and still containing plant remains.

Bones studied for bone modifications comprised a NISP of 486 (95 MNE) (Table S11). The following elements were studied for Individuals E10A-10C: Cranium with M3 sup. dex. and tusk, Mandible, *Os hyoideum* dex., *Atlas* and cervical vertebrae 3-7, thoracic vertebrae 1-6, 8-9, 11-16, 18-19, lumbar vertebrae 3-4, caudal vertebrae 1, 2 and 4. From the right side of the

rump rib 1, 3-6, 8-14, 16-17 survived, from the left side rib 1-2, 5-9, 12-17, 26 additional rib fragments and the *Scapula* survived. The foreleg is represented by *Humerus* dex. and *Radius* sin., *Os carpi intermedium* sin., *Os carpale II* and *III* sin., *Os carpale III* dex., *Phalanges proximales, dig. III-IV, Phalanges distales, dig. III-IV*. The right hindquarter with *Os sacrum, Pelvis, Femur, Fibula* and *Calcaneus* was recorded in addition to *Os metatarsale I* sin..

Cut marks were recorded on 34 NISP, representing an MNE of 33 (Table S11). The majority of cut marks were observed on vertebrae and ribs of individual E23A. These traces testify to the complete dismemberment of the torso. The exploitation of the tenderloin is evident here as well as the removal of skin, fat and the connecting tissue between the ribs. This process was meticulously carried out from the outside of the carcass but also from within the area of the inner rib cage. In addition, marks refer to the disarticulation of the entire spine and rib cage. Traces on long bones prove the separation of the stylo-, from the zeugo-, from the autopodium after the right front leg and the left hind leg have been deboned. Finally, cuts on bones of the left metacarpus indicate its disarticulation.

Carnivore traces were additionally observed on bones from bone complex E23. From individual E23A, the cervical spine is more or less completely preserved (Figure S16). The bones show gnawing marks on the right side of the transverse process. The character of the marks suggests that the cervical spine was still articulated at the time when carnivore gnawing occurred. The same is true for the distal ends of a 3rd, 5th, and 6th rib, which show carnivore tooth marks.

Unit 7 (PAZ V, the *Carpinus* phase of the Eemian Interglacial)

Bone complex E30

Bone complex E30 was located in the eastern part of the lake basin, in the lower part of the upper “gewarvte Feindetritusmudde*” sensu* Mania (*23*). He (*23*) describes bone complex E30 as containing the remains of a large adult elephant, which probably became embedded more or less completely. Although the skull was preserved in numerous fragments, the tusks were missing. The fragment of a flint flake was uncovered among the bones (Figure S17). On its dorsal and ventral sides, remains of crushed oak bark were identified, originally interpreted as remains of a hafting residue, an interpretation disputed by Koller and Baumer (*60*) who suggest that the crushed oak bark may have been used for the tanning of hides.

Table S13 lists the skeletal elements (178 NISP/79 MNE) which were studied for bone surface modifications. Among these are the following skeletal elements: Cranium fragments, Mandible sin., vertebrae fragments, *Atlas, Epistropheus*, cervical vertebrae 4-7, thoracal vertebrae 1-19, lumbar vertebrae 1 and 3 and caudal vertebrae, *Scapula* fragments, rib fragments, ribs 1, 3- 4 sin. and ribs 1, 3 dex., *Pelvis* fragments, *Humerus* indet, *Radius* sin./dex., *Ulna* indet., *Femur* dex., *Tibia* sin., *Fibula* dex., *Os carpale I* sin., *Phalange proximale, dig. I* and *III*, *Os metacarpale III* sin., *Calcaneus* sin./dex., *Os tarsi centrale* sin./dex., *Os tarsale III* dex., *Os metatarsale II - III* dex. and *Phalange proximale, dig. II* dex.

Cut marks were observed on 6 bones, constituting 5 skeletal elements. Even though not many anthropogenic traces were observed, the traces present indicate the disarticulation of major parts of the carcass. Particularly remarkable are cut marks observed on the condyles of the occipital, next to the opening of the occiput. These marks are particularly deep, cross each other and are confined to a small area. They suggest that the severing of the skull was done by the disarticulation at the atlas. Cut marks on the *Scapula* show that the right half of the carcass was dissected and traces on ribs indicate the disarticulation of the ribcage and the removal of fat and connective tissue between the ribs (Table S14). Finally, as observed in all complexes illustrated here, the feet were disarticulated as a means to harvest the rich fat resources stored

in the elephant’s foot cushion. Traces of carnivore modification were not observed on bones from E30.

Supplementary Text 2: Calculating estimates of caloric and nutritional yields of *P. antiquus* from Neumark-Nord 1

Given the very large sizes of the Neumark-Nord straight-tusked elephants and their extensive processing, they undoubtedly yielded large amounts of food. Based on studies of contemporary elephants it is possible to set some constraints on the nutritional yields provided by these elephants.

African elephant carcasses average 38% muscle tissue/weight, with little variation, and including brain, tongue, liver, heart, and trunk, so the edible mass (omitting stomach content, etc.) makes up 42% of body weight (*61*), a more generous estimate than an earlier one by Frison and Todd (*62*) (25-35%). For a 10-tonne Neumark-Nord male elephant this implies 4200 kg of edible material.

However, there are constraints on the consumption of meat, which should be taken into account when discussing the food yielded by the Neumark-Nord elephants. In general, mammal tissue is rich in proteins, relatively low in fats (although high amounts are stored in bone marrow and adipose tissue), and contains almost no carbohydrates. An unbalanced intake of these macronutrients, in the form of a diet without sufficient non-protein calories (i.e. calories from either fat or carbohydrates), can lead to severe health problems including a condition known as "rabbit starvation" (*37*, *38*). Protein should generally not exceed 25-35% of the daily macronutrient energy intake (en%) (*38*, *63*), a proportion which is reflected in hunter-gatherer diets (*37*, *38*, *64*, *65*). A Neumark-Nord elephant carcass – as well as those of all other mammals - could therefore come with a considerable excess of protein, when all edible parts are considered.

The body fat percentage of the Neumark-Nord elephants is unknown, but studies of current elephants shed light on the range of likely values. Studies of captive elephants have documented body fat percentages of 5.24 to 15.97% for *Loxodonta africana* (*66*), and a range of 3.54 – 24.59% for Asian captive elephants, *Elephas maximus* (*67*).

Based on this we can suggest the likely energy yield in fat for the elephants in this study. For a 10-tonne Neumark-Nord male elephant, a conservative estimate of 3.5% body fat would yield minimally 350 kg of fat, probably the lower limit of variation throughout a year. Based on Byer's and Ugan's (*61*) calculations as well as the body fat percentages discussed above, a 10-tonne elephant would yield 4200 kg of edible material, including minimally ~350 kg of fat. With 100 g fat translated into ~660 kcal (*33*) that would imply that our Neumark-Nord male would have stored *minimally* 2.3 million kcal in the form of fat only. Continuing this thought exercise, and assuming a high daily energy expenditure (DEE) of 4000 kcal/day for an "average" Neanderthal ((*42*), p. 326-27) we are talking about minimally 575 daily rations financed only by the lipids coming from one large elephant.

Further, taking the 'protein ceiling' into account, we can estimate the amount of meat and fat that would be available for consumption. Following Speth's calculations (*38*), and assuming our average Neanderthal would have an upper protein intake level of 300 g/day at 4 kcal/g and meat consisting of 20% protein, this would imply a daily safe maximum intake of 1500 grams of "meat", yielding 1200 kcal. Combining the meat plus lipids into a 4,000 kcal "protein safe" elephant-fat and -meat package would yield >821 daily rations, a very conservative estimate, based on the lowest values of the documented body fat range. In such an "elephant-products only" scenario, i.e. without alternative sources of key macronutrients, about 3,000 kg of "meat" would not be usable. Increasing the body fat content to 10% (a value which is still in the middle of the range calculated for contemporary African elephants) would yield about 2,500 "elephant-only" daily portions in our calculation, with a good 1,000 kg of muscle meat still unused. Raising the fat content of our hypothetical elephant to 12.8%,

still below the middle of the range of Asian elephants, would enable complete and safe consumption of this elephant, yielding >3,000 adult Neanderthal rations of 4,000 kcal.

This is of course a thought exercise rather than an exact value, which is intended to illustrate the quantity of food yielded by the Neumark-Nord elephants, even taking nutritional constraints into account.

Edible material from elephants was likely supplemented with lipids obtained from other animals, e.g. by marrow-processing activities documented at the nearby location of Neumark-Nord 2 (*17*), or e.g. with carbohydrates from plant foods. While the processing of large herbivores created a clear archaeological signal in the Neumark-Nord landscape and at Neanderthal sites in general, nutritional studies indicate that they could not have survived on terrestrial game alone and that plants played an important role in Neanderthal diets, providing carbohydrates and some of the required nutrients and calories (*68*, *69*). Recent studies suggest that starch-rich foods were already important before the split between the Neanderthal and modern human lineages (*70*), and document consumption of a similarly wide range of plant species by Neanderthals across their geographical range (*69*, *71*). The plant component of the Neanderthal diet at Neumark-Nord remains largely unknown, as is the case at most Palaeolithic sites, no matter how important plant foods must have been in the past, especially in the context of the protein ceiling. At Neumark-Nord, fragments of charred hazelnut (*Corylus avellanus*), acorn (*Quercus* sp.), and blackthorn/sloe plum (*Prunus spinosa*) (*72*) may constitute the ephemeral traces of this part of Last Interglacial Neanderthal diet (*22*).

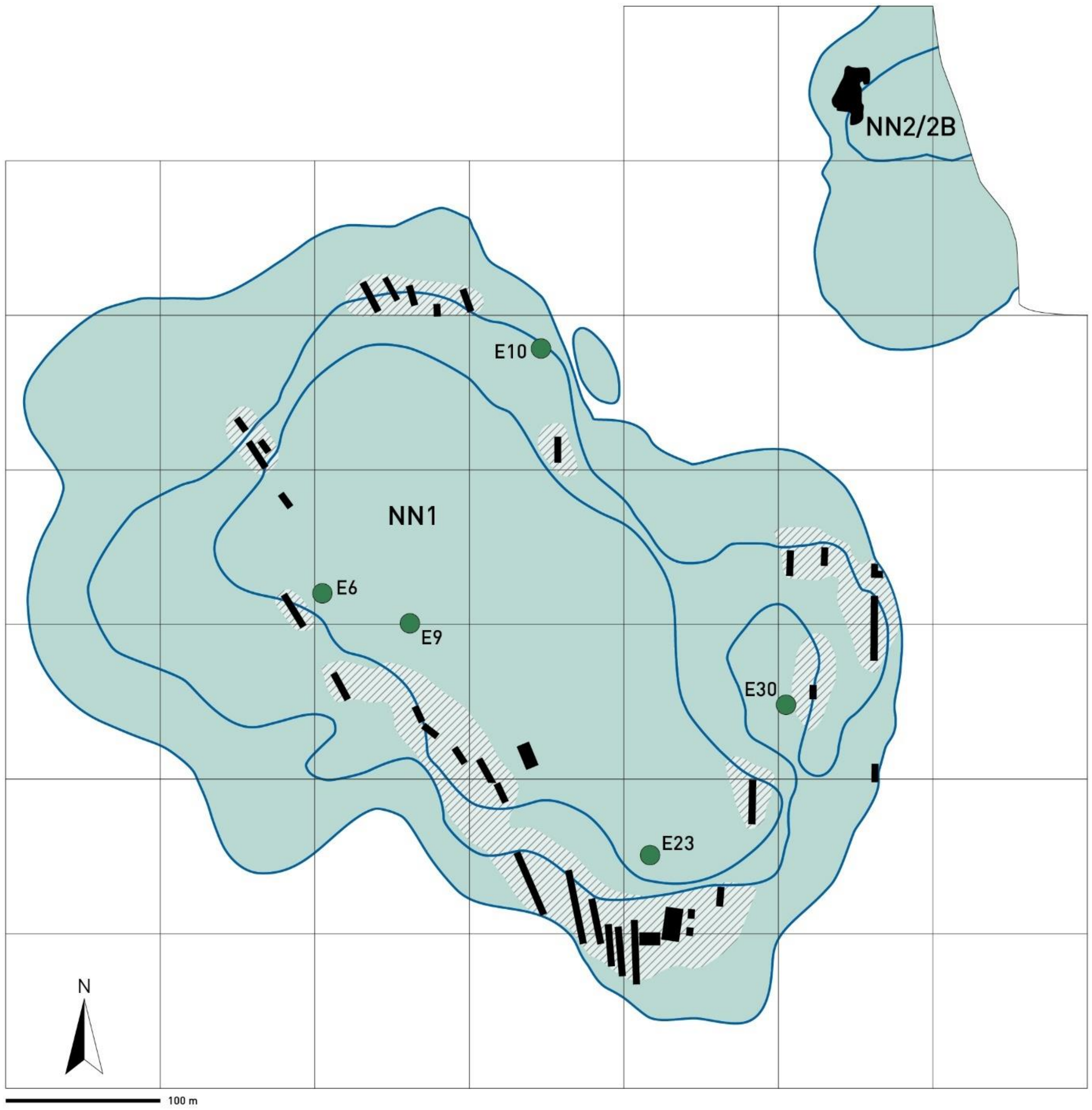

Fig. S1

Topographic map of the basin Neumark-Nord 1 (center) and Neumark-Nord 2 (upper right) with the spatial distribution of bone complexes (dots) mentioned in the SI from units 4, 6.1, 6 and 7 (after Mania (*23*) and archeological rescue interventions (black rectangles), the high-density distributions of flint artifacts and fragmented faunal remains (hatched areas), and the position of the Neumark-Nord 2 excavated area (in black) (after (*22*). Contour lines of the basins, at respectively 8, 16, and 24 m below the surface. The outer line indicates the maximum extension of the water body.

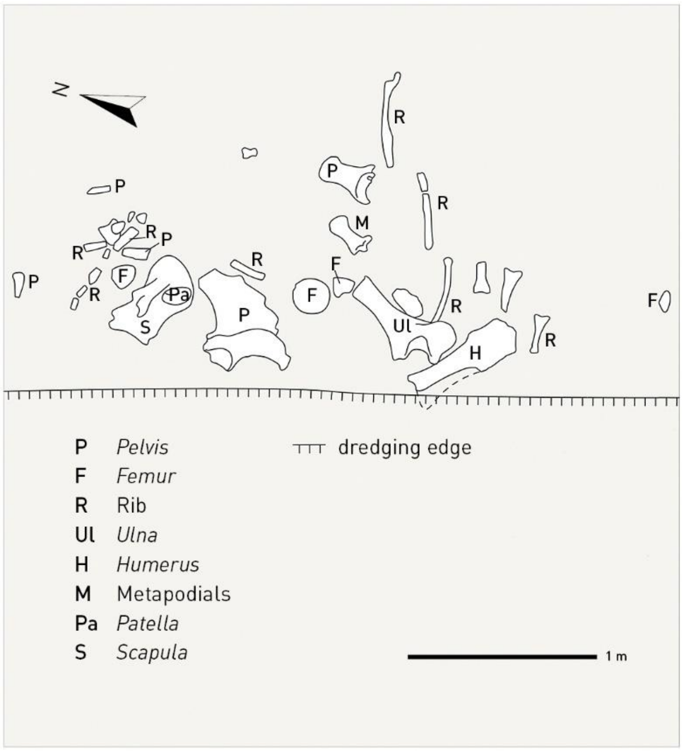

Fig. S2

Individual E6, spatial distribution of skeletal elements modified after (*23*), Figure 2). (© Landesamt für Denkmalpflege und Archäologie Sachsen-Anhalt, Dietrich Mania)

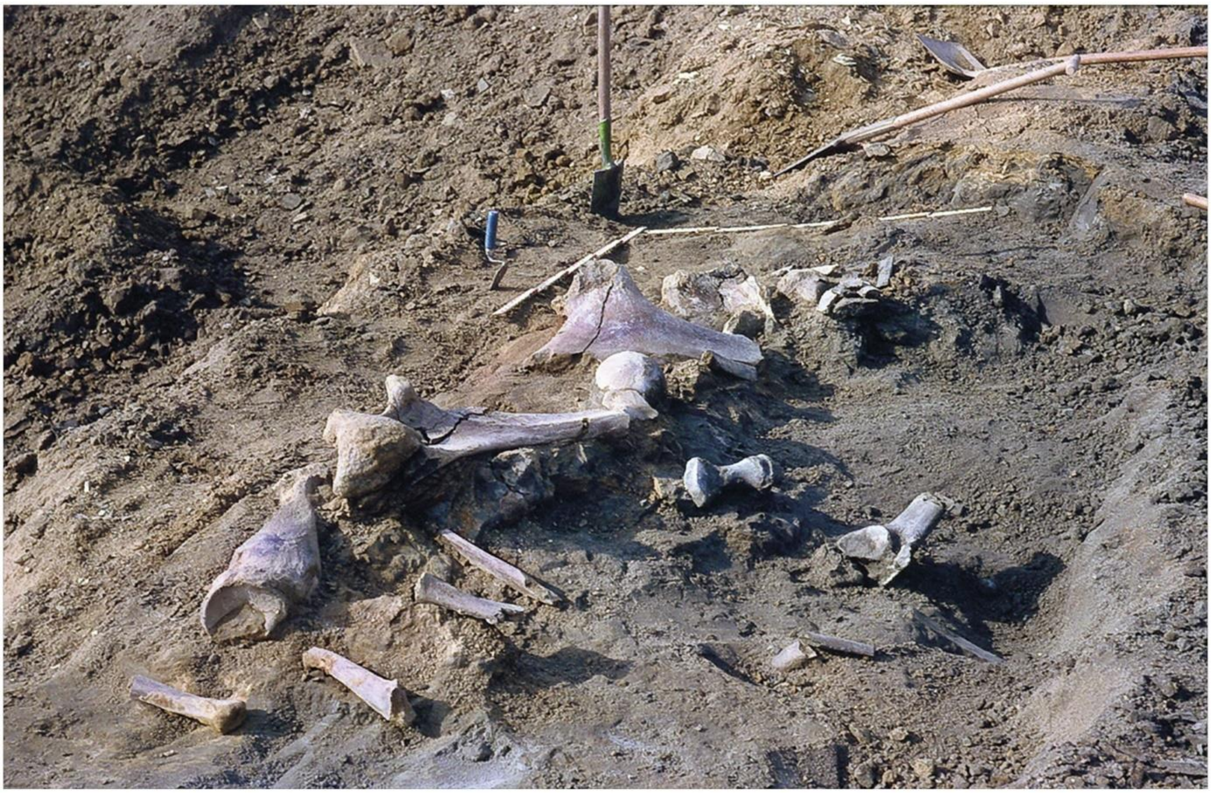

Fig. S3

Individual E6, view from South-East. (© Landesamt für Denkmalpflege und Archäologie Sachsen-Anhalt, Photo Dietrich Mania)

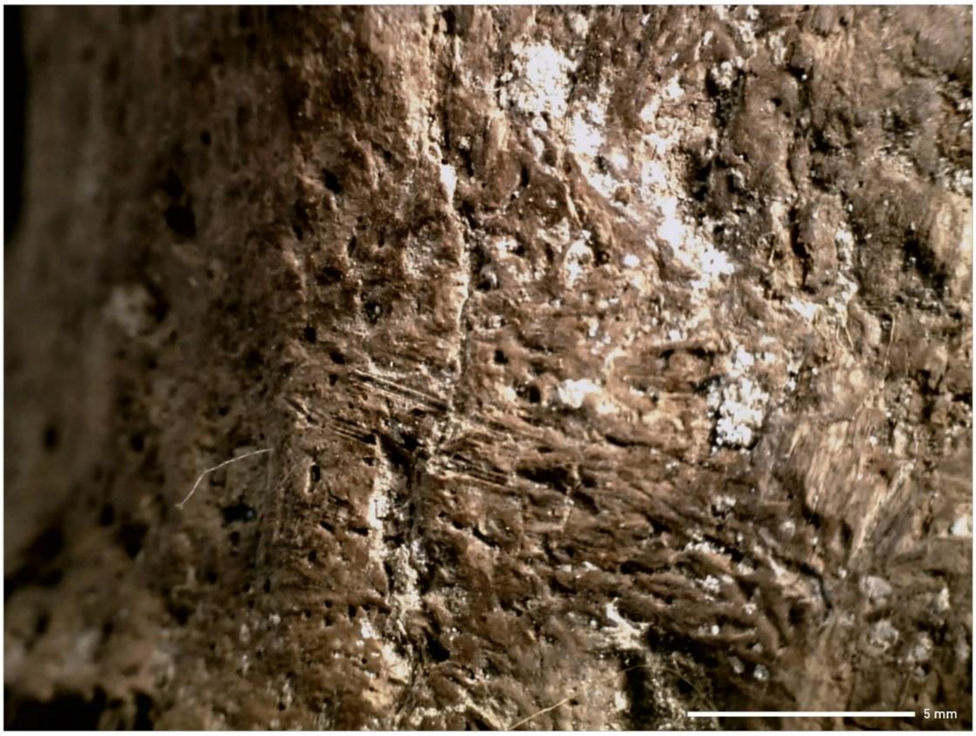

Fig. S4A

Cut marks on *Ulna* sin., cranial aspect. For a detailed description see Table S5.

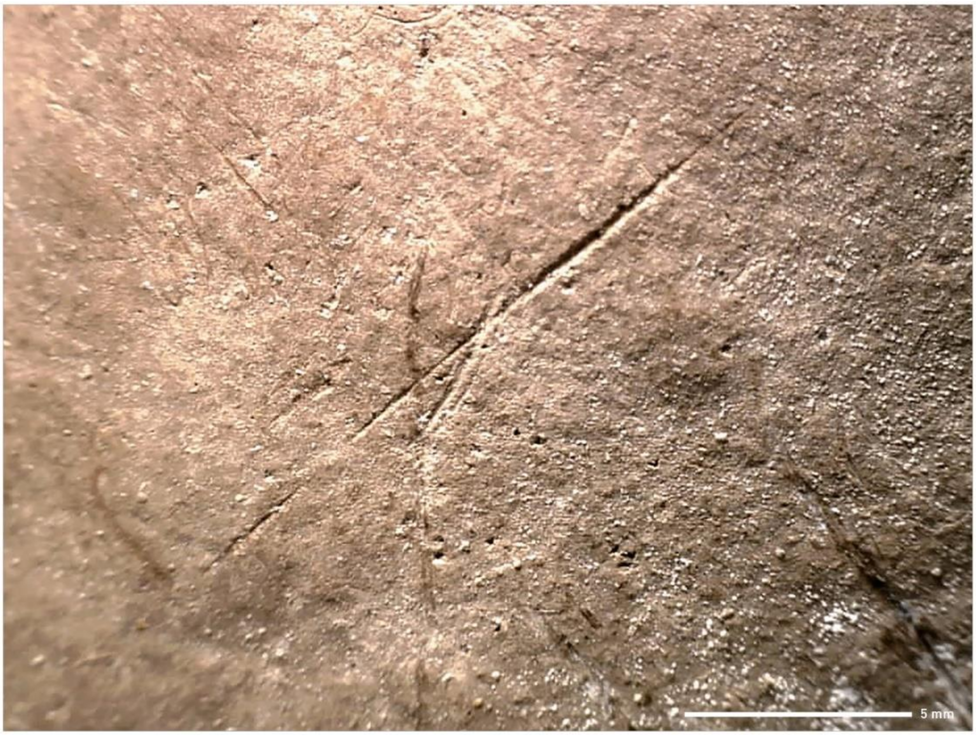

Fig. S4B
Cut marks on *Os carpi intermedium* sin., proximal aspect. For a detailed description see Table S5.

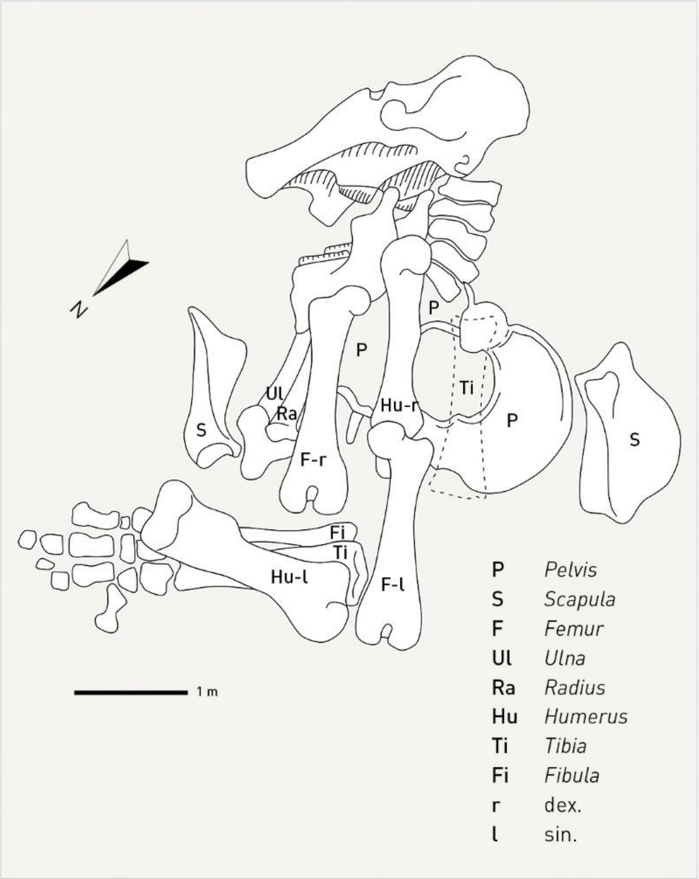

Fig. S5
Find-situation documented for E9 (modified after (*23*), Figure 6). (© Landesamt für Denkmalpflege und Archäologie Sachsen-Anhalt, Dietrich Mania)

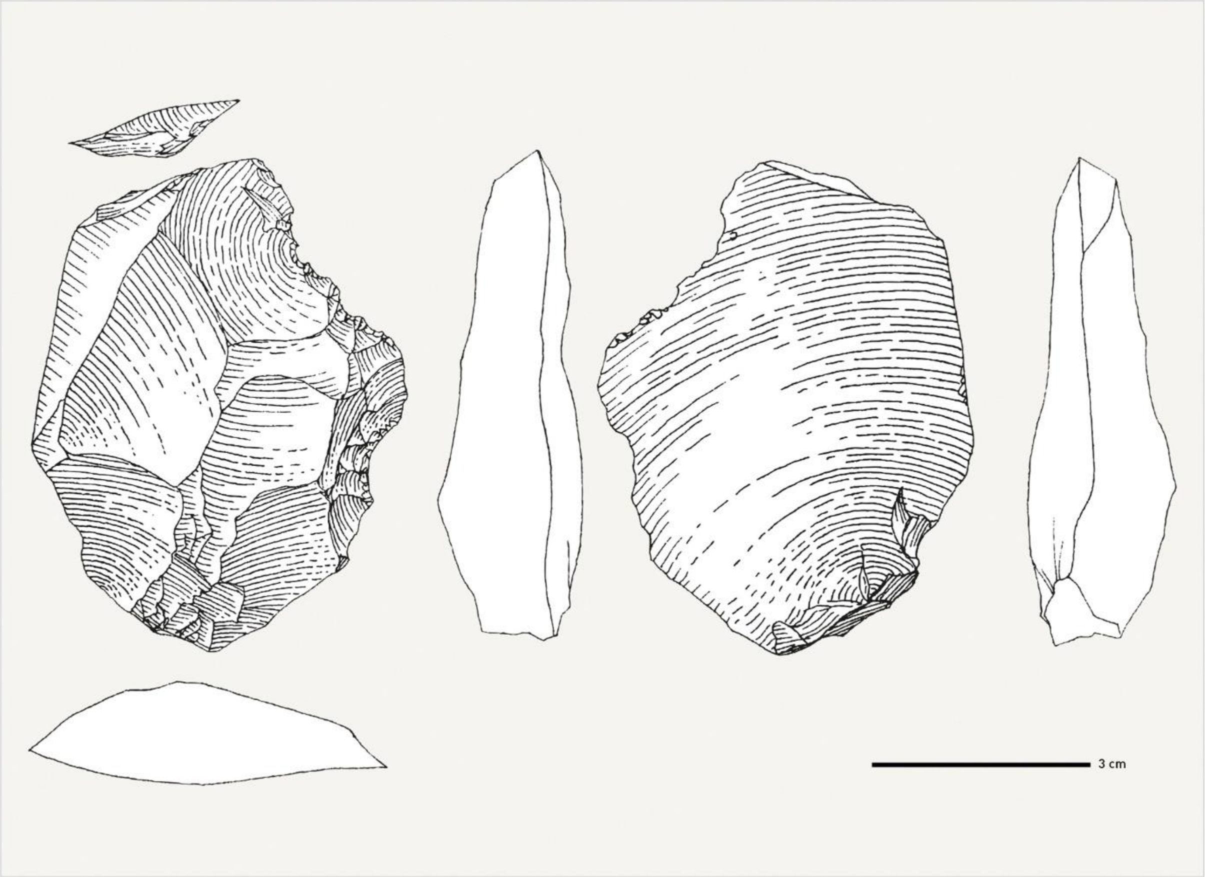

Fig. S6
Large flint flake found amongst the bones of E9. (© Landesamt für Denkmalpflege und Archäologie Sachsen-Anhalt, Dietrich Mania)

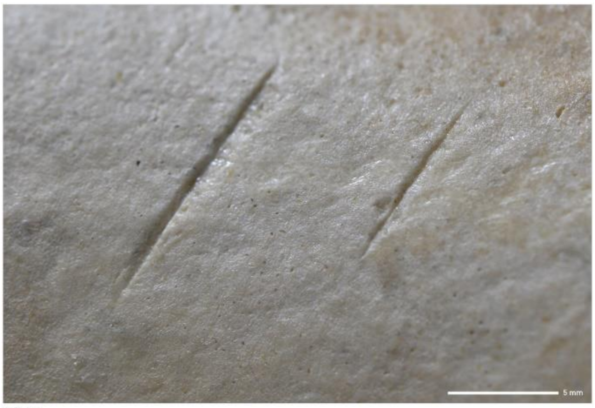

S7A1

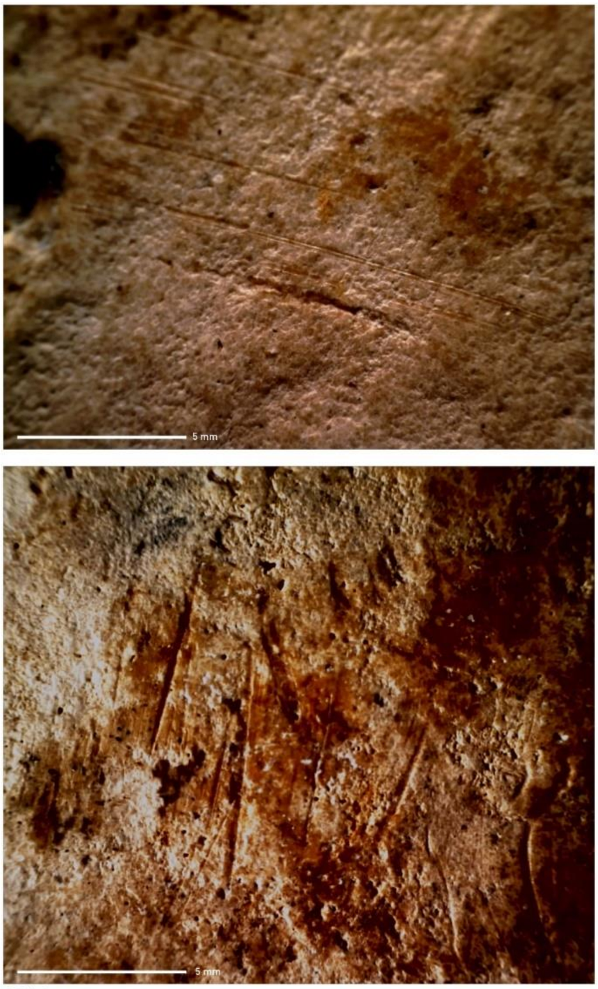

S7A2

Fig. S7A
Bone complex E9. Cut marks on *Ulna* sin. – proximal / cranial aspect. For a detailed description see Table S7.

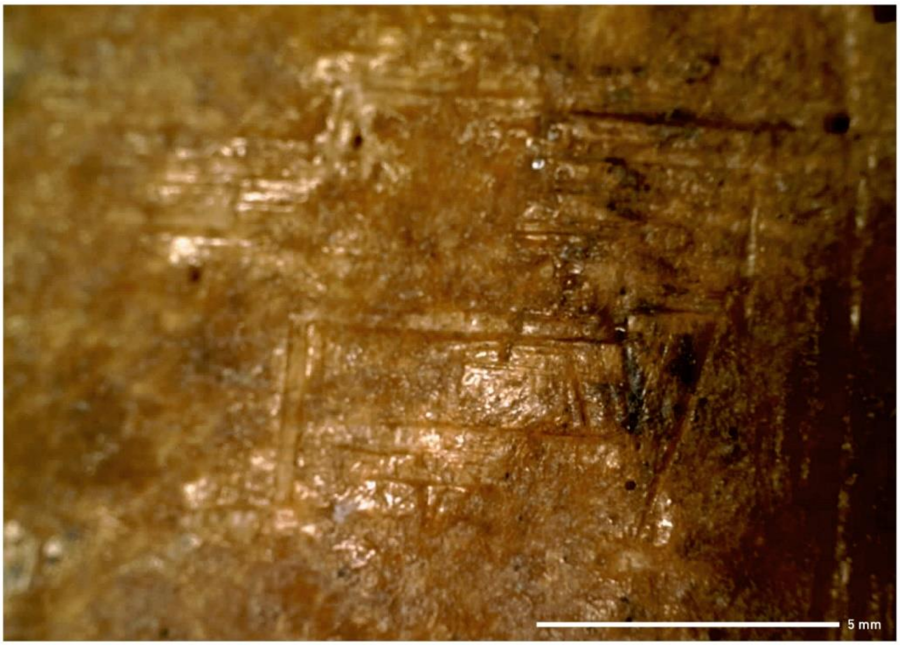

Fig. S7B
Bone complex E9. Cut marks on *Femur* dex. – distal / caudal aspect. For a detailed description see Table S7.

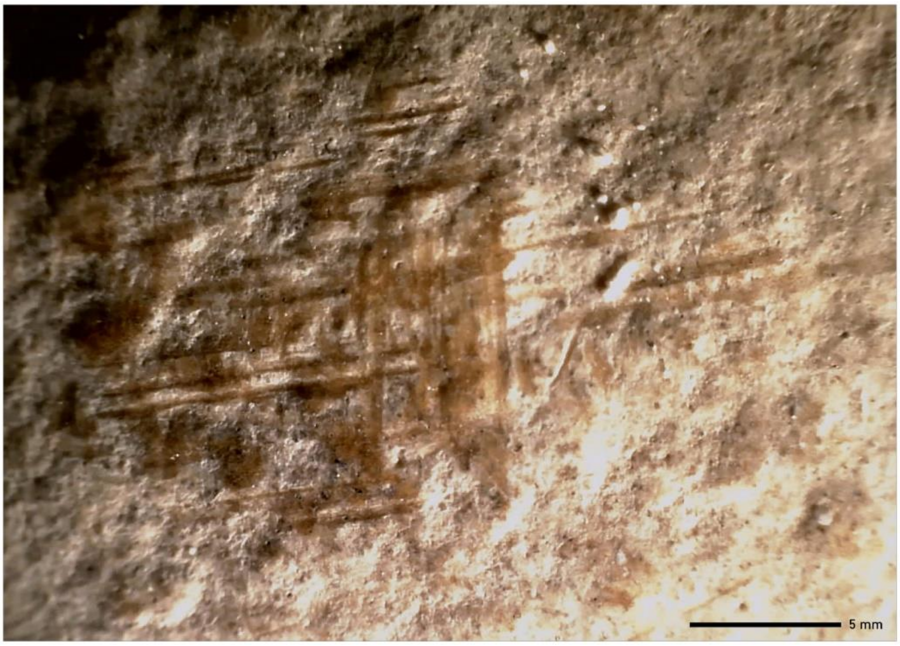

Fig. S7C
Bone complex E9. Cut marks on *Femur* sin. – distal / caudal aspect. For a detailed description see Table S7.

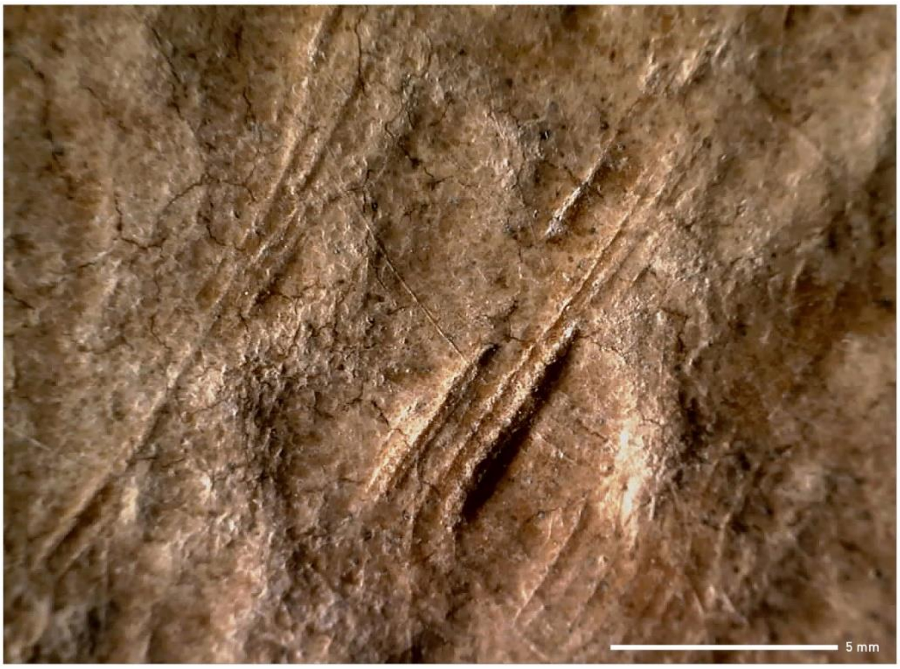

Fig. S7D
Bone complex E9. Cut marks on *Talus* dex. – cranial aspect. For a detailed description see Table S7.

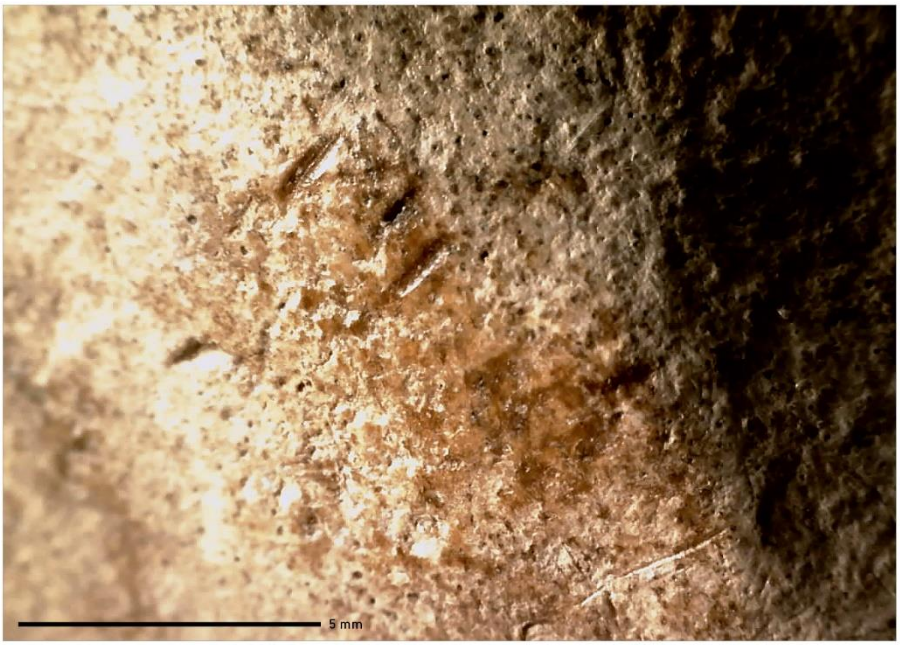

Fig. S7E
Bone complex E9. Cut marks on *Os carpale IV* dex. – distal aspect. For a detailed description see Table S7.

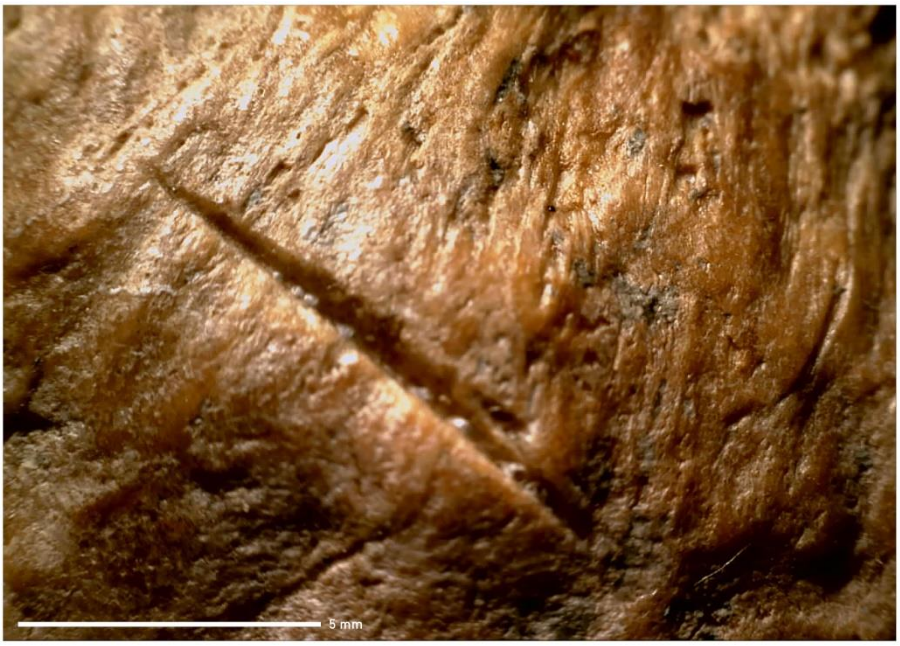

Fig. S7F
Bone complex E9. Cut mark on *Phalange proximale* sin. – dorsal/proximal aspect. For a detailed description see Table S7.

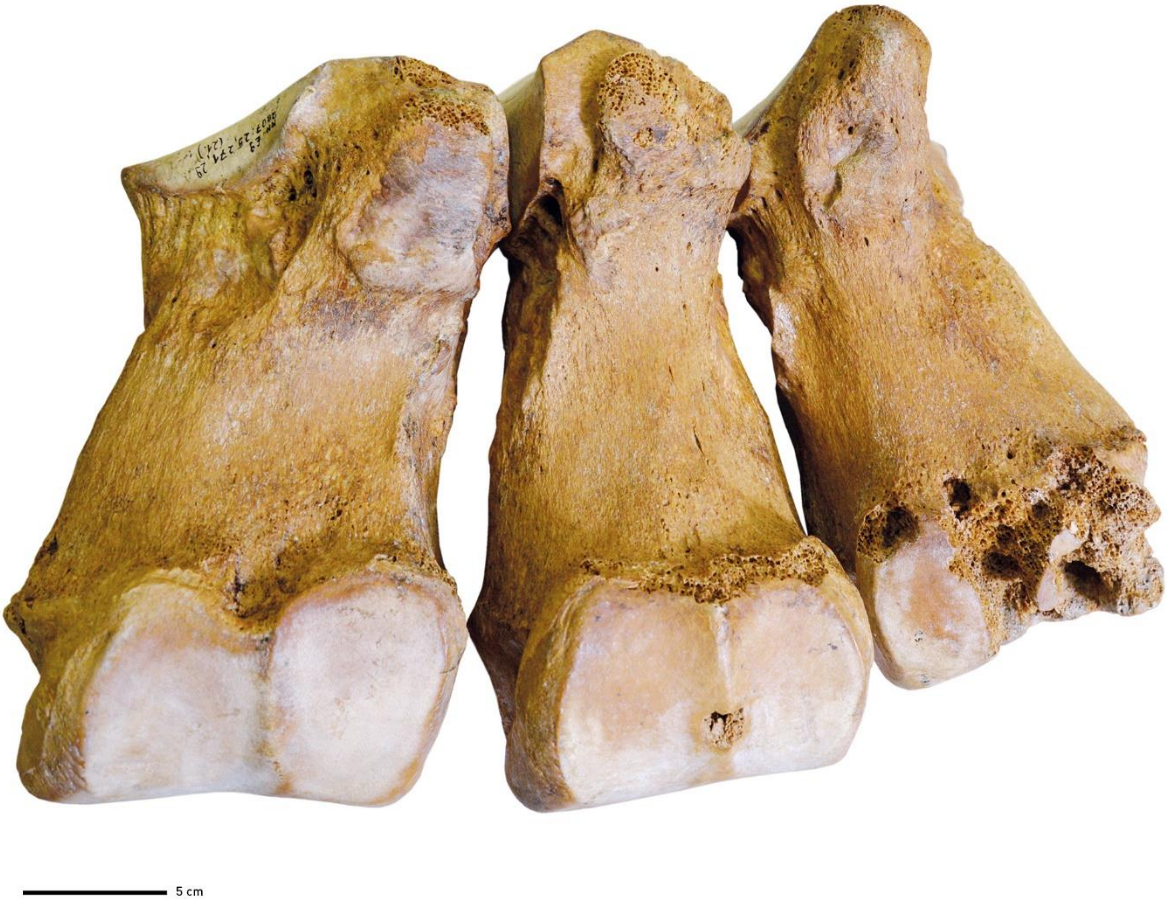

Fig. S8
Bone complex E9. Carnivore modification on metacarpals.

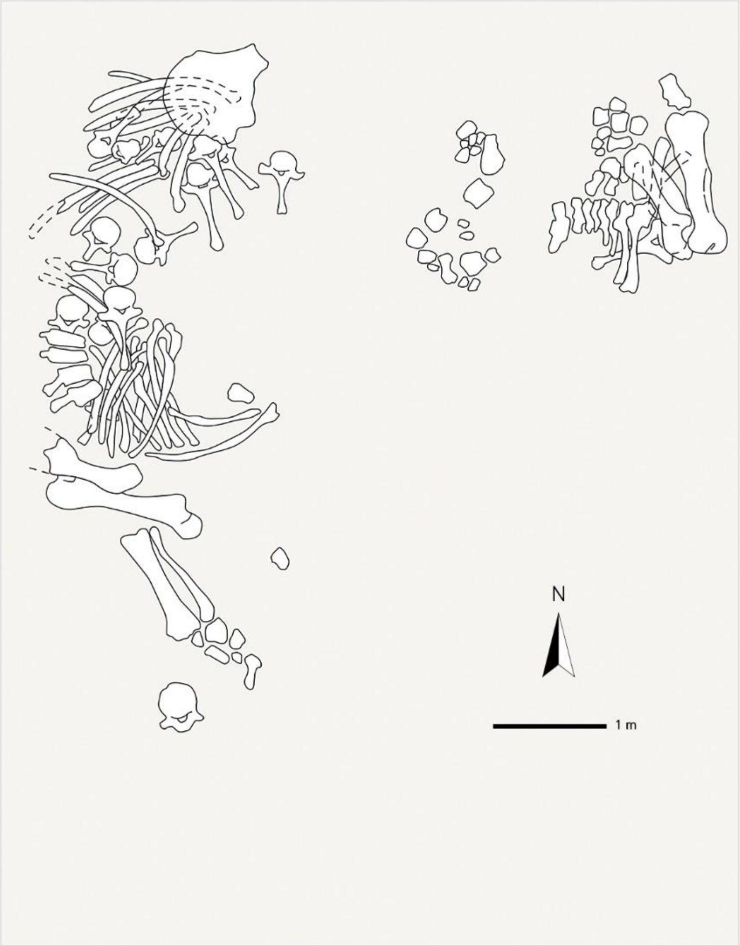

Fig. S9
Find-situation documented for E10. (© Landesamt für Denkmalpflege und Archäologie Sachsen-Anhalt, Dietrich Mania)

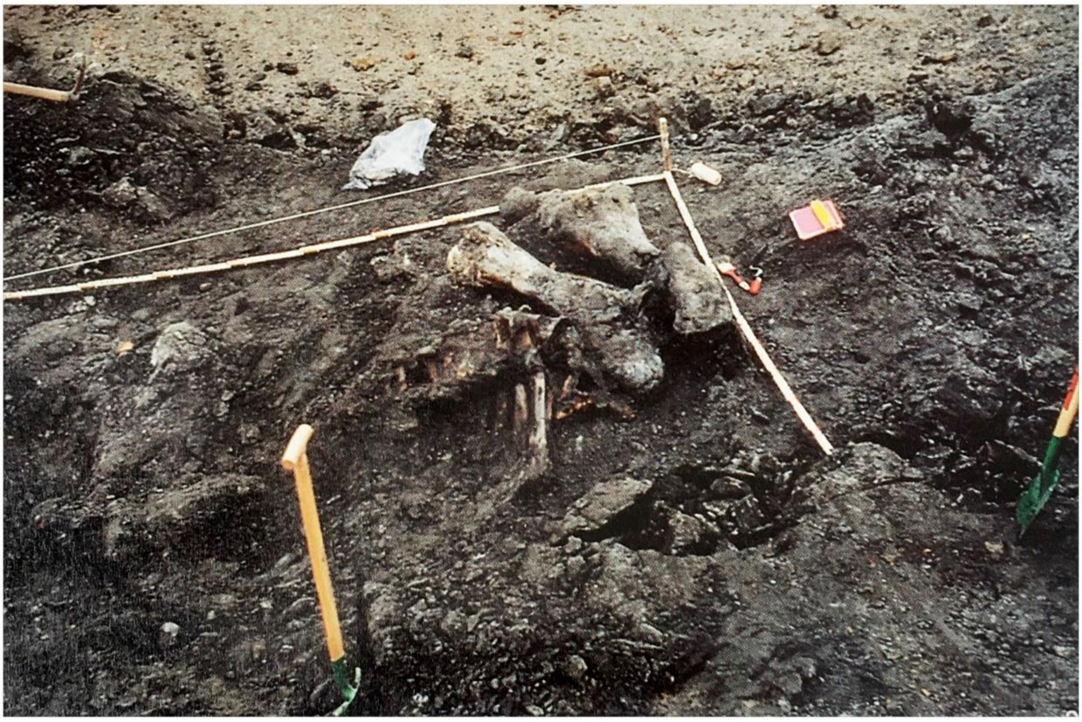

Fig. S10
Find-situation documented for E10, view from South-West. (© Landesamt für Denkmalpflege und Archäologie Sachsen-Anhalt, Photo Dietrich Mania)

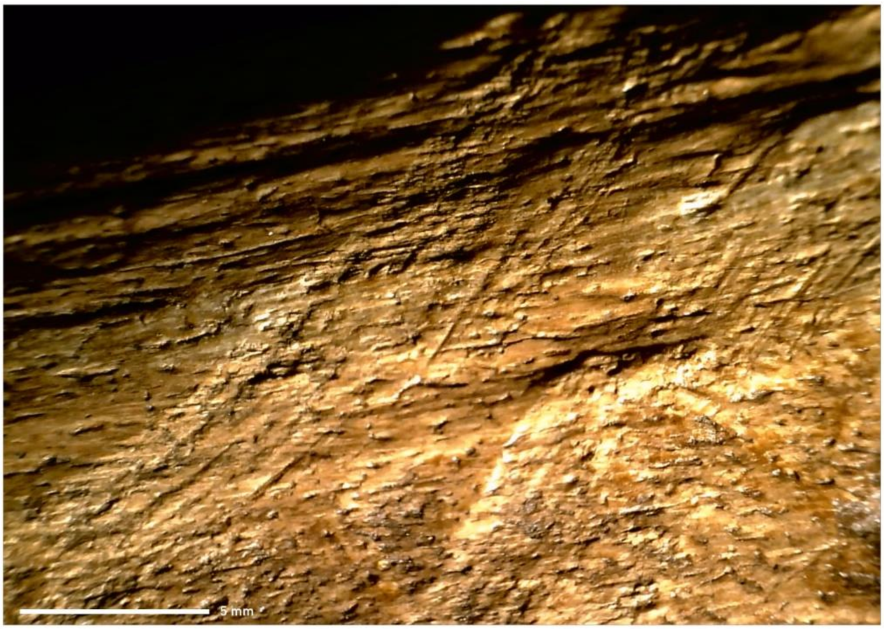

Fig. S11A
Bone complex E10, Individual E10 A. Cut marks on spina of 12/13th thoracal vertebra – dex. - lateral aspect. For a detailed description see Table S9.

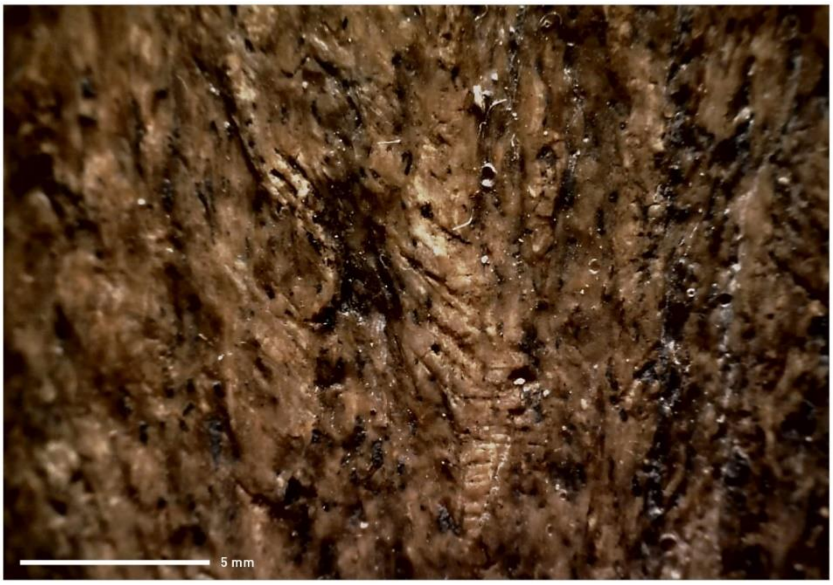

Fig. S11B
Bone complex E10, Individual E10 A. Cut marks on 7th rib - lateral aspect. For a detailed description see Table S9.

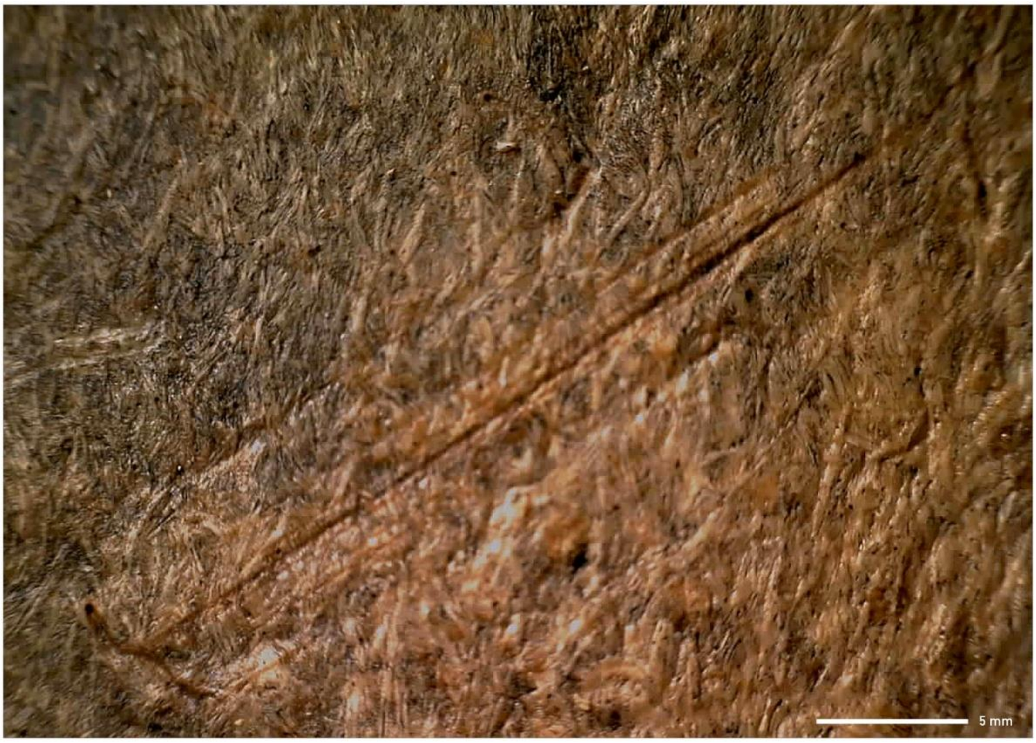

Fig. S11C
Bone complex E10, Individual E10 A. Cut marks on *Pelvis* dex. – *Os ilium* - medial aspect. For a detailed description see Table S9.

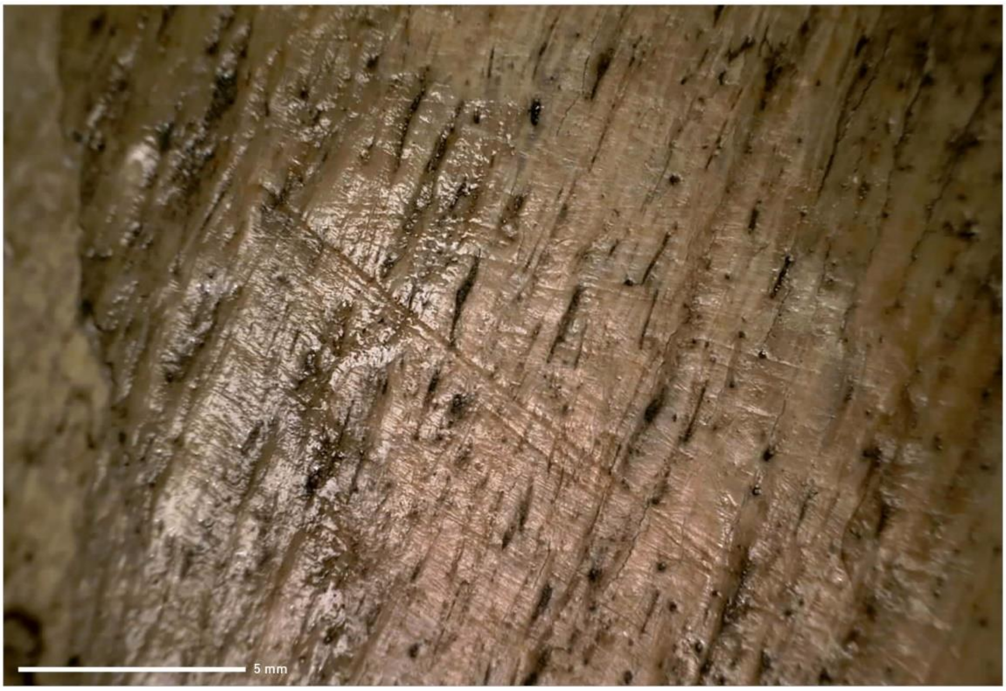

Fig. S11D
Bone complex E10, Individual E10 A. Cut marks on *Scapula* dex. - medial aspect. For a detailed description see Table S9.

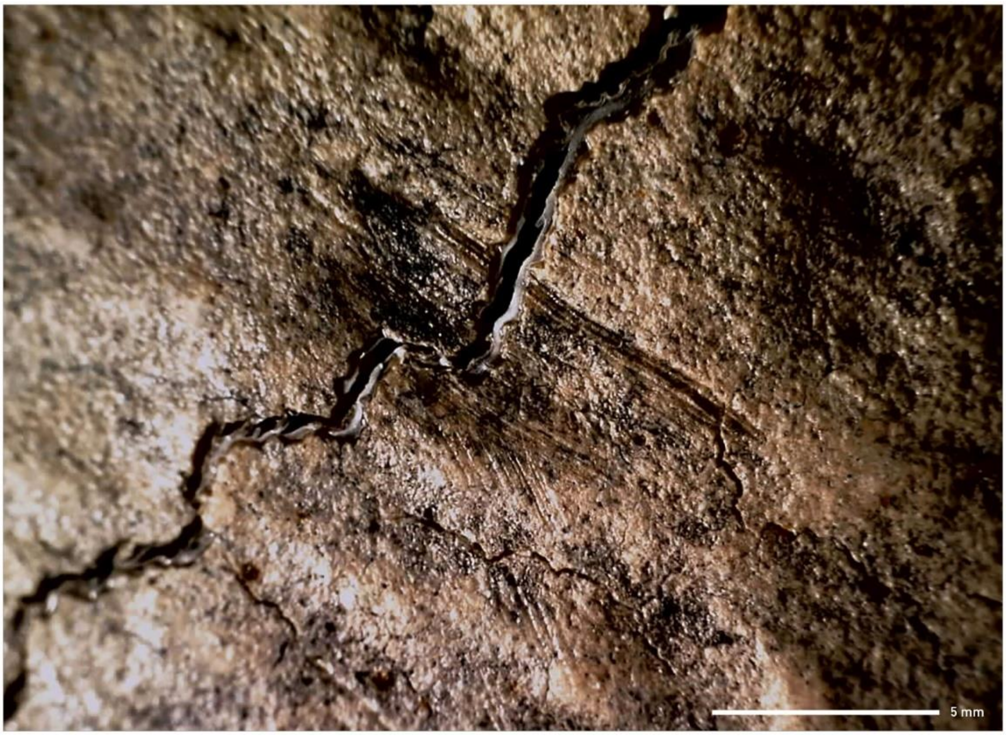

Fig. S11E
Bone complex E10, Individual E10 A. Cut marks on *Humerus* dex. – distal/cranial aspect. For a detailed description see Table S9.

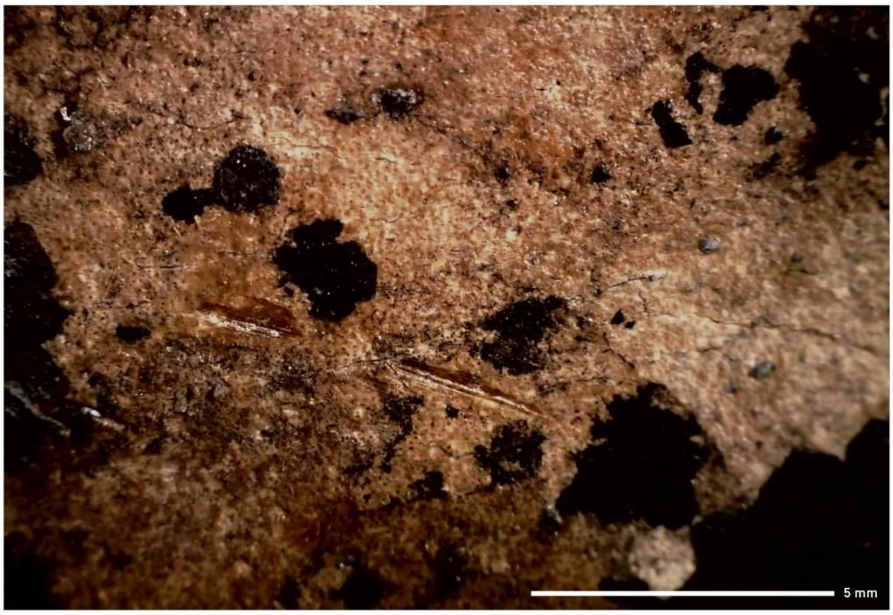

Fig. S11F
Bone complex E10, Individual E10 A. Cut marks on *Radius/Ulna* sin. - distal aspect. For a detailed description see Table S9.

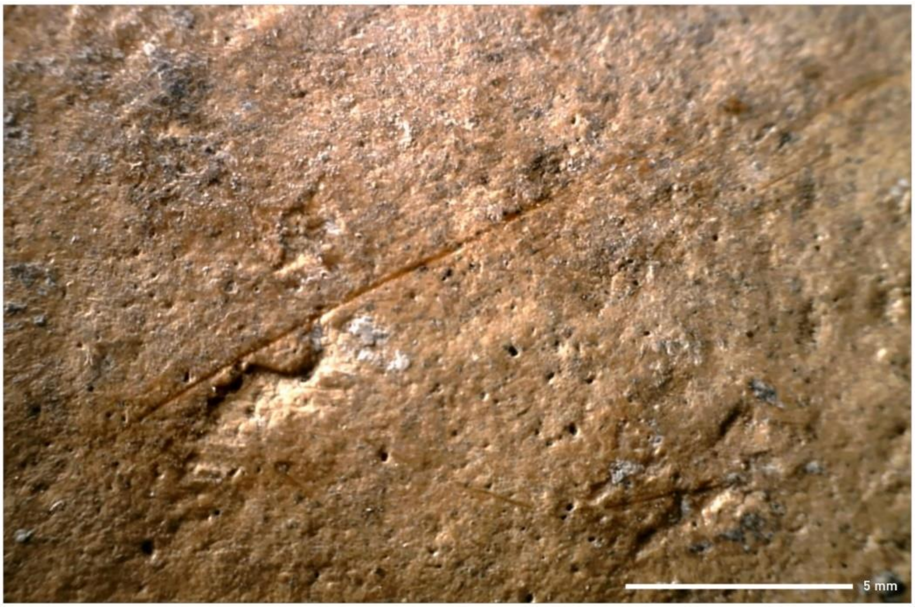

Fig. S11G
Bone complex E10, Individual E10 A. Cut mark on *Os carpi intermedium* dex. - proximal aspect. For a detailed description see Table S9.

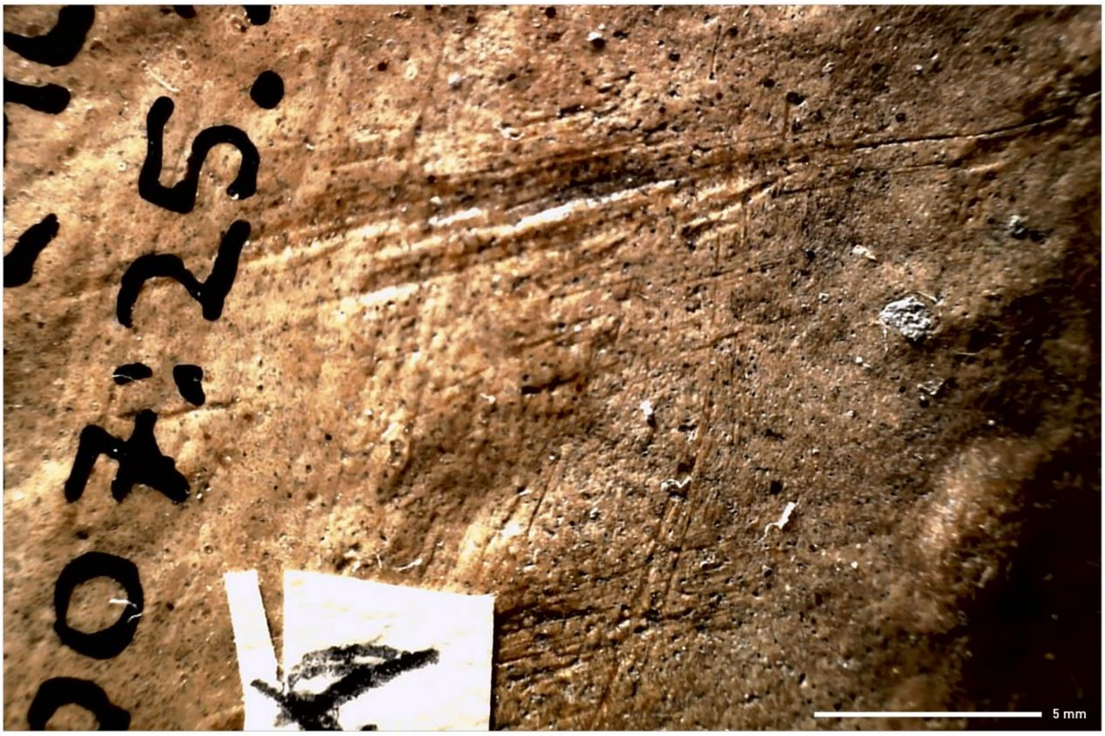

Fig. S11H
Bone complex E10, Individual E10 A. Cut marks on *Os carpi intermedium* sin. - medial aspect. For a detailed description see Table S9.

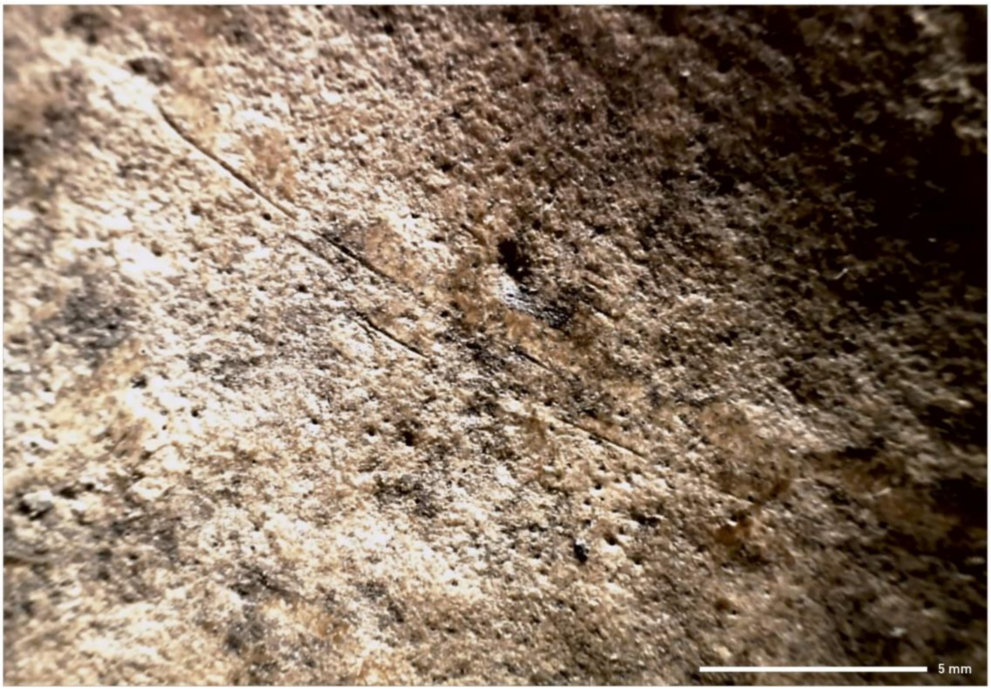

Fig. S11I
Bone complex E10, Individual E10 A. Cut marks on *Os carpi ulnare* sin. - proximal aspect. For a detailed description see Table S9.

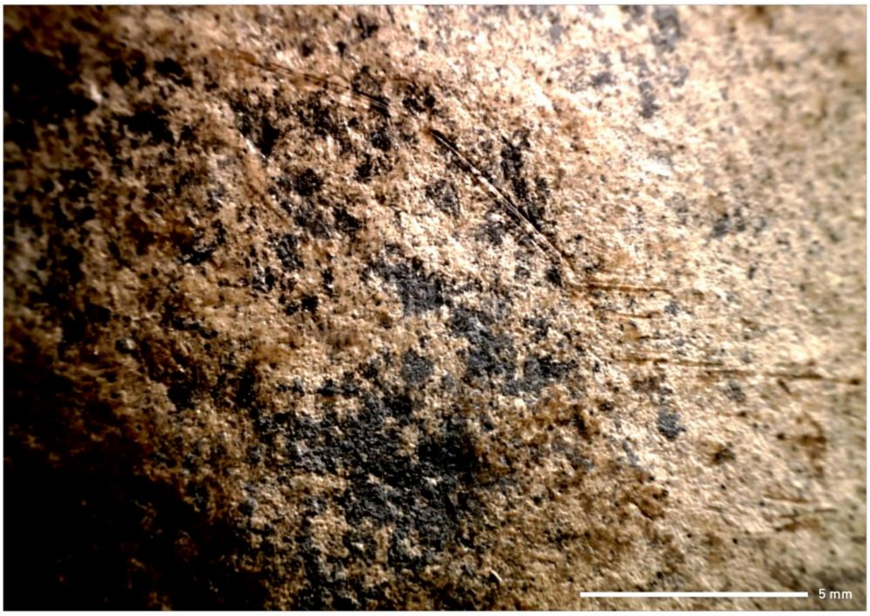

S11J1

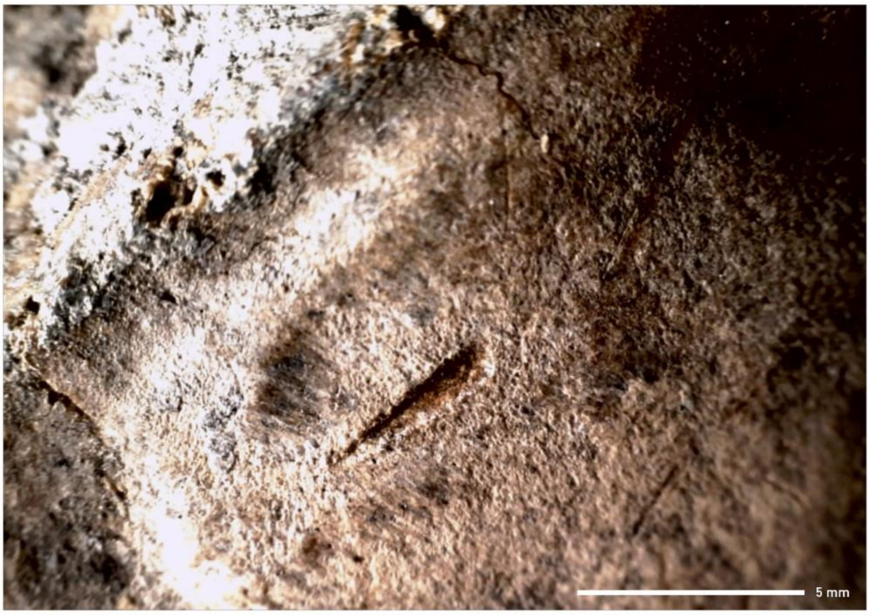

S11J2

Fig. S11J
Bone complex E10, Individual E10 A. Cut marks on *Os carpale III* sin. – S11J1: medial/proximal aspect, S11J: medial aspect. For a detailed description see Table S9.

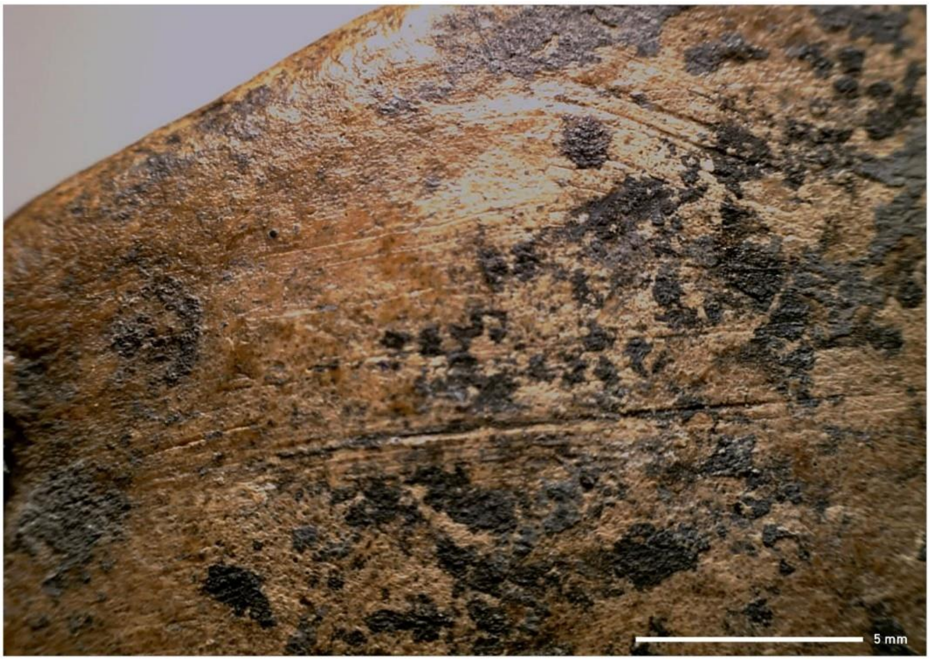

S11K1

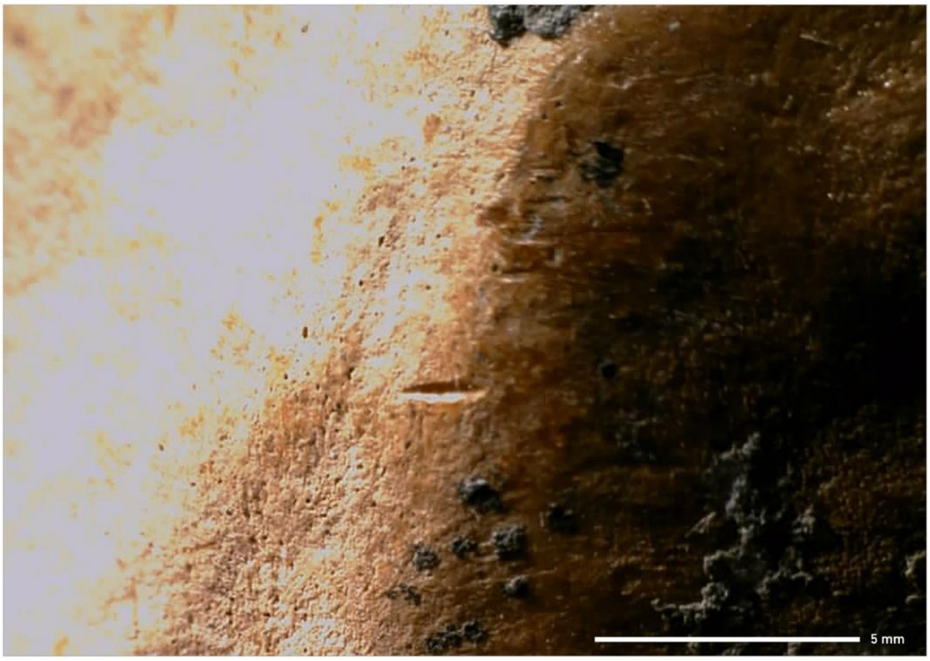

S11K2

Fig. S11K
Bone complex E10, Individual E10 A. Cut marks on *Os carpale IV* dex. – S11K1: proximal aspect, S11K2: proximal/medial aspect. For a detailed description see Table S9.

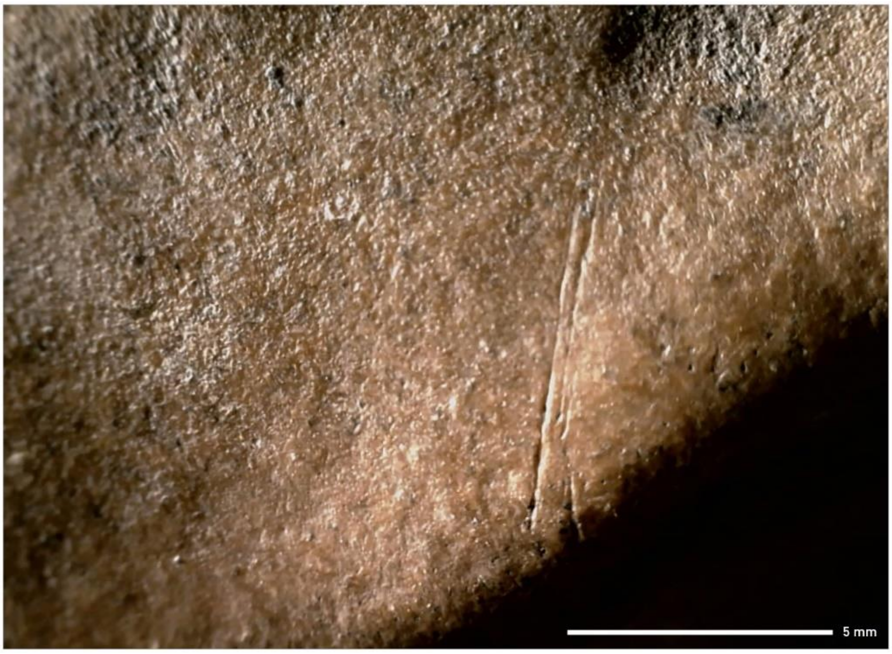

Fig. S11L
Bone complex E10, Individual E10 A. Cut marks on *Os metacarpale III* dex. - proximal aspect. For a detailed description see Table S9.

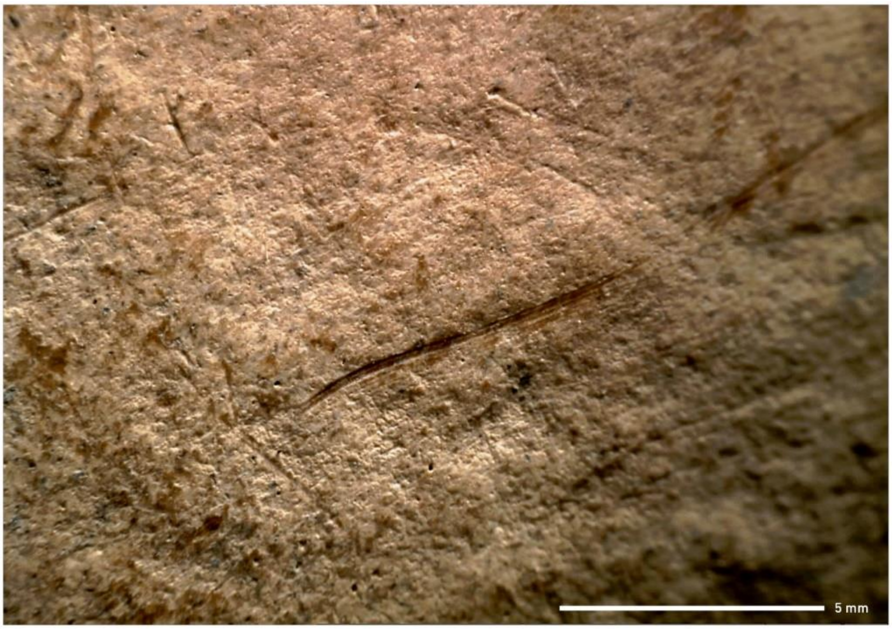

Fig. S11M
Bone complex E10, Individual E10 A. Cut marks on *Os metacarpale IV* sin. – distal/dorsal aspect. For a detailed description see Table S9.

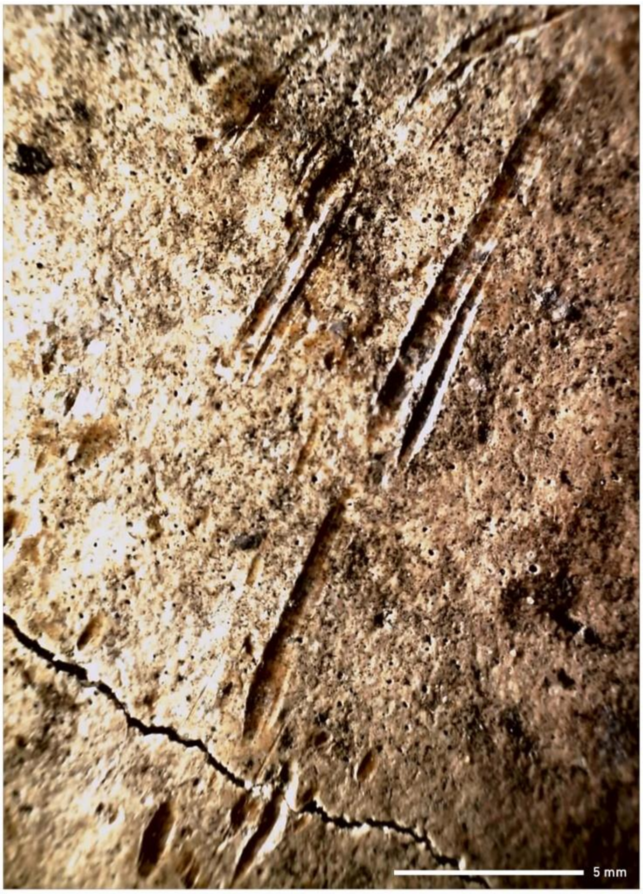

S11N1

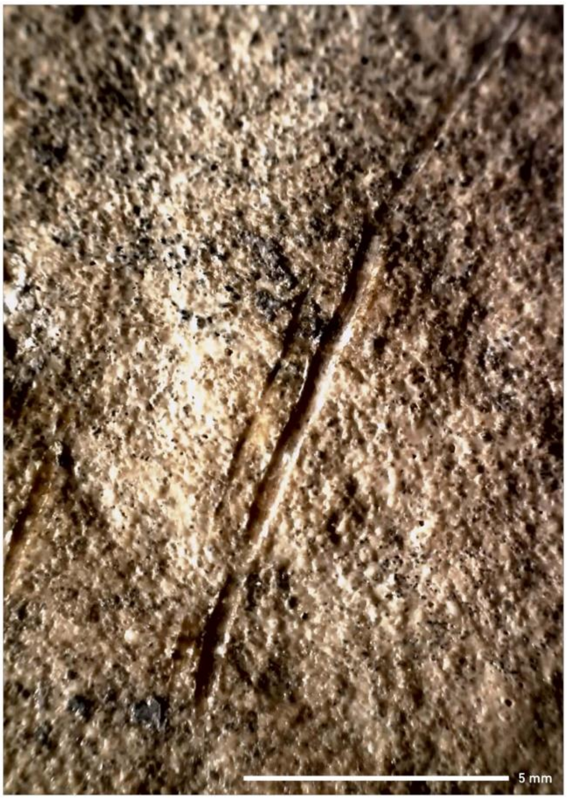

S11N2

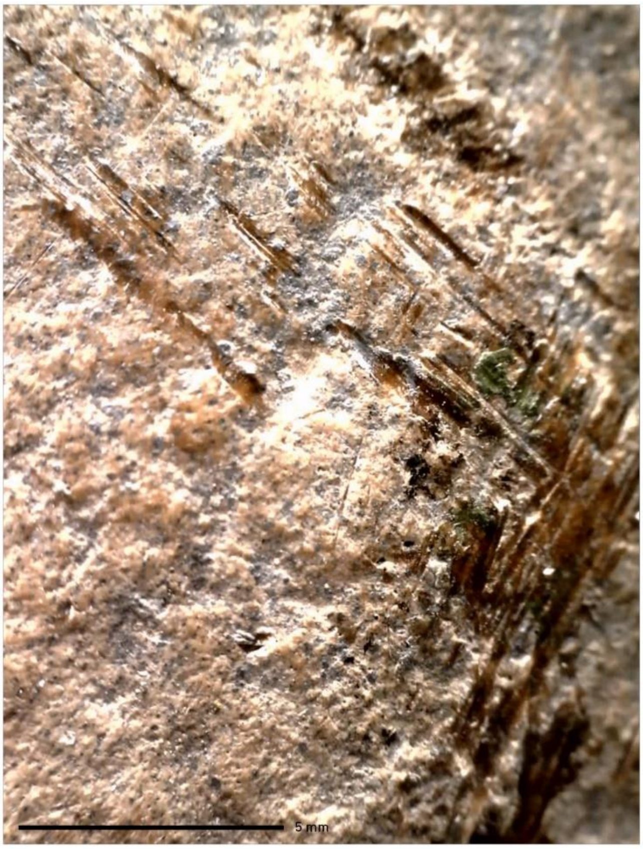

S11N3

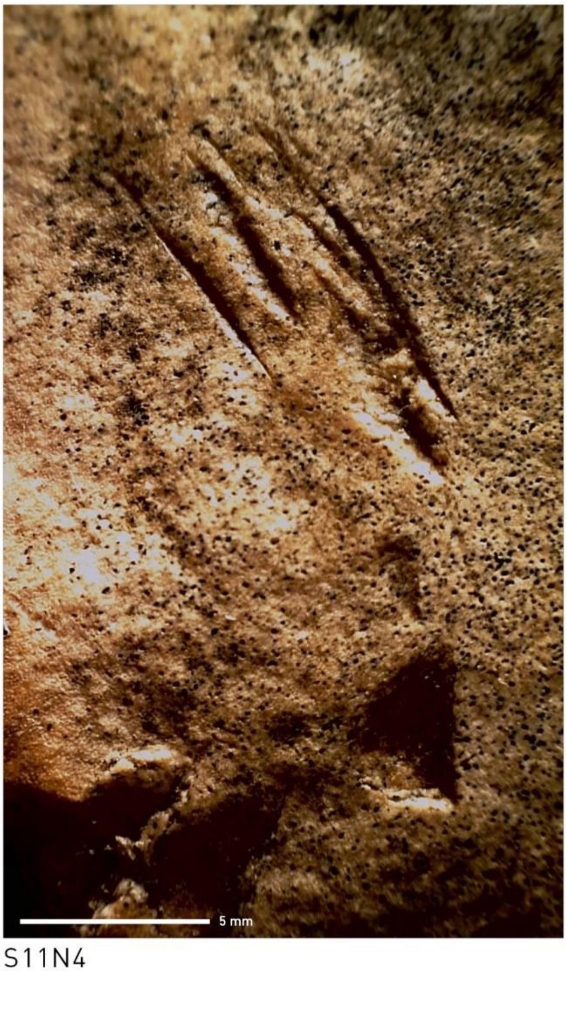

S11N4

Fig. S11N

Bone complex E10, Individual E10 A. Cut marks on *Femur* sin. – S11N1 - S11N2: *Condylus medialis* – distal aspect, S11N3: *Condylus medialis* – caudal aspect, S11N4: *Epicondylus lateralis* – distal / medial aspect. For a detailed description see Table S9.

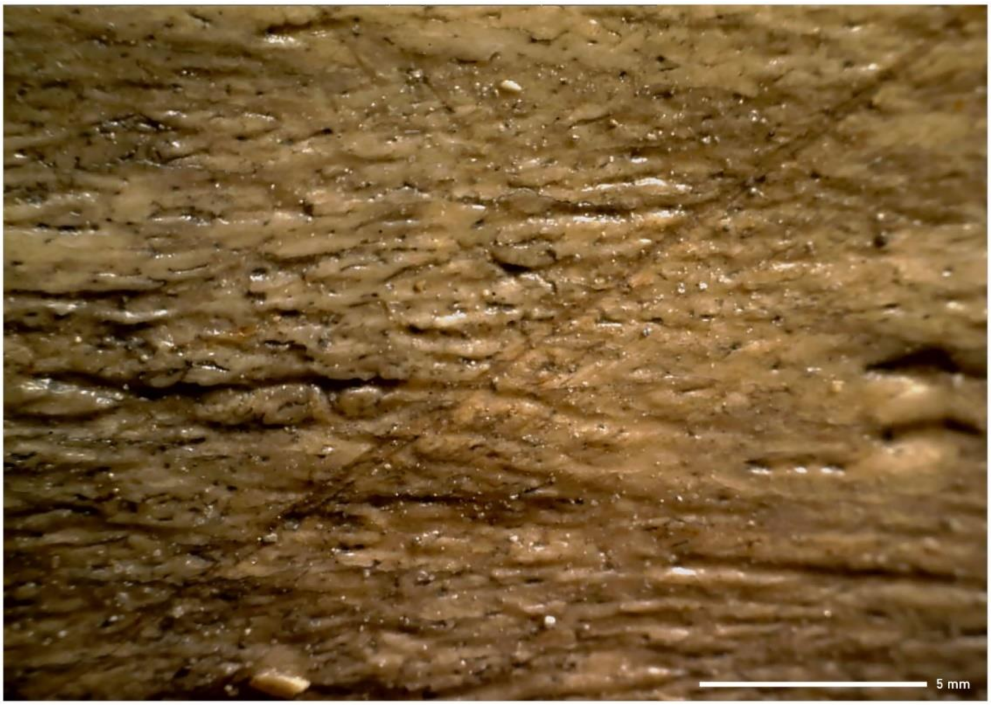

Fig. S11O
Bone complex E10, Individual E10 A. Cut mark on *Tibia* dex. – prox. Diaphysis – cranial aspect. For a detailed description see Table S9.

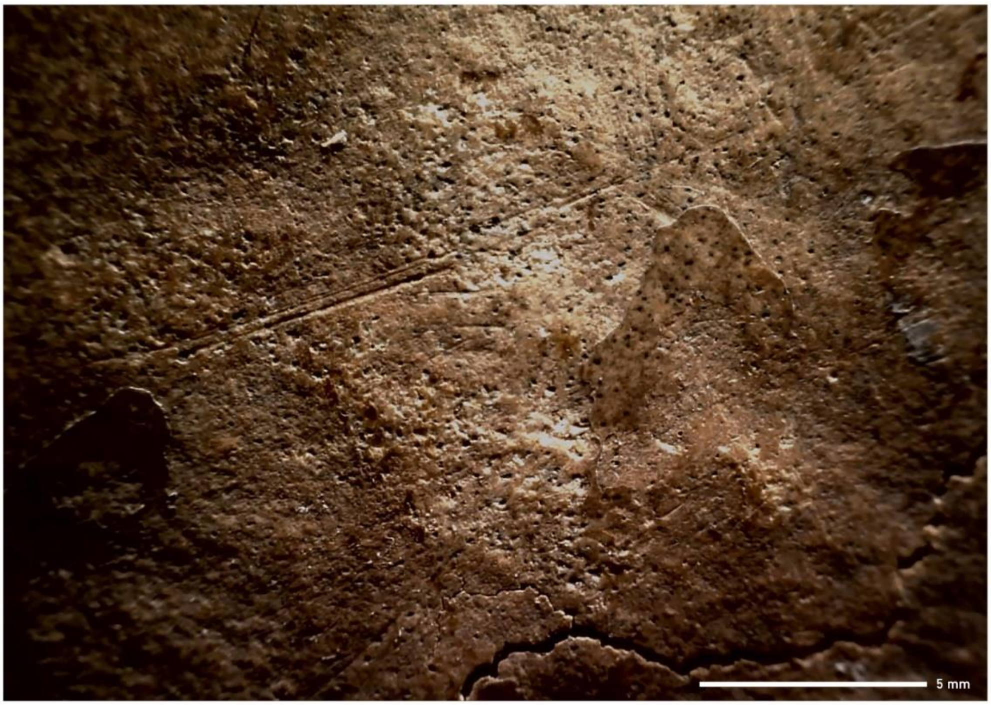

Fig. S11P
Bone complex E10, Individual E10 A. Cut marks on *Talus* dex. – cranial aspect. For a detailed description see Table S9.

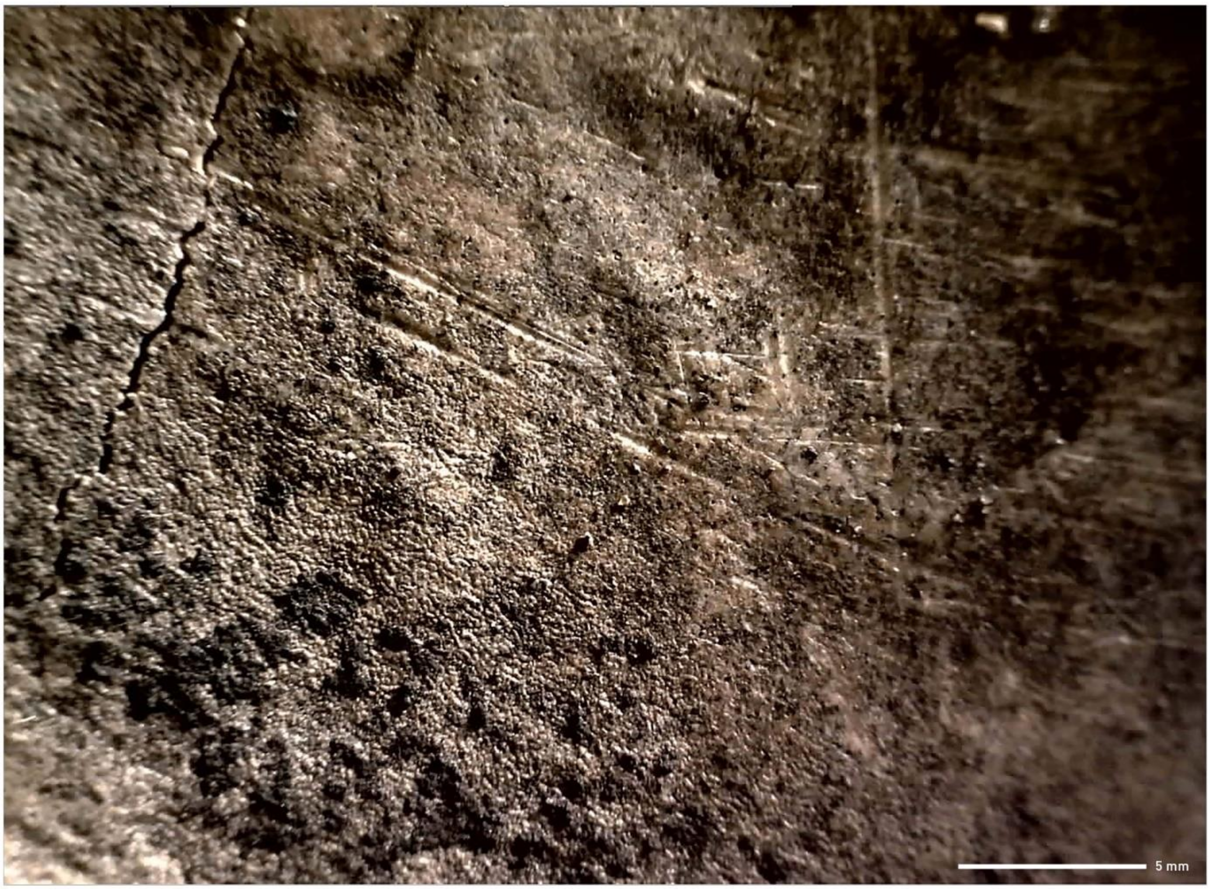

Fig. S12
Bone complex E10, Individual E10 B. Cut marks on *Pelvis* dex. – *Acetabulum*, cranial aspect. For a detailed description see Table S9.

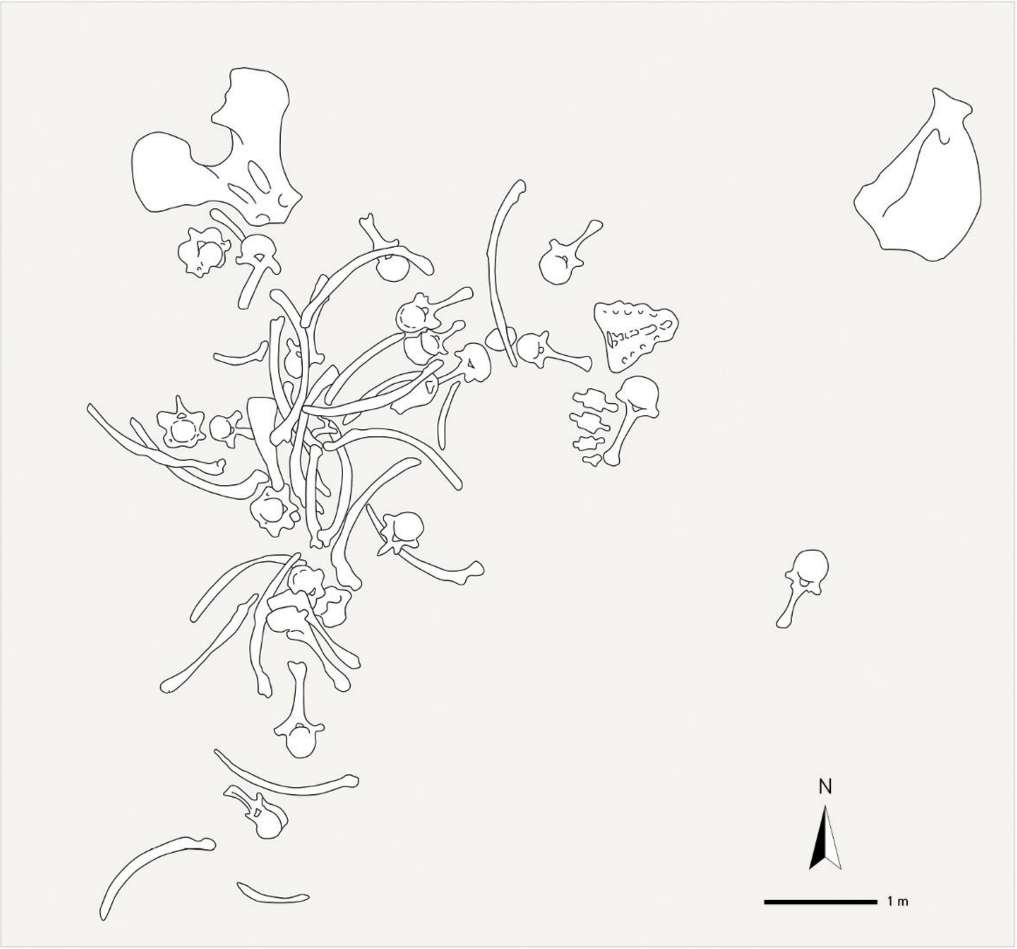

Fig. S13
Find-situation documented for E23. (© Landesamt für Denkmalpflege und Archäologie Sachsen-Anhalt, Dietrich Mania)

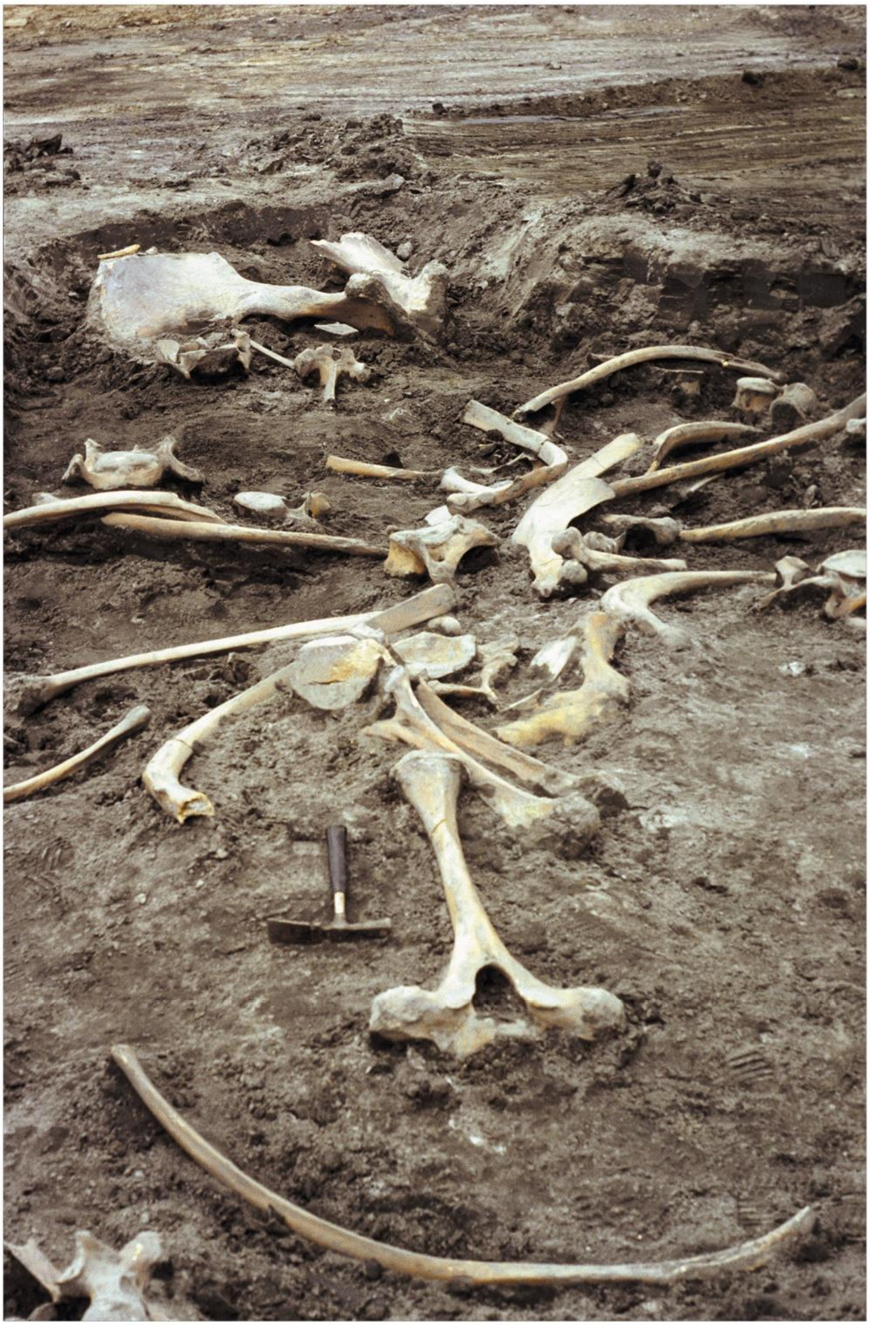

Fig. S14
Find-situation documented for E23, view from the South. (© Landesamt für Denkmalpflege und Archäologie Sachsen-Anhalt, Photo Dietrich Mania)

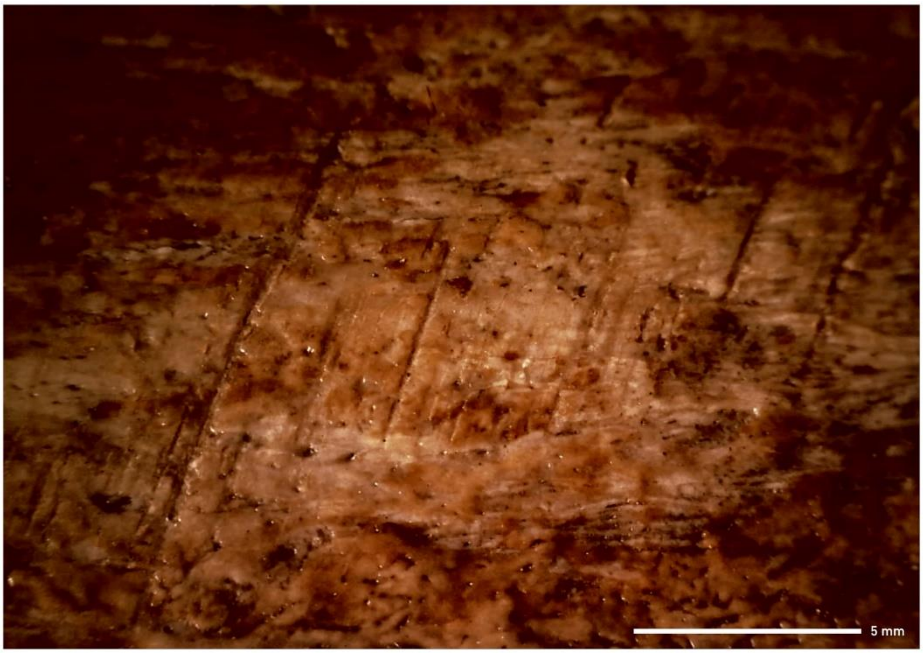

Fig. S15A
Bone complex E23. Cut marks on 1st rib sin. – *Corpus costae*, mid-shaft - medial aspect. For a detailed description see Table S12.

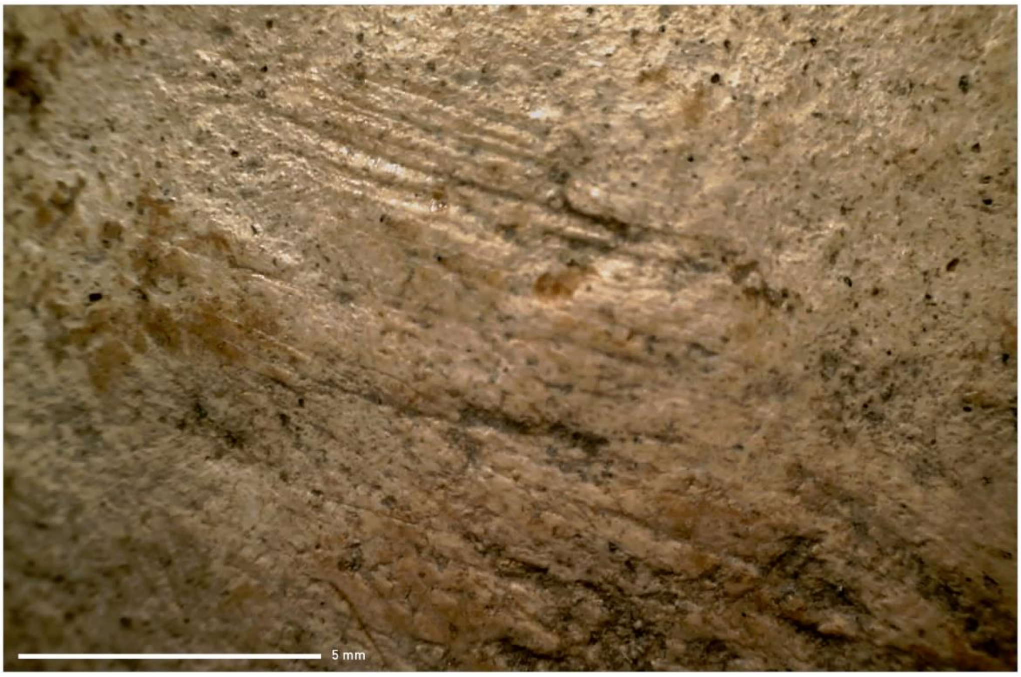

Fig. S15B
Bone complex E23. Cut marks on 4th rib sin. – *Corpus costae*, mid-shaft - medial /distal aspect. For a detailed description see Table S12.

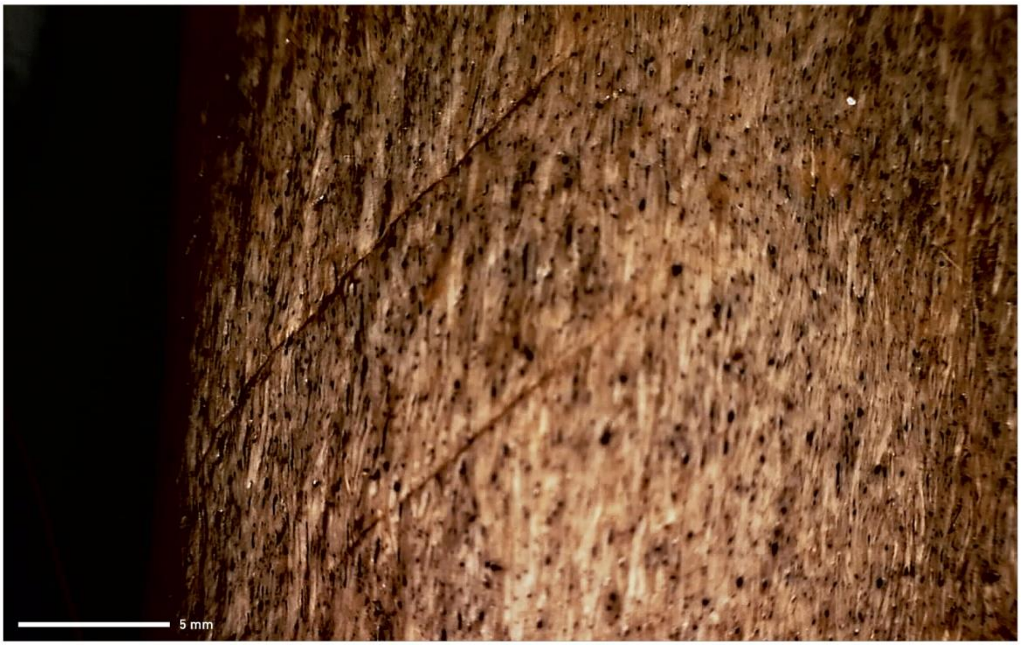

Fig. S15C
Bone complex E23. Cut marks on 17th rib sin. – *Corpus costae*, proximal - caudal / medial aspect. For a detailed description see Table S12.

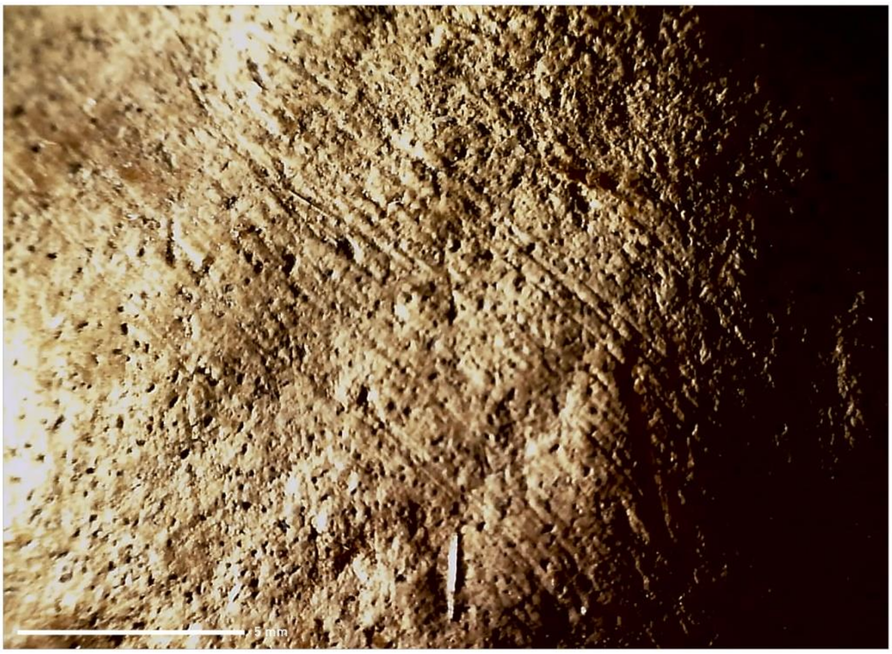

Fig. S15D
Bone complex E23. Cut marks on 8th rib sin. - *Caput costae* - medial aspect. For a detailed description see Table S12.

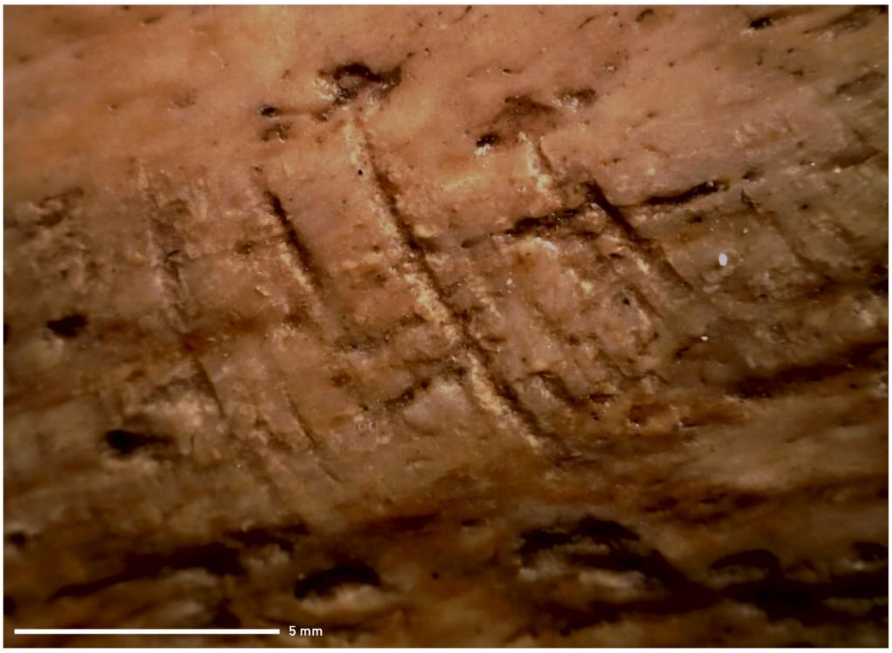

S15E1

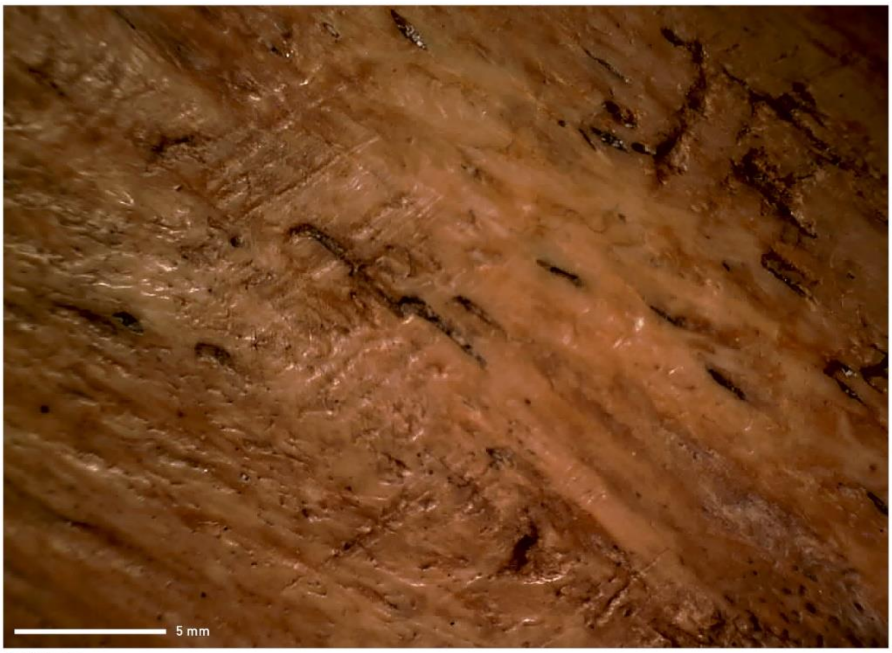

S15E2

Fig. S15E
Bone complex E23. Cut marks on 9th rib sin. – *Corpus costae* – S15E1: cranial aspect, S15E2: medial aspect. For a detailed description see Table S12.

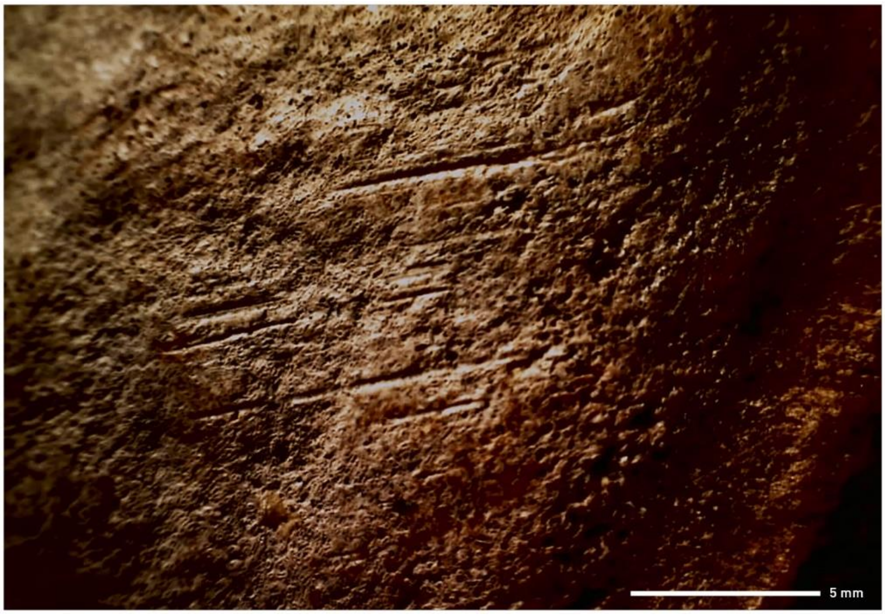

Fig. S15F
Bone complex E23. Cut marks on 4th rib dex. – *Corpus costae*. For a detailed description see Table S12.

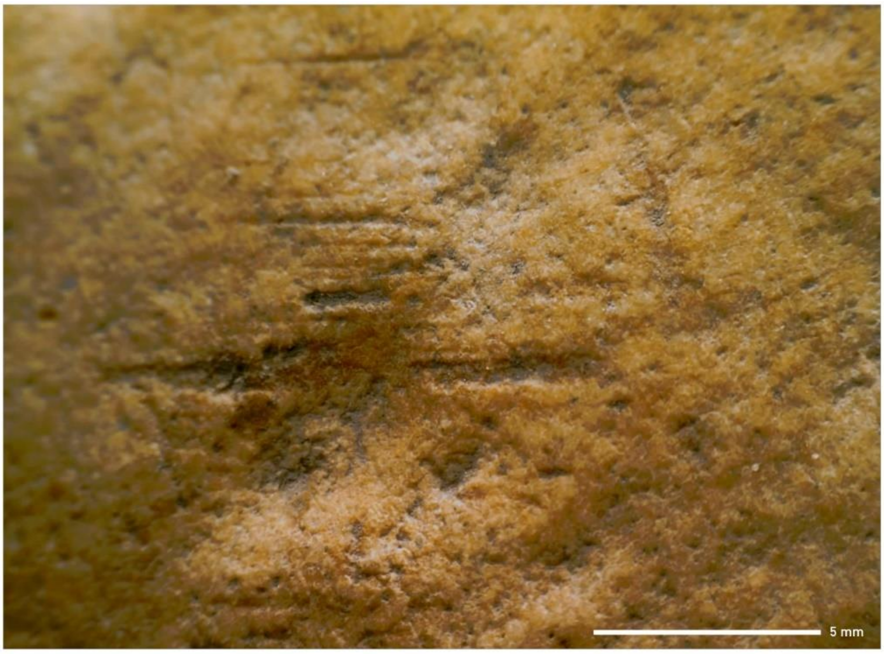

S15G1

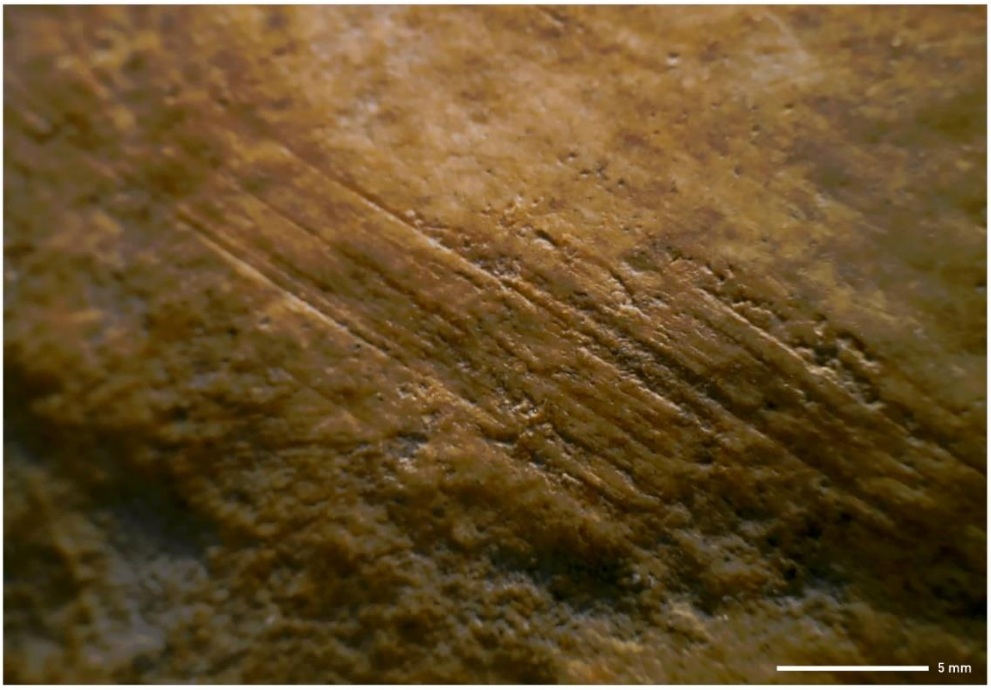

S15G2

Fig. S15G
Bone complex E23. Cut marks on *Humerus* dex. – proximal - dorsal aspect. For a detailed description see Table S12.

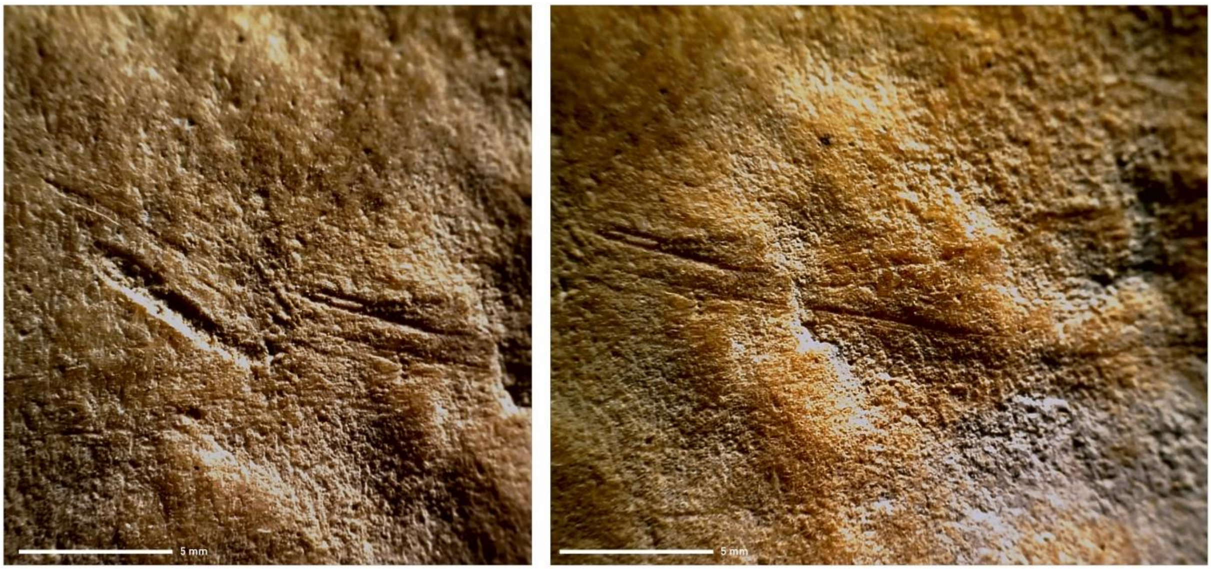

Fig. S15H
Bone complex E23. Cut marks on *Radius / Ulna* sin. – distal aspect. For a detailed description see Table S12.

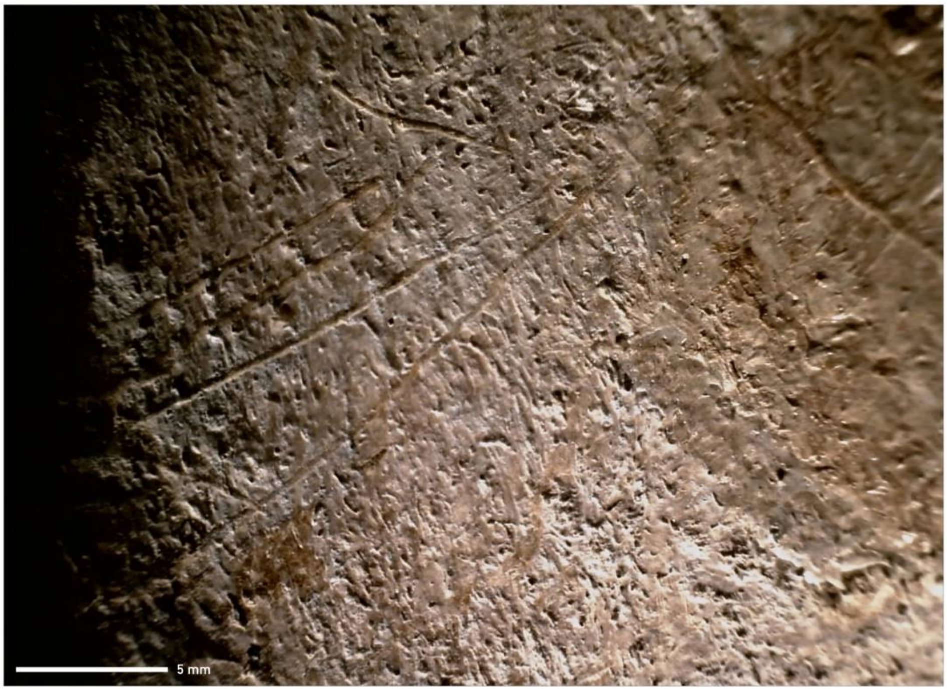

Fig. S15I
Bone complex E23. Cut marks on *Os sacrum* - lateral aspect. For a detailed description see Table S12.

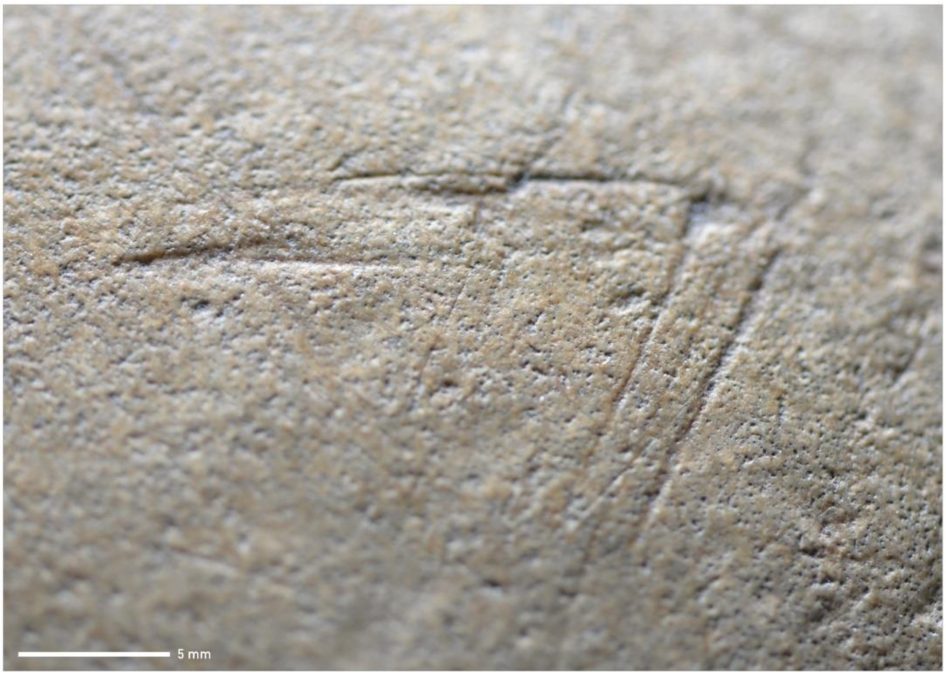

S15J1

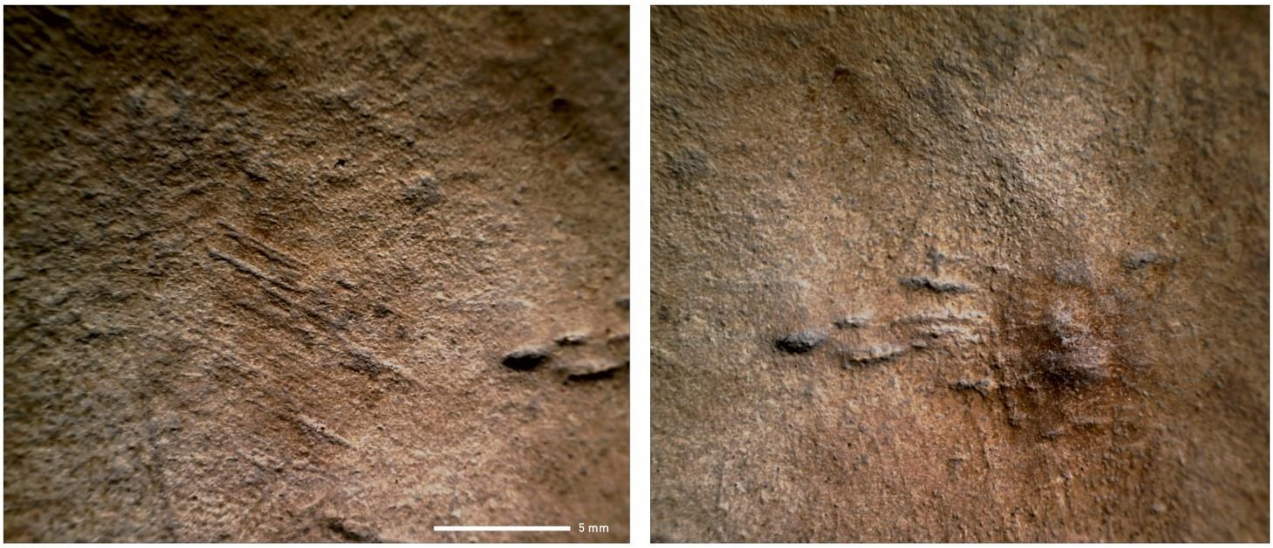

S15J2

Fig. S15J

Bone complex E23. Cut marks on *Femur* dex. – proximal – S15J1: lateral, S15J2: cranial. For a detailed description see Table S12.

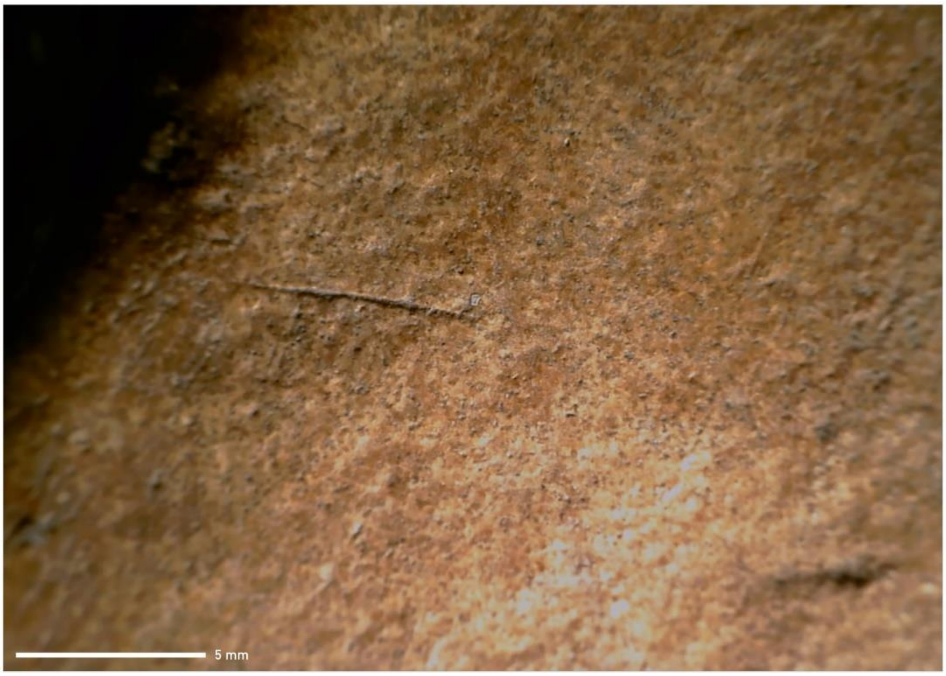

Fig. S15K
Bone complex E23. Cut mark on *Femur* dex. – distal - caudal aspect. For a detailed description see Table S12.

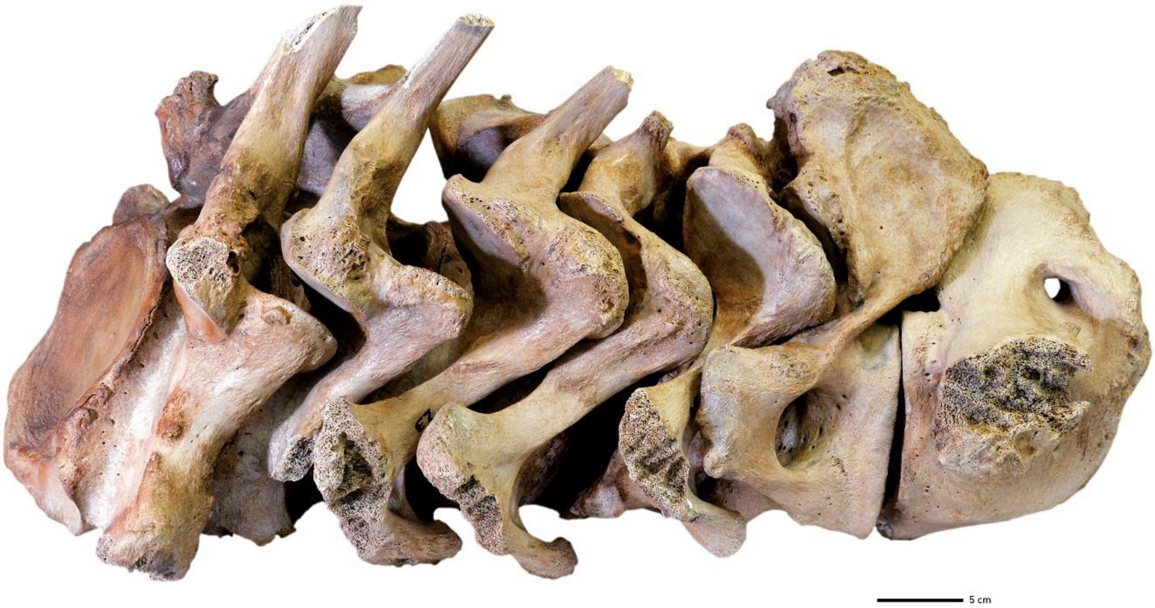

Fig. S16
Bone complex E23, individual E23A. Cervical spine with traces of carnivore modification.

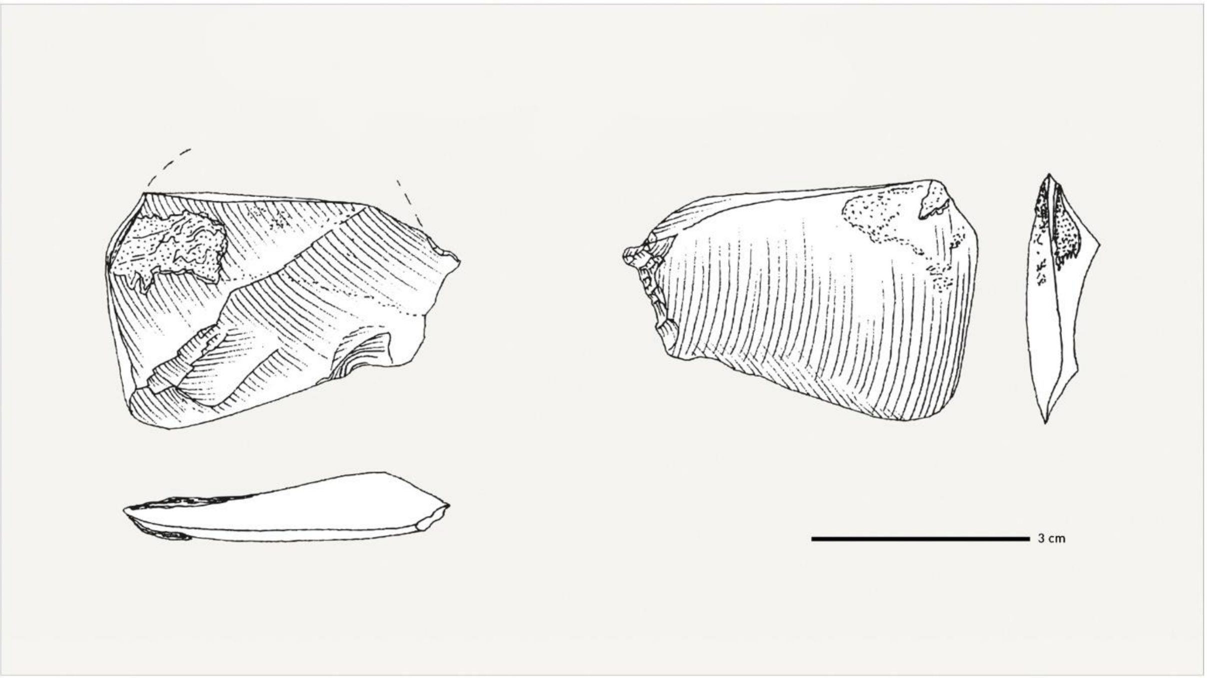

Fig. S17
Flint flake with remains of crushed oak bark on its dorsal and ventral face. (© Landesamt für Denkmalpflege und Archäologie Sachsen-Anhalt, Dietrich Mania)

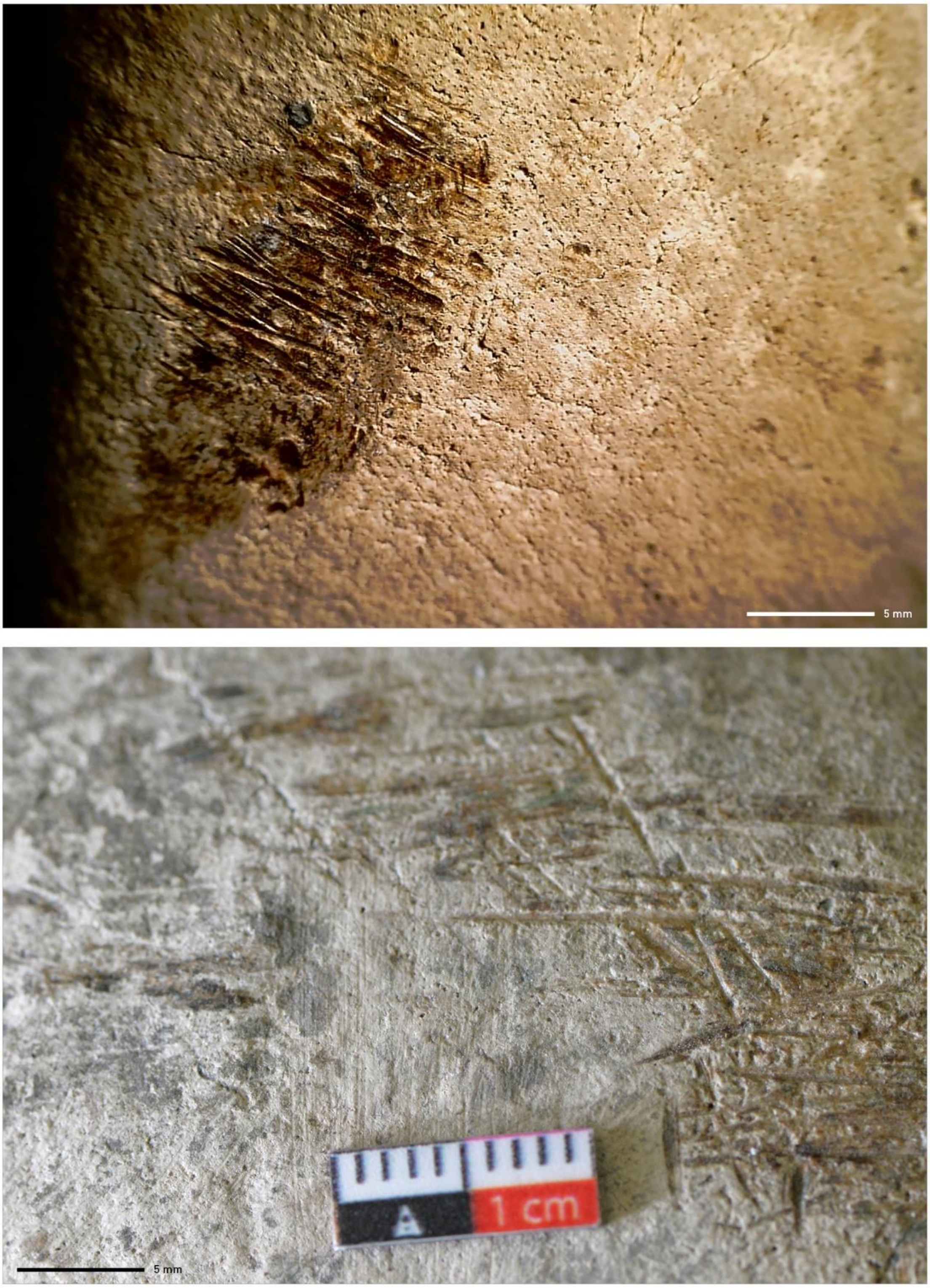

Fig. S18A
Bone complex E30. Cut marks on left (above) and right (below) *Condylus occipitalis* – distal aspect. For a detailed description see Table S14.

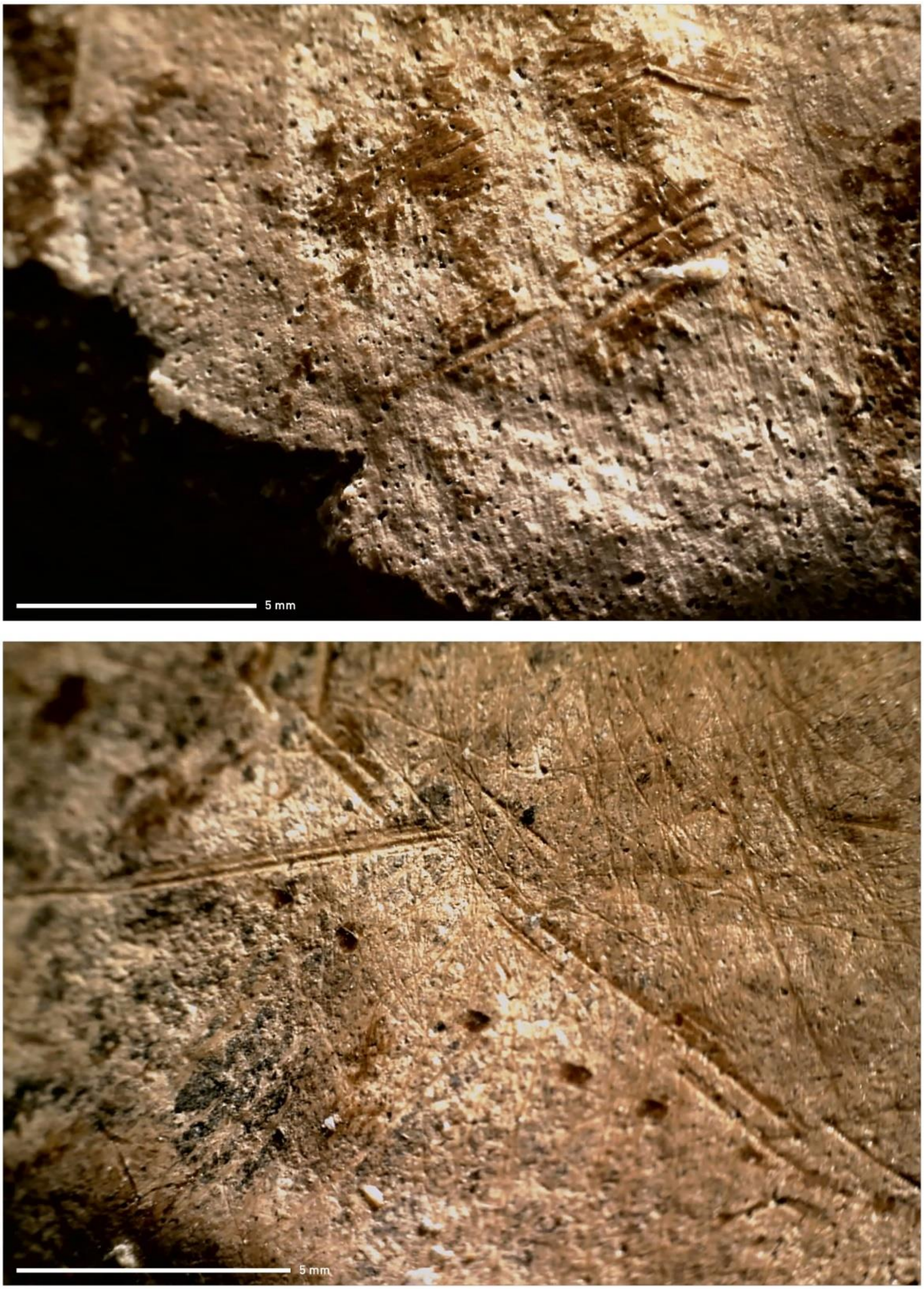

Fig. S18B
Bone complex E30. Cut marks on *Os tarsi centrale* dex. – distal aspect. For a detailed description see Table S14.

Unit	Complex	MNI	Individual	Sex	Age	estimated body mass (in tons)	NISP / MNE	NISP cut / MNE cut	NISP gnaw / MNE gnaw
7	E19	1	E19				15 / 3		
	E30	1	E30				178 / 79	6 / 6	
	E35	2	E35				21 / 13		
	C1	1	C1				2 / 1	1 / 1	
6	E8	1	E8	F	37	3,9	164 / 39	5 / 5	5 / 5
	E9	1	E9	M	47	9,8	74 / 61	9 / 9	4 / 4
	E10	3	E10A	M	47	12,5	223 / 125	44 / 38	7 / 7
			E10B	F	24	5,6			
			E10C						
	E17	1	E17				36 / 4	1 / 1	
	E34	2	E34A	F	39	5,4	14 / 11	2 / 2	3 / 3
			E34B						
	E36	1	E36				62 / 56	4 / 4	5 / 5
	E39	1	E39				31 / 17	1 / 1	4 / 3
6.1	E1	1	E1	M	>39	7,5	38 / 19	4 / 4	
	E7	2	E7A				7 / 6		
			E7B		53				
	E13	1	E13	M	39		4 / 2	1 / 1	
	E21	2	E21A	M			72 / 33	6 / 6	
			E21B	F					
	E22	4	E22A	F	24	5,2	377 / 160	46 / 44	4 / 4
			E22B		<26				
			E22C		<26				
			E22D		<26				
	E23	3	E23A	M	44	9,8	486 / 108	34 / 33	9 / 9
			E23B	M	50	13,0			
			E23C	F	<26	2,0			
	E24	8	E24A	M	50	11,1	854 / 256	50 / 50	10 / 10
			E24B	F	43	5,6			
			E24C	M	43				
			E24D	F	39				
			E24E		30				
			E24F		45				
			E24G	F					
			E24H		53				
	E25	4	E25A	F			11 / 7		
			E25B						
			E25C						
			E25D						
	E26	1	E26		47	13,0	53 / 20	2 / 2	1 / 1
	E29	2	E29A			13,3	37 / 5	2 / 2	1 / 1
			E29B						
	E31	2	E31A				17 / 11		
			E31B						
	E32	1	E32		26	4,0	9 / 5	1 / 1	
	E33	1	E33				12 / 5	2 / 2	
	E40	1	E40	F	36		8 / 2	2 / 2	
	E43	3	E43A	M	47	11,6	223 / 87	22 / 21	8 / 8
			E43B	M		6,8			
			E43C	M					
	C2	2	C2				30 / 10	3 / 3	
4	E5	1	E5				1 / 1		
	E6	1	E6				41 / 20	5 / 5	
unstratified	C3	2	C3				22 / 15	2 / 2	
**Sum**		**57**					**3122 / 1181**	**256 / 245**	**61 / 60**

Table S1

P. antiquus individuals from Neumark-Nord 1 studied for bone surface modifications. Data for composition of bone complexes for Unit 6 and 6.1 and sex, age and body mass taken from (*1*, *23*, *24*). C1-C3 were not provided with designations for bone complexes. MNI - Minimum number of individuals, NISP - Number of identified specimens per taxon, MNE - Minimum Number of Elements, NISPcut / MNEcut - Number of identified specimen per taxon with cut marks / Minimum number of cut marked elements, NISPgnaw / MNEgnaw - Number of identified specimens per taxon modified by large carnivores / Minimum number of elements modified by large carnivores.

Elements	NISP / MNE	NISP cut / MNE cut	NISP gnaw / MNE gnaw	% MNE cut	% MNE gnaw
Cranium (incl. Molars)	633/23	4/4	2/2	17,39 %	8,70 %
Mandible (incl. Molars)	65/23	8/8		34,78 %	0,00 %
isolated Molars	32/17	2/2		11,76 %	0,00 %
Tusk	56/18			0,00 %	0,00 %
Os hyoideum	7/7	2/2		28,57 %	0,00 %
Vert. cervical	57/54	6/6	5/5	11,11 %	9,26 %
Vert. indet	276/71	2/2		2,82 %	0,00 %
Vert. thoracic	126/114	35/35	20/20	30,70 %	17,54 %
Vert tho/lumb	5/3			0,00 %	0,00 %
Vert. lumbar	25/23	4/4	4/4	17,39 %	17,39 %
Vert. caudal	52/52	1/1	1/1	1,92 %	1,92 %
Os sacrum	13/11	2/2		18,18 %	0,00 %
Costae	382/275	54/54	4/4	19,64 %	1,45 %
Os sternum	12/5			0,00 %	0,00 %
Scapula	98/27	7/7	1/1	25,93 %	3,70 %
Humerus	29/19	8/8	3/3	42,11 %	15,79 %
Radius	23/17	6/6	1/1	35,29 %	5,88 %
Ulna	27/22	8/7	3/3	31,82 %	13,64 %
Carpalia	85/85	36/36	1/1	42,35 %	1,18 %
Metacarpus	50/50	13/13	7/7	26,00 %	14,00 %
Phal. prox.	39/39	2/2	1/1	5,13 %	2,56 %
Phal. med.	10/10	2/2		20,00 %	0,00 %
Phal. dist.	2/2			0,00 %	0,00 %
Pelvis	126/27	10/5	3/3	18,52 %	11,11 %
Femur	44/31	20/16	1/1	51,61 %	3,23 %
Tibia	27/22	7/7	2/2	31,82 %	9,09 %
Fibula	12/10			0,00 %	0,00 %
Tarsalia	46/46	13/13	1/1	28,26 %	2,17 %
Metatarsus	29/29	3/3		10,34 %	0,00 %
Patella	8/8	1/1		12,50 %	0,00 %
other Sesamoids	43/39			0,00 %	0,00 %
indet. long bone	70/1			0,00 %	0,00 %
indet. flat bone	57/0			0,00 %	0,00 %
indet. frag	556/1			0,00 %	0,00 %
**Sum**	**3122/1181**	**256/245**	**60/60**	**20,75 %**	**5,08 %**

Table S2

The studied sample, listed according to skeletal element. NISP - Number of identified specimens per taxon, MNE - Minimum Number of Elements, NISPcut / MNEcut - Number of identified specimens per taxon with cut marks / Minimum number of cut marked elements, NISPgnaw / MNEgnaw - Number of identified specimens per taxon modified by large carnivores / Minimum number of elements modified by large carnivores.

Complex	Number of Individuals	Carnivore modification on rump	Carnivore modification on other body parts
Unit 6			
E8	1	Vert. thoracic and caudal (n = 5)	
E9	1	Vert. lumbar (n = 2), *Scapula* (n = 1)	*Os metacarpale* II sin. (n = 1)
E10	3	Vert. thoracic (n = 2), Rib (n = 1)	*Tibia* dex. (n = 1), *Os metacarpale* V dex. (n = 1), *Os metacarpale* II sin. (n = 1), *Phalange proximale* indet. (n = 1)
E34	2		*Humerus* dex. (n = 1), *Radius* indet. (n = 1), *Ulna* sin. (n = 1)
E36	1	Vert. thoracic (n = 5)	
E39	1	Cranium (n = 2), *Pelvis* (n = 1)	*Humerus* sin. (n = 1)
Unit 6.1			
E22	4	Vert. thoracic, lumbar, caudal (n = 3)	*Humerus* sin. (n = 1)
E23	3	Vert. cervical (n = 5), Ribs (n = 3), Cranium (n = 1)	
E24	8	Vert. thoracic (n = 8)	*Femur* sin. (n = 1), *Ulna* dex. (n = 1)
E26	1	*Pelvis* (n = 2)	
E29	2	*Pelvis* (n = 1)	
E43	3		*Tibia* dex. (n = 1), *Ulna* sin. (n = 1), *Os carpi ulnare* sin. (n = 1), *Os metacarpale* I-IV sin. (n = 4), *Os tarsale* III dex. (n = 1)

Table S3
Modification by large carnivores on bones of *P. antiquus* from Neumark-Nord 1 (NISP =61).

Elements	NISP	MNE	NISP cut	MNE cut
Cranium (incl. Molars)				
Mandible (incl. Molars)	1	1		
isolated Molars				
Tusk				
Vert. cervical				
Vert. indet	1	1		
Vert. thoracic				
Vert tho/lumb				
Vert. lumbar				
Vert. caudal				
Os sacrum				
Costae	2	1		
Scapula	1	1		
Humerus	4	1		
Radius	1	1		
Ulna	1	1	1	1
Carpalia	4	4	2	2
Metacarpus	2	2	1	1
Phal. prox.				
Phal. med.				
Phal. dist.				
Pelvis	2	1	1	1
Femur	2	1		
Tibia	2	1		
Fibula				
Tarsalia				
Metatarsus	1	1		
Patella	1	1		
other Sesamoids	1	1		
indet. long bone	11			
indet. flat bone	1			
indet. frag				
**Sum**	**38**	**19**	**5**	**5**

Table S4
Number of identified specimens per taxon (NISP), Number of Elements (MNE) and Number of identified specimens per taxon with cut marks (NISP cut) and Number of Elements with cut marks (MNE cut) for bone complex E6.

Bone	Location of cut marks	Function of cut marks	Figure
Ulna sin.	proximal, cranial, below *Processus cononoideus lateralis*	severing the connection between distal *Radius* and *Ulna* – disarticulation of the left foreleg	Figure S4A
Pelvis sin.	on *Ramus cranialis ossis pubis*	separating the connection between the *Pelvis* and the proximal *Femur* – deboning the left hindleg	
Os carpi intermedium sin.	proximal	severing the connection to left distal *Radius* and *Ulna* – separating the connection between left zeugo- and autopodium	Figure S4B
Os carpi intermedium sin.	distal	severing the connection to the left *Os carpale III* – disarticulation of the left forefoot	
Os carpale II dex.	lateral	severing the connection to the right *Os carpale III* – disarticulation of the right forefoot	
Os carpale II dex.	medial	severing the connenction to *Os carpale I* – disarticulation of the right forefoot	
Os metacarpale III dex.	proximal, medial	severing the connection to the right *Os carpale IV* – disarticulation of the right forefoot	
Os metacarpale III dex.	distal on *Facies articularis*	separating the connection to *Phalange proximale, dig. III* – disarticulation of the right forefoot	

Table S5
Cut marks and sequences of disarticulation observed for bone complex E6.

Elements	NISP	MNE	NISP cut	MNE cut
Cranium (incl. Molars)	11	1		
Mandible (incl. Molars)	1	1		
isolated Molars				
Tusk				
Vert. cervical	5	5		
Vert. indet				
Vert. thoracic	1	1		
Vert tho/lumb				
Vert. lumbar	4	4		
Vert. caudal	2	2		
Os sacrum	1	1		
Costae				
Scapula	2	2	2	2
Humerus	2	2		
Radius	2	2	1	1
Ulna	2	2	1	1
Carpalia	14	14	2	2
Metacarpus	10	10	1	1
Phal. prox.	4	4		
Phal. med.				
Phal. dist.				
Pelvis	1	1		
Femur	2	2	2	2
Tibia	1	1		
Fibula	1	1		
Tarsalia	3	3	2	2
Metatarsus				
Patella	1	1		
other Sesamoids	1	1		
indet. long bone	3	1		
indet. flat bone				
indet. frag	1	1		
**Sum**	**74**	**62**	**11**	**11**

Table S6
Number of identified specimens per taxon (NISP), Number of Elements (MNE) and Number of identified specimens per taxon with cut marks (NISP cut) and Number of Elements with cut marks (MNE cut) for bone complex E9.

**Bone**	**Location of cut marks**	**Function of cut marks**	**Figure**
Ulna sin.	proximal, cranial, below *Processus anconeus*	separating the connection between distal *Humerus* and *Ulna* – severing the connection between the left stylo- and zeugopodium	Figure S7A
Femur dex.	proximal, cranial and lateral on *Caput ossis femoris*	separating the connection between the proximal *Femur* and the *Acetabulum* – deboning the right hindleg	
Femur dex.	distal on *Epicondylus lateralis* and *medialis*	separating the connection between distal *Femur* and proximal *Tibia* – severing the connection between right stylo- and zeugopodium	Figure S7B
Femur sin.	distal, caudal on *Epicondylus lateralis*	separating the connection between distal *Femur* and proximal *Tibia* – severing the connection between left stylo- and zeugopodium	Figure S7C
Talus dex.	cranial, on *Trochlea tali*	severing the connection to the distal *Tibia* – separating the connection between right zeugo- and autopodium	Figure S7D
Calcaneus sin.	on *Facies articulares talares*	disarticulation of the left foot – separating the connection between the left zeugo- and autopodium	
Os carpi intermedium dex.	medial	severing the connection to the right *Os carpi radiale* – disarticulation of the right forefoot	
Os carpale IV dex.	distal	severing the connection to the right *Os metacarpus III* – disarticulation of the right forefoot	Figure S7E
Phalange proximale sin.	dorsal, proximal	severing the connection to the left distal metapodials – disarticulation of a left forefoot or hindfoot	Figure S7F

Table S7
Cut marks and sequences of disarticulation observed for bone complex E9.

Elements	NISP	MNE	NISP cut	MNE cut
Cranium (incl. Molars)				
Mandible (incl. Molars)				
isolated Molars				
Tusk				
Vert. cervical	9	8		
Vert. indet	48			
Vert. thoracic	16	14	5	4
Vert tho/lumb				
Vert. lumbar	2	2	2	2
Vert. caudal	3	3		
Os sacrum	1	1		
Costae	29	25	13	11
Scapula	1	1	1	1
Humerus	4	3	3	2
Radius	2	2	1	1
Ulna	3	3	2	1
Carpalia	14	14	7	7
Metacarpus	9	9	2	2
Phal. prox.	8	8		
Phal. med.	4	4		
Phal. dist.	1	1		
Pelvis	9	3	7	3
Femur	2	2	1	1
Tibia	4	3	2	1
Fibula	4	2		
Tarsalia	4	4	1	1
Metatarsus	4	4	1	1
Patella	1	1		
other Sesamoids	9	9		
indet. long bone	13			
indet. flat bone	19			
indet. frag				
**Sum**	**223**	**125**	**48**	**38**

Table S8
Number of identified specimens per taxon (NISP), Number of Elements (MNE) and Number of identified specimens per taxon with cut marks (NISP cut) and Number of Elements with cut marks (MNE cut) for bone complex E10.

Bone	Location of cut marks	Function of cut marks	Figure
Vertebrae – 12th/13th and 19th thoracal and 1st-2nd lumbar (n=5)	right lateral, *Processus spinosus*, medial/proximal	removal of tenderloin – defleshing, while carcass rested on its left side	Figure S11A
Ribs sin.	cranial, lateral *Corpus costae* (6th rib – lateral towards cranial mid-shaft; 7th rib – lateral towards cranial mid-shaft, 16th rib – lateral, proximal; Rib sin. – cranial, mid-shaft)	removal of skin, fat and connective tissue while carcass rested on its right side	Figure S11B
Ribs dex.	caudal, medial *Corpus costae* (14th rib – medial, mid-shaft; 15th rib – medial, mid-shaft; 16th rib – medial, mid-shaft; 18th rib – medial, mid-shaft; 2x Rib indet. dex. – medial mid-shaft)	removal of fat and connective tissue while carcass rested on its right side	
Pelvis indet.	*Os ilium*, lateral	removal of skin and fat – defleshing of carcass	
Pelvis dex.	*Os ilium*, lateral	removal of skin and fat – defleshing of carcass	
Pelvis indet.	*Os ilium*, medial	removal of the viscera – evisceration of the carcass	
Pelvis dex.	*Os ilium*, medial	removal of the viscera – evisceration of the carcass	Figure S11C
Pelvis sin.	caudal, *Ramus cranialis ossis pubis*	removal of the viscera – evisceration of the carcass	
Scapula dex.	medial, on *Collum scapulae*	detachment of the *Scapula* – dissection of the right half of the torso	Figure S11D
Scapula dex.	lateral, on *Fossa supraspinata*	removal of muscle mass – defleshing of the right half of the torso	
Humerus sin.	distal and cranial on *Trochlea humeri*	separating of the joint-connection to the proximal *Ulna* – disarticulation of the foreleg – separating the connection between left stylo- and zeugopodium	
Humerus sin.	cranial on *Tuberositas deltoidea*	meat filleting of left forefoot	
Humerus dex.	distal and cranial on *Trochlea humeri*	separating of the joint-connection to the proximal *Ulna* – disarticulation of the forefoot – separating the connection between right stylo- and zeugo-podium	Figure S11E
Radius/*Ulna* sin.	distal on *Facies articularis carpea* – separating the connection to *Os carpi ulnare*	severing the connection between left auto- and zeugopodium	Figure S11F
Os carpi intermedium sin.	distal	severing the connection to the left *Os carpale III* – disarticulation of the left forefoot	
Os carpi intermedium dex.	proximal	severing the connection to right distal *Radius* and *Ulna* – separating the connection between right zeugo- and autopodium	Figure S11G
Os carpi intermedium sin.	medial	severing the connection to the left *Os carpi radiale* – disarticulation of the left forefoot	Figure S11H
Os carpi ulnare sin.	proximal	severing the connection to the left distal *Ulna* – severing the connection between left auto- and zeugopodium	Figure S11I
Os carpale III sin.	proximal	severing the connection to the left *Os carpi intermedium* – disarticulation of the left forefoot	
Os carpale III sin.	medial	severing the connection to the left *Os carpale II* – disarticulation of the left forefoot	Figure S11J
Os carpale IV dex.	proximal	severing the connection to the right *Os carpi ulnare* – disarticulation of the right forefoot	
Os carpale IV dex.	medial	severing the connection to the right *Os carpale III* – disarticulation of the right forefoot	Figure S11K
Os metacarpale III dex.	proximal	severing the connection to the right *Os carpale III* – disarticulation of the right forefoot	Figure S11L
Os metacarpale III dex.	lateral	severing the connection to the right *Os metacarpale IV* – disarticulation of the right forefoot	
Os metacarpale IV sin.	distal, dorsal	severing the connection to the left *Phalange proximale, dig IV* – disarticulation of the left forefoot	Figure S11M
Femur sin.	*Trochlea ossis femoris, Condylus lateralis, Condylus medialis*, distal	separating the connection between left *Patella* and left proximal *Tibia* – severing the left stylo- and zeugopodium	Figure S11N
Tibia dex.	proximal midshaft lateral, midshaft lateral	meat filleting of right hindleg	
Tibia dex.	midshaft cranial, next to *Margo cranialis*	meat filleting of right hindleg	Figure S11O
Talus dex.	cranial on *Trochlea tali*	severing the connection to the distal *Tibia* – separating the right zeugo- and autopodium	Figure S11P
Os metatarsale IV dex.	proximal	severing the connection to the right *Os tarsale IV* – disarticulation of the right metatarsal	

Table S9
Cut marks and sequences of disarticulation observed for bone complex E10, Individual E10A.

Bone	Location of cut marks	Function of cut marks	Figure
Pelvis dex.	*Acetabulum*, on *Facies lunata* towards *Os ilium*	severing the connection between the *Pelvis* and the proximal *Femur* – deboning the right hindleg	Figure S12

Table S10
Cut marks and sequences of disarticulation observed for bone complex E10, Individual E10B.

Elements	NISP	MNE	NISP cut	MNE cut
Cranium (incl. Molars)	77	2		
Mandible (incl. Molars)	3	2		
isolated Molars	1	1		
Tusk	3	2		
Vert. cervical	9	9		
Vert. indet	4			
Vert. thoracic	18	16	11	11
Vert tho/lumb				
Vert. lumbar	3	3		
Vert. caudal	3	3		
Os sacrum	1	1	1	1
Os sternum	4	1		
Costae	60	38	14	14
Scapula	7	2		
Humerus	2	1	1	1
Radius	2	2	1	1
Ulna				
Carpalia	4	4	3	3
Metacarpus				
Phal. prox.	2	2	1	1
Phal. med.				
Phal. dist.	1	1		
Pelvis	15	2		
Femur	2	1	2	1
Tibia				
Fibula	1	1		
Tarsalia				
Metatarsus	1	1		
Patella				
other Sesamoids	13			
Os hyoideum	1	1		
indet. long bone	2			
indet. flat bone				
indet. frag	247			
**Sum**	**486**	**95**	**34**	**33**

Table S11
Number of identified specimens per taxon (NISP), Number of Elements (MNE) and Number of identified specimens per taxon with cut marks (NISP cut) and Number of Elements with cut marks (MNE cut) for bone complex E23.

Bone	Location of cut marks	Function of cut marks	Figure
Vertebrae	1st thoracal – right lateral, *Processus spinosus* medial/distal; 9th thoracal – left lateral, *Processus spinosus* distal; 11th thoracal – righ/left lateral, *Processus spinosus* distal; 12th thoracal – right lateral, *Processus spinosus* medial and left lateral, *Processus spinosus* proximal; 14th thoracal – left lateral, *Processus spinosus* proximal and distal; 16th thoracal – left lateral, *Processus spinosus* medial	removal of tenderloin – defleshing the carcass	
Vertebrae	4th thoracal – left lateral, *Processus spinosus* above *Processus articularis caudalis*; 6th thoracal – left caudal, *Processus transversus*; 19th thoracal – right lateral, *Processus transversus*	separating ribs from vertebrae – disarticulation of the ribcage	
Vertebrae	2nd thoracal – caudal, *Processus spinosus* distal sin. and cranial on *Processus articularis cranialis* sin.; 18th thoracal – right lateral, *Corpus vertebrae* distal	separation of vertebrae – disarticulation of the spine	
1st Rib sin.	medial, *Corpus costae*, mid-shaft	removal of skin, fat and connective tissue while carcass rested on its left side	Figure S15A
3rd Rib sin.	medial, *Corpus costae*, mid-shaft	removal of skin, fat and connective tissue while carcass rested on its left side	
4th Rib sin.	medial, *Corpus costae*, mid-shaft and distal	removal of skin, fat and connective tissue while carcass rested on its left side	Figure S15B
6th Rib sin.	medial, *Corpus costae*, distal	removal of skin, fat and connective tissue while carcass rested on its left side	
11th Rib sin.	caudal/medial, *Corpus costae*, mid-shaft	removal of skin, fat and connective tissue while carcass rested on its left side	
17th Rib sin.	caudal/medial, *Corpus costae*, proximal	removal of skin, fat and connective tissue while carcass rested on its left side	Figure S15C
8th Rib sin.	caudal, *Caput costae*	separating rib from vertebrae – disarticulation of ribcage	Figure S15D
9th Rib sin.	cranial/lateral, *Corpus costae*, proximal mid-shaft	removal of skin, fat and connective tissue	Figure S15E
4th Rib dex.	Caput costae	separating rib from vertebrae – disarticulation of ribcage	Figure S15F
6th Rib dex.	*Caput costae*	separating rib from vertebrae – disarticulation of ribcage	
5th Rib dex.	caudal/medial, *Corpus costae*, proximal	removal of skin, fat and connective tissue while carcass rested on its right side	
8th Rib dex.	lateral *Corpus costae*, proximal/mid-shaft	removal of skin, fat and connective tissue	
14th Rib dex.	caudal, *Corpus costae*, proximal	removal of skin, fat and connective tissue	
17th Rib dex.	cranial, *Corpus costae*, mid-shaft	removal of skin, fat and connective tissue	
Humerus dex.	proximal/dorsal on *Caput humeri* and *Tuberculum majus, pars caudalis*	separating *Humerus* from *Scapula* – deboning the right foreleg	Figure S15G
Radius/Ulna sin.	distal on *Facies articularis carpea*	separating the connection to *Os carpi ulnare* – severing the connection between left auto- and zeugopodium	Figure S15H
Os sacrum	lateral left on *Christa sacralis mediana*	removal of skin, fat and connective tissue while carcass rested on its left side	Figure S15I
Femur dex.	proximal, cranial and lateral on *Caput ossis femoris*	separating the connection between the proximal *Femur* and the *Acetabulum* – deboning the right hindleg	Figure S15J
Femur dex.	distal, caudal on *Epicondylus lateralis*	separating the connection between distal *Femur* and proximal *Tibia* – severing the connection between right stylo- and zeugopodium	Figure S15K
Os carpi intermedium sin.	distal	severing the connection to the left *Os carpale III* – disarticulation of left forefoot	
Os carpale II sin.	proximal	severing the connection to the left *Os carpi radiale* – disarticulation of left forefoot	
Os carpale II sin.	distal	severing the connection to the left *Os metacarpale II* and *Os carpale I* – disarticulation of the left forefoot	
Os carpale III dex.	distal	severing the connection to the left *Os metacarpale III* – disarticulation of left forefoot	
Phalange proximale, dig. III sin.	proximal	severing the connection to the left distal metapodials – disarticulation of a left forefoot/hindfoot	
Phalange proximale, dig. III sin.	distal	severing the connection to *Phalange mediale, dig. III* sin. – disarticulation of a left forefoot/hindfoot	

Table S12
Cut marks and sequences of disarticulation observed for bone complex E23.

Elements	NISP	MNE	NISP cut	MNE cut
Cranium (incl. Molars)	13	1	1	1
Mandible (incl. Molars)	25	1		
isolated Molars				
Tusk	3	1		
Vert. cervical	7	7		
Vert. indet				
Vert. thoracic	16	16		
Vert tho/lumb	5	3		
Vert. lumbar	1	1		
Vert. caudal	3	3		
Os sacrum				
Costae	24	16	2	1
Scapula	12	1	1	1
Humerus	1	1		
Radius				
Ulna	1	1		
Carpalia	1	1		
Metacarpus	1	1	1	1
Phal. prox.	7	7		
Phal. med.				
Phal. dist.				
Pelvis	12	1		
Femur	1	1		
Tibia	1	1		
Fibula	1	1		
Tarsalia	9	9	1	1
Metatarsus	5	5		
Patella				
other Sesamoids				
indet. long bone	2			
indet. flat bone				
indet. frag	27			
**Sum**	**178**	**79**	**6**	**5**

Table S13
Number of identified specimens per taxon (NISP), Number of Elements (MNE) and Number of identified specimens per taxon with cut marks (NISP cut) and Number of Elements with cut marks (MNE cut) for bone complex E30.

Bone	Location of cut marks	Function of cut marks	Figure
Skull	left and right *Condylus occipitalis*, distal	separating skull from *Atlas* – disarticulation of zono-skeleton	Figure S18A
Scapula	medial on *Fossa subscapularis*	detachment of the *Scapula* – dissection of the right half of the torso	
Rib dex.	caudal on *Caput costae*	separating rib from vertebrae – disarticulation of ribcage	
Rib dex.	caudal medial on *Corpus costae*	removal of fat and connective tissue	
Os metacarpus III sin.	dorsal, distal	separating the connection to the left *Phalange proxi-male, dig. III* – disarticulation of the left forefoot	
Os tarsi centrale dex.	distal	severing the connection to the right *Os metatarsus IV* – disarticulation of the right hindfoot	Figure S18B

Table S14
Cut marks and sequences of disarticulation observed for bone complex E30.

Neumark-Nord 1

PAZ	Duration in years	Lake phase	unit	sediments	Elephant assemblages studied (MNE/MNI)	Associated fauna and archaeology
VII	~ 2,000	*tr*	11-9	peat silts		*B. primigenius* skull and partial skeleton of *C. elaphus*
VI	~ 2,000	*re*	8	reworked silts and gyttja		
		tr	7	gyttja		partial/complete skeletons of *D. dama* (MNI=2) and *C. elaphus* (MNI=1)
V	~ 4,000				E19 (3/1), E30 (79/1), E35 (13/2), C1 (1/1)	partial/complete skeletons of *D. dama* (MNI=104), *C. elaphus* (MNI=22) and *Cervus gen. spec.* indet. (MNI=8) with cut marks and hunting lesions and *B. primigenius* (MNI=2), Some scattered flint artefacts
IVb	~ 1,200		6	silts		
		re	6.2	littoral deposits, formed during <100 years		Skeleton of *St. hemitoechus* with lithics, partial skeleton of *B. primigenius*, isolated bones of *Rhinoceros* sp.(MNI=1), *M. giganteus* (MNI=1) and *C. crocuta* (MNI=1), scatters with lithics (n= 654) and bone fragments (NISP> 300) of *P. antiquus, Rhinoceros* sp., *Equus* sp., *B. primigenius, D. dama*, *C. elaphus* (MNI=7)
IVa	~ 1,200	*tr*	6	silts	E8 (39/1), E9 (61/1), E10 (125/3), E17 (4/1), E34 (11/2), E36 (56/1), E39 (17/1)	partial/complete skeletons of *D. dama* (MNI=23), *C. elaphus* (MNI=4), *B. primigenius* (MNI=2), isolated bones of *P. leo* (MNI=1), *St. kirchbergensis* (MNI=2), *B. primigenius* (MNI=2), sometimes associated with lithics
		re	6.1	littoral deposits, formed during ~300 years	E1 (19/1), E7 (6/2), E13 (6/2), E21 (33/2), E22 (160/4), E23 (108/3), E24 (256/8), E25 (7/4), E26 (20/1), E29 (5/2), E31 (11/2), E32 (5/1), E33 (5/1), E40 (2/1), E43 (87/3), C2 (10/2)	complete/partial skeletons, skeletal remains and individual bones of *Rhinoceros* sp. (MNI=9) and *B. primigenius* (MNI=9), partly associated with lithics, *M. giganteus* (MNI=1), *P. leo* (MNI=2), *C. crocuta* (MNI=1), *C. lupus* (MNI= 1), *U. arctos* (MNI=1), areas with lithics (n=~800) and bone fragments (NISP>1,400) of *P. antiquus, Rhinoceros* sp., *B. primigenius, Equus* sp., *D. dama, C. elaphus, Megaloceros* sp., *P. leo, Ursus* sp., *V. vulpes* (total MNI of these 23)
		tr	6	silts		
III	~ 450	*re*	5	denudation area		
		tr	4	gyttja	E5 (1/1), E6 (20/1)	complete/partial skeletons of *C. elaphus* (MNI=2)
II	~ 200	*re*	3	littoral deposits	.	
I	~ 100	*tr*		silts		

Neumark-Nord 2

unit	sediments	NN2 fauna and archaeology
18 17	sandy silts	NN2/1a: low-density scatters of lithics and bones
16	organic silts	NN2/1b: low-density scatters of lithics and bones
15	clay	
14	silts	
13	silts	
12	silts	
11	fine sands and silts, formed during <100 years	NN2/1c: Lithics and ~ 5,000 bones, *C. crocuta, P. antiquus, Equus* sp., *B. primigenius, D. dama, C. elaphus* (MNI=11)
10	silts	NN2/2A isolated scatters of individual lithics and bones
9	silts	
8	sandy silts. Formed during <455 years	NN2/2B: Lithics (n=~20,000) and bones (n=~120,000) of *P. leo, U. arctos, C. lupus, V. vulpes, P. antiquus, Rhinoceros* sp., *S. scrofa, Equus,* sp., *B. primigenius, C. capreolus., D. dama, C. elaphus, Alces* sp., *Megaloceros* sp.(all species MNI=166)
7	fine sands and silts	NN2/2C: low-density scatters lithics and bones
6	fine sands and silts	NN2/3: lithics (n=71) and bones (NISP=170) from *Equus* sp. (MNI=1) large *Cervid gen*. spec. indet. (MNI=1)
5	silts	
4	silts	

Table S15

The Neumark-Nord 1 elephant bone complexes in their geological and archaeological setting of the Neumark-Nord 1 and 2 sequences, with indicated Pollen Assemblage Zones (PAZ) of the Last Interglacial (*25*, *58*). for Neumark-Nord 1 and 2, the various lake phases of Neumark-Nord 1 (*tr* = transgression, r*e* = regression), the sedimentary context of the various find levels as well as information on the associated faunal and lithic finds (compiled after (*9*, *16–20*, *22*, *29*, *73*) The provenance of elephant bone complexes/assemblages analyzed is presented in a separate column (see also Table S1).

!

REFERENCES

1. A. Larramendi, M. R. Palombo, F. Marano, Reconstructing the life appearance of a Pleistocene giant: Size, shape, sexual dimorphism and ontogeny of *Palaeoloxodon antiquus* (Proboscidea: Elephantidae) from Neumark-Nord 1 (Germany). *Boll. Della Soc. Paleontol. Ital.* **56**, 299–317 (2017).

2. A. J. Stuart, The extinction of woolly mammoth (*Mammuthus primigenius*) and straight-tusked elephant (*Palaeoloxodon antiquus*) in Europe. *Quat. Int.* **126–128**, 171–177 (2005).

3. M. R. Palombo, E. Albayrak, F. Marano, The straight-tusked elephants from Neumark-Nord. A glance into a lost world, in *Elefantenreich: eine Fossilwelt in Europa ; Begleitband zur Sonderausstellung im Landesmuseum für Vorgeschichte Halle 26.03.-03.10.2010*, H. Meller, Ed. (Landesamt für Denkmalpflege und Archäologie Sachsen-Anhalt, Landesmuseum für Vorgeschichte, 2010), pp. 218–251.

4. E. Santucci, F. Marano, E. Cerilli, I. Fiore, C. Lemorini, M. R. Palombo, A. P. Anzidei, G. M. Bulgarelli, *Palaeoloxodon* exploitation at the Middle Pleistocene site of La Polledrara di Cecanibbio (Rome, Italy). *Quat. Int.* **406**, 169–182 (2016).

5. G. Haynes, Late Quaternary Proboscidean sites in Africa and Eurasia with possible or probable evidence for hominin involvement. *Quat.* **5**, 18 (2022).

6. K. D. Lupo, D. N. Schmitt, When bigger is not better: The economics of hunting megafauna and its implications for Plio-Pleistocene hunter-gatherers. *J. Anthropol. Archaeol.* **44**, 185–197 (2016).

7. W. Soergel, *Die Jagd der Vorzeit* (Gustav Fischer, 1922).

8. H. Thieme, S. Veil, Neue Untersuchungen zum eemzeitlichen Elefanten-Jagdplatz Lehringen, Ldkr. Verden. *Kunde* **36**, 11–58 (1985).

9. D. Mania, M. Thomae, T. Litt, T. Weber, Eds., *Neumark-Gröbern: Beiträge zur Jagd des mittelpaläolithischen Menschen* (Deutscher Verlag der Wissenschaften, 1990),

Veröffentlichungen des Landesmuseums für Vorgeschichte in Halle.

10. G. E. Konidaris, V. Tourloukis, Proboscidea-Homo interactions in open-air localities during the Early and Middle Pleistocene of western Eurasia: A palaeontological and archaeolocigal perspective, in *Human-Elephant Interactions: From Past to Present*, G. E. Konidaris, R. Barkai, V. Tourloukis, K. Harvati, Eds. (Tuebingen paleoanthropology book series - contributions in paleoanthropology, Tübingen Univ. Press, 2021), pp. 67–104; https://publikationen.uni-tuebingen.de/xmlui/handle/10900/114224.

11. G. Haynes, K. Krasinski, Butchering marks on bones of *Loxodonta africana* (African savanna elephant): Implications for interpreting marks on fossil proboscidean bones. *J. Archaeol. Sci. Rep.* **37**, 102957 (2021).

12. G. Haynes, J. Klimowicz, Recent elephant-carcass utilization as a basis for interpreting mammoth exploitation. *Quat. Int.* **359–360**, 19–37 (2015).

13. M. R. Palombo, E. Cerilli, Human-Elephant interactions during the Lower Palaeolithic: Scrutinizing the role of environmental factors, in *Human-Elephant Interactions: From Past to Present*, G. Konidaris, R. Barkai, V. Tourloukis, K. Harvati, Eds. (Tuebingen paleoanthropology book series - contributions in paleoanthropology, Tübingen Univ. Press, 2021), pp. 105–143; http://dx.doi.org/10.15496/publikation-55604.

14. H. Bocherens, D. G. Drucker, M. Germonpré, M. Lázničková-Galetová, Y. I. Naito, C. Wissing, J. Brůžek, M. Oliva, Reconstruction of the Gravettian food-web at Předmostí I using multi-isotopic tracking (^{13}C, ^{15}N, ^{34}S) of bone collagen. *Quat. Int.* **359–360**, 211–228 (2015).

15. J. Z. Metcalfe, Proboscidean isotopic compositions provide insight into ancient humans and their environments. *Quat. Int.* **443**, 147–159 (2017).

16. S. Gaudzinski-Windheuser, W. Roebroeks, Eds., *Multidisciplinary Studies of the Middle Palaeolithic Record from Neumark-Nord (Germany), Vol. I* (LDA-LSA, Halle (Saale), 2014), *Veröffentlichungen des Landesamtes für Denkmalpflege und Archäologie Sachsen-*

Anhalt - Landesmuseum für Vorgeschichte.

17. L. Kindler, G. M. Smith, A. Garcia-Moreno, S. Gaudzinski-Windheuser, E. Pop, W. Roebroeks, The last interglacial (Eemian) lakeland of Neumark-Nord (Saxony-Anhalt, Germany). Sequencing Neanderthal occupations, assessing subsistence opportunities and prey selection based on estimations of ungulate carrying capacities, biomass production and energy values, in *Human Behavioural Adaptations to Interglacial Lakeshore Environments*, A. Garcia-Moreno, J. M. Hutson, G. M. Smith, L. Kindler, E. Turner, A. Villaluenga, S. Gaudzinski-Windheuser, Eds. (RGZM-Tagungen, Propylaeum, 2020), pp. 67–104; https://books.ub.uni-heidelberg.de/index.php/propylaeum/catalog/book/647.

18. S. Gaudzinski-Windheuser, E. S. Noack, E. Pop, C. Herbst, J. Pfleging, J. Buchli, A. Jacob, F. Enzmann, L. Kindler, R. Iovita, M. Street, W. Roebroeks, Evidence for close-range hunting by last interglacial Neanderthals. *Nat. Ecol. Evol.* **2**, 1087–1092 (2018).

19. M. J. Sier, W. Roebroeks, C. C. Bakels, M. J. Dekkers, E. Brühl, D. De Loecker, S. Gaudzinski-Windheuser, N. Hesse, A. Jagich, L. Kindler, W. J. Kuijper, T. Laurat, H. J. Mücher, K. E. H. Penkman, D. Richter, D. J. J. van Hinsbergen, Direct terrestrial–marine correlation demonstrates surprisingly late onset of the last interglacial in central Europe. *Quat. Res.* **75**, 213–218 (2011).

20. D. Mania, H. Meller, Eds., *Neumark-Nord: ein interglaziales Ökosystem des mittelpaläolithischen Menschen* (LDA-LSA, Halle (Saale), 2010), *Veröffentlichungen des Landesamtes für Denkmalpflege und Archäologie Sachsen-Anhalt - Landesmuseum für Vorgeschichte*.

21. E. Pop, W. Kuijper, E. van Hees, G. Smith, A. García-Moreno, L. Kindler, S. Gaudzinski-Windheuser, W. Roebroeks, Fires at Neumark-Nord 2, Germany: An analysis of fire proxies from a Last Interglacial Middle Palaeolithic basin site. *J. Field Archaeol.* **41**, 603–617 (2016).

22. W. Roebroeks, K. MacDonald, F. Scherjon, C. Bakels, L. Kindler, A. Nikulina, E. Pop, S. Gaudzinski-Windheuser, Landscape modification by Last Interglacial Neanderthals. *Sci. Adv.*

7, eabj5567 (2021).

23. D. Mania, Der Fossilbericht von den Waldelefanten im Seebecken von Neumark-Nord, in *Elefantenreich: eine Fossilwelt in Europa ; Begleitband zur Sonderausstellung im Landesmuseum für Vorgeschichte Halle 26.03.-03.10.2010*, H. Meller, Ed. (Landesamt für Denkmalpflege und Archäologie Sachsen-Anhalt, Landesmuseum für Vorgeschichte, 2010), pp. 201–217.

24. F. Marano, M. R. Palombo, Population structure in straight-tusked elephants: A case study from Neumark Nord 1 (late Middle Pleistocene?, Sachsen-Anhalt, Germany). *Boll. Della Soc. Paleontol. Ital.* **52**, 207–218 (2013).

25. H. Müller, Pollenanalytische Untersuchungen und Jahresschichtenzählungen an der eem-zeitlichen Kieselgur von Bispingen/Luhe. *Geol. Jahrb.* **A21**, 149–169 (1974).

26. M. Meyer, E. Palkopoulou, S. Baleka, M. Stiller, K. E. H. Penkman, K. W. Alt, Y. Ishida, D. Mania, S. Mallick, T. Meijer, H. Meller, S. Nagel, B. Nickel, S. Ostritz, N. Rohland, K. Schauer, T. Schüler, A. L. Roca, D. Reich, B. Shapiro, M. Hofreiter, Palaeogenomes of Eurasian straight-tusked elephants challenge the current view of elephant evolution. *eLife* **6**, e25413 (2017).

27. R. L. Lyman, *Vertebrate Taphonomy* (Cambridge Manuals in Archaeology, Cambridge Univ. Press, 1994); http://site.ebrary.com/id/10897773.

28. G. Haynes, P. Wojtal, Weathering stages of proboscidean bones: Relevance for zooarchaeological analysis. *J. Archaeol. Method Theory* 10.1007/s10816-022-09569-3 (2022).

29. H. Meller, Ed., *Elefantenreich: eine Fossilwelt in Europa ; Begleitband zur Sonderausstellung im Landesmuseum für Vorgeschichte Halle 26.03.-03.10.2010* (Landesamt für Denkmalpflege und Archäologie Sachsen-Anhalt, Landesmuseum für Vorgeschichte, 2010).

30. G. E. Weissengruber, G. F. Egger, J. R. Hutchinson, H. B. Groenewald, L. Elsässer, D.

Famini, G. Forstenpointner, The structure of the cushions in the feet of African elephants (*Loxodonta africana*). *J. Anat.* **209**, 781–792 (2006).

31. B. M. Starkovich, P. Cuthbertson, K. Kitagawa, N. Thompson, G. E. Konidaris, V. Rots, S. C. Münzel, D. Giusti, V. C. Schmid, A. Blanco-Lapaz, C. Lepers, V. Tourloukis, Minimal tools, maximum meat: A pilot experiment to butcher an elephant foot and make elephant bone tools using lower paleolithic stone tool technology. *Ethnoarchaeology* **12**, 118–147 (2020).

32. G. Boschian, D. Caramella, D. Saccà, R. Barkai, Are there marrow cavities in Pleistocene elephant limb bones, and was marrow available to early humans? New CT scan results from the site of Castel di Guido (Italy). *Quat. Sci. Rev.* **215**, 86–97 (2019).

33. J. L. Guil-Guerrero, A. Tikhonov, R. P. Ramos-Bueno, S. Grigoriev, A. Protopopov, G. Savvinov, M. J. González-Fernández, Mammoth resources for hominins: From omega-3 fatty acids to cultural objects. *J. Quat. Sci.* **33**, 455–463 (2018).

34. S. E. Churchill, Weapon technology, prey size selection, and hunting methods in modern hunter-gatherers: Implications for hunting in the Palaeolithic and Mesolithic. *Archeol. Pap. Am. Anthropol. Assoc.* **4**, 11–24 (1993).

35. A. Milks, A review of ethnographic use of wooden spears and implications for pleistocene hominin hunting. *Open Quat.* **6**, 12 (2020).

36. H. Thieme, Lower Palaeolithic hunting spears from Germany. *Nature* **385**, 807–810 (1997).

37. J. D. Speth, K. A. Spielmann, Energy source, protein metabolism, and hunter-gatherer subsistence strategies. *J. Anthropol. Archaeol.* **2**, 1–31 (1983).

38. J. D. Speth, *The Paleoanthropology and Archaeology of Big-Game Hunting* (Interdisciplinary Contributions to Archaeology, Springer, 2010; http://link.springer.com/10.1007/978-1-4419-6733-6.

39. R. Boyd, P. J. Richerson, Large-scale cooperation in small-scale foraging societies. *Evol.*

Anthropol. **31**, 175–198 (2022).

40. D. W. Bird, R. B. Bird, B. F. Codding, D. W. Zeanah, Variability in the organization and size of hunter-gatherer groups: Foragers do not live in small-scale societies. *J. Hum. Evol.* **131**, 96–108 (2019).

41. R. L. Kelly, *The Lifeways of Hunter-Gatherers: The Foraging Spectrum* (Cambridge Univ. Press, ed. 2, 2013).

42. S. E. Churchill, *Thin on the Ground: Neandertal Biology, Archeology, and Ecology* (John Wiley & Sons Inc., 2014); http://doi.wiley.com/10.1002/9781118590836.

43. M. Ichikawa, Elephant hunting by the Mbuti hunter-gatherers in the Eastern Congo Basin, in *Human-Elephant Interactions: From Past to Present*, G. Konidaris, R. Barkai, V. Tourloukis, K. Harvati, Eds. (Tuebingen paleoanthropology book series - contributions in paleoanthropology, Tübingen Univ. Press, 2021), pp. 455–467; http://dx.doi.org/10.15496/publikation-55604.

44. B. Bratlund, Taubach revisited. *Jahrb. Röm. Ger. Zentralmuseums.* **46**, 61–174 (1999).

45. S. Gaudzinski, W. Roebroeks, Adults only. Reindeer hunting at the Middle Palaeolithic site Salzgitter Lebenstedt, Northern Germany. *J. Hum. Evol.* **38**, 497–521 (2000).

46. P. Auguste, Chasse et charognage au Paléolithique moyen: L'apport du gisement de Biache-Saint-Vaast (Pas-de-Calais). *Bull. Société Préhistorique Fr.* **92**, 155–168 (1995).

47. L. Kindler, *Die Rolle von Raubtieren bei der Einnischung und Subsistenz jungpleistozäner Neandertaler: Archäozoologie und Taphonomie der mittelpaläolithischen Fauna aus der Balver Höhle, Westfalen* (Verl. des Römisch-Germanischen Zentralmuseums, 2012).

48. J. Speth, J. Clark, Hunting and overhunting in the Levantine Late Middle Palaeolithic. *Farming* **2006**, 1–42 (2006).

49. J. M. Hutson, A. Villaluenga, A. García-Moreno, E. Turner, S. Gaudzinski-Windheuser, A

zooarchaeological and taphonomical perspective of hominin behaviour from the Schöningen 13II-4 “Spear Horizon”, in *Human Behavioural Adaptations to Interglacial Lakeshore Environments* (RGZM-Tagungen, Propylaeum, 2020), pp. 43–66; https://books.ub.uni-heidelberg.de/index.php/propylaeum/catalog/book/647.

50. A. García-Moreno, J. M. Hutson, A. Villaluenga, E. Turner, S. Gaudzinski-Windheuser, A detailed analysis of the spatial distribution of Schöningen 13II-4 ‘Spear Horizon’ faunal remains. *J. Hum. Evol.* **152**, 102947 (2021).

51. G. L. Dusseldorp, *A View to a kill: Investigating Middle Palaeolithic Subsistence Using an Optimal Foraging Perspective* (Sidestone Press, 2009).

52. M. M. Smuts, A. J. Bezuidenhout, Osteology of the thoracic limb of the African elephant (Loxodonta africana). *Onderstepoort J. Vet. Res.* **60**, 1–14 (1993).

53. A. J. Bezuidenhout, C. D. Seegers, The osteology of the African elephant (Loxodonta africana): Vertebral column, ribs and sternum. *Onderstepoort J. Vet. Res.* **63**, 131–147 (1996).

54. N. J. van der Merwe, A. J. Bezuidenhout, C. D. Seegers, The skull and mandible of the African elephant (Loxodonta africana). *Onderstepoort J. Vet. Res.* **62**, 245–260 (1995).

55. G. J. Stanek, thesis, Veterinärmedizinische Universität Wien, Wien (2012).

56. R. G. Klein, K. Cruz-Uribe, *The Analysis of Animal Bones From Archeological Sites* (Prehistoric archeology and ecology, University of Chicago Press, 1984).

57. Y. Fernández-Jalvo, P. Andrews, *Atlas of Taphonomic Identifications 1001+ Images of Fossil and Recent Mammal Bone Modification* (Vertebrate Paleobiology and Paleoanthropology Series, Springer, 2016); http://springerlink.com/content/978-94-017-7432-1.

58. B. Menke, R. Tynni, Das Eeminterglazial und das Weichselfrühglazial von Rederstall/Dithmarschen und ihre Bedeutung für die mitteleuropäische Jungpleistozän-

Gliederung. *Geol. Jahrb.* **A76**, 3–120 (1984).

59. R. Grube, M. R. Palombo, P. Iacumin, A. Di Matteo, What did the fossil elephants from Neumark-Nord eat?, in *Elefantenreich: eine Fossilwelt in Europa; Begleitband zur Sonderausstellung im Landesmuseum für Vorgeschichte Halle 26.03.-03.10.2010*, H. Meller, Ed. (Landesamt für Denkmalpflege und Archäologie Sachsen-Anhalt, Landesmuseum für Vorgeschichte, 2010), pp. 252–274.

60. J. Koller, U. Baumer, Der organische Belag auf der Silexklinge aus Neumark-Nord. Gerbungsmaterial oder Schäftungskit?, in *Elefantenreich: eine Fossilwelt in Europa ; Begleitband zur Sonderausstellung im Landesmuseum für Vorgeschichte Halle 26.03.-03.10.2010*, H. Meller, Ed. (Landesamt für Denkmalpflege und Archäologie Sachsen-Anhalt, Landesmuseum für Vorgeschichte, 2010), pp. 553–563.

61. D. A. Byers, A. Ugan, Should we expect large game specialization in the late Pleistocene? An optimal foraging perspective on early Paleoindian prey choice. *J. Archaeol. Sci.* **32**, 1624–1640 (2005).

62. G. C. Frison, L. C. Todd, *The Colby Mammoth Site: Taphonomy and Archaeology of a Clovis Kill in Northern Wyoming* (University of New Mexico Press, ed. 1, 1986).

63. S. Bilsborough, N. Mann, A review of issues of dietary protein intake in humans. *Int. J. Sport Nutr. Exerc. Metab.* **16**, 129–152 (2006).

64. L. Cordain, J. B. Miller, S. B. Eaton, N. Mann, S. H. Holt, J. D. Speth, Plant-animal subsistence ratios and macronutrient energy estimations in worldwide hunter-gatherer diets. *Am. J. Clin. Nutr.* **71**, 682–692 (2000).

65. R. S. Kuipers, M. F. Luxwolda, D. A. Janneke Dijck-Brouwer, S. B. Eaton, M. A. Crawford, L. Cordain, F. A. J. Muskiet, Estimated macronutrient and fatty acid intakes from an East African Paleolithic diet. *Br. J. Nutr.* **104**, 1666–1687 (2010).

66. D. E. Chusyd, J. L. Brown, C. Hambly, M. S. Johnson, K. Morfeld, A. Patki, J. R. Speakman, D. B. Allison, T. R. Nagy, Adiposity and reproductive cycling status in zoo african elephants.

Obesity **26**, 103–110 (2018).

67. D. E. Chusyd, T. R. Nagy, L. Golzarri-Arroyo, S. L. Dickinson, J. R. Speakman, C. Hambly, M. S. Johnson, D. B. Allison, J. L. Brown, Adiposity, reproductive and metabolic health, and activity levels in zoo Asian elephant (*Elephas maximus*). *J. Exp. Biol.* **224**, jeb219543 (2021).

68. D. C. Salazar-García, R. C. Power, A. Sanchis Serra, V. Villaverde, M. J. Walker, A. G. Henry, Neanderthal diets in central and southeastern Mediterranean Iberia. *Quat. Int.* **318**, 3–18 (2013).

69. R. C. Power, D. C. Salazar-García, M. Rubini, A. Darlas, K. Harvati, M. Walker, J.-J. Hublin, A. G. Henry, Dental calculus indicates widespread plant use within the stable Neanderthal dietary niche. *J. Hum. Evol.* **119**, 27–41 (2018).

70. J. A. Fellows Yates, I. M. Velsko, F. Aron, C. Posth, C. A. Hofman, R. M. Austin, C. E. Parker, A. E. Mann, K. Nägele, K. W. Arthur, J. W. Arthur, C. C. Bauer, I. Crevecoeur, C. Cupillard, M. C. Curtis, L. Dalén, M. Díaz-Zorita Bonilla, J. C. Díez Fernández-Lomana, D. G. Drucker, E. Escribano Escrivá, M. Francken, V. E. Gibbon, M. R. González Morales, A. Grande Mateu, K. Harvati, A. G. Henry, L. Humphrey, M. Menéndez, D. Mihailović, M. Peresani, S. Rodríguez Moroder, M. Roksandic, H. Rougier, S. Sázelová, J. T. Stock, L. G. Straus, J. Svoboda, B. Teßmann, M. J. Walker, R. C. Power, C. M. Lewis, K. Sankaranarayanan, K. Guschanski, R. W. Wrangham, F. E. Dewhirst, D. C. Salazar-García, J. Krause, A. Herbig, C. Warinner, The evolution and changing ecology of the African hominid oral microbiome. *Proc. Natl. Acad. Sci. U.S.A.* **118**, e2021655118 (2021).

71. A. G. Henry, A. S. Brooks, D. R. Piperno, Plant foods and the dietary ecology of Neanderthals and early modern humans. *J. Hum. Evol.* **69**, 44–54 (2014).

72. W. J. Kuijper, Investigation of inorganic, botanical, and zoological remains of an exposure of Last Interglacial (Eemian) sediments at Neumark-Nord 2 (Germany), in *Multidisciplinary Studies of the Middle Palaeolithic Record from Neumark-Nord (Germany), Vol. I*, S. Gaudzinski-Windheuser, W. Roebroeks, Eds. (Veröffentlichungen des Landesamtes für

Denkmalpflege und Archäologie Sachsen-Anhalt - Landesmuseum für Vorgeschichte, LDA-LSA, 2014), pp. 79–97.

73. E. Pop, C. Bakels, Semi-open environmental conditions during phases of hominin occupation at the Eemian Interglacial basin site Neumark-Nord 2 and its wider environment. *Quat. Sci. Rev.* **117**, 72–81 (2015).
